# An Activated Dendritic-Cell-Related Gene Signature Indicative of Disease Prognosis and Chemotherapy and Immunotherapy Response in Colon Cancer Patients

**DOI:** 10.3390/ijms242115959

**Published:** 2023-11-03

**Authors:** Yiben Ouyang, Mingqian Yu, Tiange Liu, Mengying Suo, Jingyi Qiao, Liqiang Wang, Na Li

**Affiliations:** School of Medicine, Nankai University, Tianjin 300071, China; ed1501260@163.com (Y.O.); mingqianyu2022@163.com (M.Y.); nimbus2000e@163.com (T.L.); 15866725760@163.com (M.S.); 18092289178@163.com (J.Q.)

**Keywords:** colon cancer, activated dendritic cells, gene signature, nomogram, prognosis, chemotherapy and immunotherapy response, cancer biomarkers

## Abstract

Accumulating evidence has underscored the prognostic value of tumor-infiltrating immune cells in the tumor microenvironment of colon cancer (CC). In this retrospective study, based on publicly available transcriptome profiles and clinical data from the Gene Expression Omnibus and The Cancer Genome Atlas databases, we derived and verified an activated dendritic cell (aDC)-related gene signature (aDCRS) for predicting the survival outcomes and chemotherapy and immunotherapy response of CC patients. We quantified the infiltration abundance of 22 immune cell subtypes via the “CIBERSORT” R script. Univariate Cox proportional hazards (PHs) regression was used to identify aDC as the most robust protective cell type for CC prognosis. After selecting differentially expressed genes (DEGs) significantly correlated with aDC infiltration, we performed univariate Cox-PH regression, LASSO regression, and stepwise multivariate Cox-PH regression successively to screen out prognosis-related genes from selected DEGs for constructing the aDCRS. Receiver operating characteristic (ROC) curves and Kaplan–Meier (KM) analysis were employed to assess the discriminatory ability and risk-stratification capacity. The “oncoPredict” package, Cancer Treatment Response gene signature DataBase, and Tumor Immune Dysfunction and Exclusion algorithm were utilized to estimate the practicability of the aDCRS in predicting response to chemotherapy and immune checkpoint blockade. Gene set enrichment analysis and single-cell RNA sequencing analysis were also implemented. Furthermore, an aDCRS-based nomogram was constructed and validated via ROC curves, calibration plots and decision curve analysis. In conclusion, aDCRS and an aDCRS-based nomogram will facilitate precise prognosis prediction and individualized therapeutic interventions, thus improving the survival outcomes of CC patients in the future.

## 1. Introduction

Colorectal cancer (CRC) remains the third most prevalent cancer and the second leading cause of cancer-related deaths globally according to the GLOBOCAN 2020 estimates [1]. Approximately 50% of patients with localized lesions eventually fall into metastatic CRC [2]. Current treatment decisions for CRC sufferers primarily rely on histopathological assessment of tumor invasion degree according to the American Joint Committee on Cancer/Union Internationale Contre le Cancer (AJCC/UICC) TNM classification system [3,4]. Nevertheless, this system has limited prognostic value to predict response to treatment, with considerably fluctuating risks of CRC relapse and mortality even among patients with similar histopathological tumor stage [5,6,7]. The core issue lies in an inaccurate assumption of this traditional staging system, which claims that disease progression is a tumor cell-autonomous process, and thereby confines the main focus to tumor cells themselves, rather than the reciprocal and dynamic interactions between tumor cells and their adjacent tumor microenvironment (TME) [5,8].

Initiated by the stepwise accumulation of genetic and epigenetic changes, the evolution of the CRC niche is driven by both consecutive replication of dysregulated epithelial cells lining colon mucosa and an accomplice branded TME. TME is an admixture of cellular components mainly comprising immune and inflammatory cells, cancer-associated fibroblasts, and the blood and lymphatic vascular networks, as well as non-cellular components including extracellular matrix [9,10]. The colorectal stroma, which physiologically serves as a defender of the equilibrium and homeostasis in normal tissues, now becomes exposed to frequent education, cooption, and modification from the cancer cells and thereby synthesizes a wide variety of cytokines, chemokines, proteinases, and growth factors [9,11]. All of these components interact reciprocally and can concertedly regulate the proliferation, cell death, immunosuppression, and energy metabolism of tumor cells, thus checking the tumorigenesis, progression, and metastasis of CRC as well as determining treatment response and drug resistance [12].

As the major TME stromal cellular constituents, immune cells aggregate into the tumor-adjacent milieu and exert profound influence on clinical outcomes [9]. As a heterogeneous and rare type of immune cells, dendritic cells (DCs) are professional antigen-presenting cells that orchestrate innate and adaptive immune responses through a developmental process termed “maturation” [13,14]. Inherently, DCs exist in two phenotypically and functionally discrepant states, immature and mature, which can exert distinct impacts on CRC progression. Immature DCs are highly adept at antigen uptake, but are less proficient in antigen presentation and may induce immune tolerance and immune escape that can lead to further progression of CRC. Mature DCs have a reduced capacity for antigen uptake but obtain an exceptional capacity for T cell stimulation at this stage [13]. Most DCs in peripheral tissues are of the immature phenotype, which expresses relatively low levels of surface costimulatory molecules (CD40, CD70, CD80, CD86, etc.) [13,15]. In response to microbial or inflammatory stimuli, immature DCs are activated and transform into mature DCs, which migrate from peripheral tissues to secondary lymphoid organs, present captured antigens to naïve T cells, and ultimately prime naïve T cells to initiate adaptive immunity. Meanwhile, the expression of costimulatory molecules and production of inflammatory cytokines are all elevated [13,16]. Distinct subsets of DCs with specific phenotype and maturation status are involved in different immune responses. Therefore, the presence or absence of DCs and their explicit subtype, as well as activation/maturation status, are robustly correlated with different survival outcomes [17,18]. An enhanced infiltration of activated and mature DCs in CRC can improve overall survival (OS) in CRC patients, suggesting the important role of DCs in tumor progression [17,19,20].

Considering DCs’ prognostic utility, tumor cell-oriented therapeutic strategies should be integrated with specific interventions that modify pre- and post-operative immune responses mediated by DCs to achieve ideal clinical outcomes [17]. In this study, we identified activated DC (aDC) infiltration as the most robust protective factor for colon cancer (CC) prognosis among 22 immune cell types. Differentially expressed genes (DEGs) that are significantly correlated with both aDC infiltration abundance and OS probabilities were selected through a sophisticated procedure and incorporated into an aDC-related gene signature (aDCRS). The aDCRS exhibited extraordinary discriminatory ability and risk-stratification capacity, both for predicting patient survival and for forecasting response to chemotherapy and immune checkpoint blockade (ICB). Ultimately, a prognostic nomogram was formulated anchored in the aDCRS and clinicopathological features to improve the predictive accuracy and efficiency. Our results shed light on how DC activation and infiltration shape the immune pathogenesis of CC and can assist clinicians to make precise predictions of patient prognosis and select appropriate therapeutic strategies for each patient.

## 2. Results

### 2.1. Estimation of Immune Cell Infiltration Abundance and Their Prognostic Value

**Figure 1:**
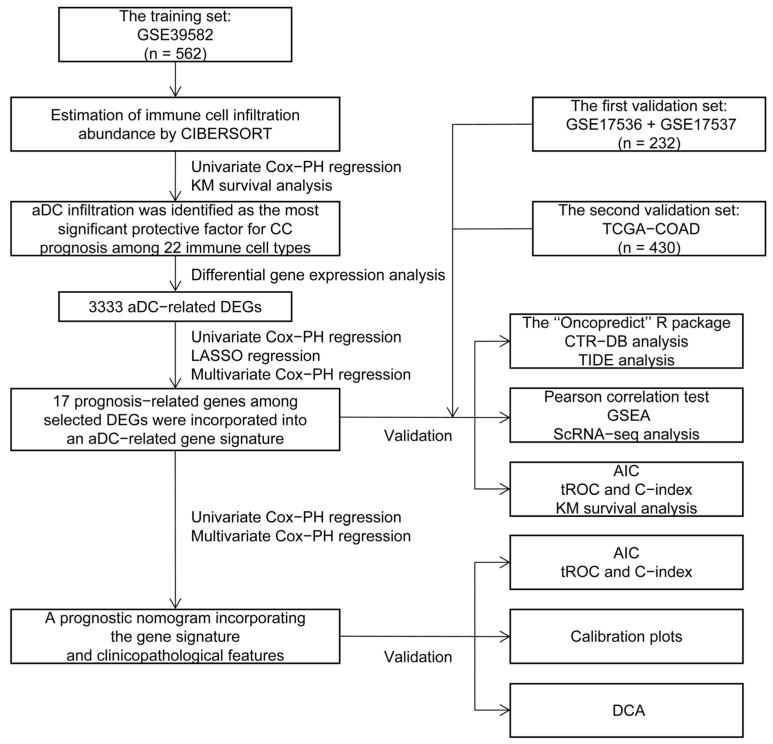
The study flowchart. CIBERSORT: Cell-type Identification by Estimating Relative Subsets of RNA Transcripts; PH: proportional hazards; KM: Kaplan–Meier; aDC: activated dendritic cell; CC: colon cancer; DEGs: differentially expressed genes; LASSO: least absolute shrinkage and selection operator; TCGA: The Cancer Genome Atlas; COAD: colon adenocarcinoma; CTR-DB: Cancer Treatment Response gene signature DataBase; TIDE: Tumor Immune Dysfunction and Exclusion; GSEA: Gene Set Enrichment Analysis; ScRNA-seq: single-cell RNA sequencing; AIC: Akaike information criterion; tROC: time-dependent receiver operating characteristic; C-index: concordance index; DCA: decision curve analysis.

**Figure 2:**
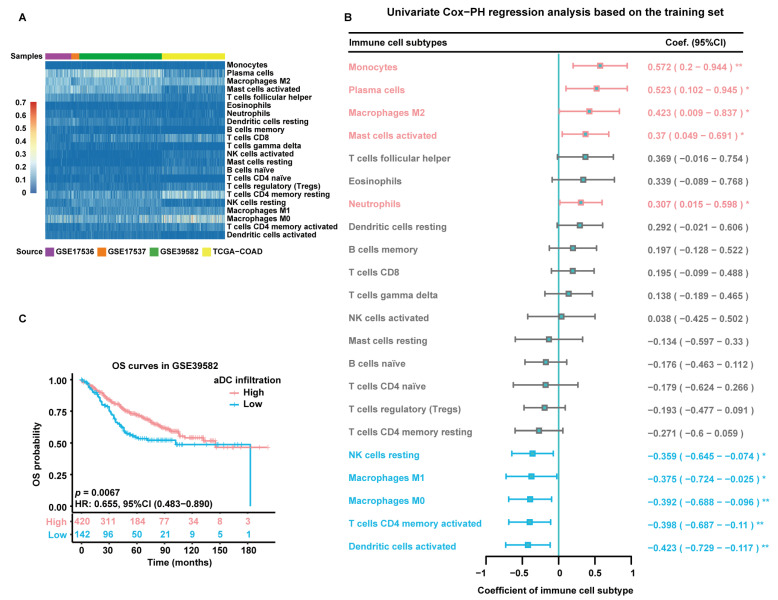
Estimation of immune cell infiltration abundance and their prognostic value. (**A**) Quantification of immune cell infiltration via CIBERSORT analysis of gene expression data from all enrolled tumor samples. The heatmap displays the infiltration abundance of 22 immune cell subtypes in each sample. The color intensity shown in the left column is proportional to the CIBERSORT score. (**B**) A forest plot exhibiting results of univariate Cox-PH regression analysis based on 562 patients in the training set. Cell types are marked in red to represent risk factors and blue to represent protective factors. *: *p* < 0.05; **: *p* < 0.01. (**C**) KM survival curves of patients in the high- and low-aDC-infiltration groups based on 562 patients in the training set. Individual survival numbers and time data are labeled at the bottom.

The flowchart of this study is depicted in Figure 1. The infiltration abundance of 22 immune cell subtypes in all enrolled tumor samples was appraised via the Cell-type Identification by Estimating Relative Subsets of RNA Transcripts (CIBERSORT) R script and displayed in a heatmap (Figure 2A). The detailed CIBERSORT scores, *p*-values, and Pearson correlation coefficients corresponding to each sample from 562 CC patients in the training set (GSE39582) are demonstrated in Figure S1. As revealed by univariate Cox proportional hazards (PHs) regression analysis (Figure 2B), among the 22 investigated immune cell types, the CIBERSORT scores of resting NK cells, M1-type macrophages, M0-type macrophages, activated CD4+ memory T cells, and aDCs show positive correlation with the OS status of CC patients, with aDCs exhibiting the strongest protective effect. Meanwhile, monocytes, plasma cells, M2-type macrophages, activated mast cells, and neutrophils are all significant unfavorable factors for CC prognosis. Kaplan–Meier (KM) analysis with log-rank statistical tests unearthed that patients with higher aDC infiltration showed better OS compared to those with lower aDC infiltration (Figure 2C). We also categorized the 562 patients according to the optimal cutoff values of the CIBERSORT scores of other immune cells that had significant association with CC prognosis. The results of the corresponding KM analyses are displayed in Figure S2.

### 2.2. Selection of Candidate Genes and Construction of the Prognostic Gene Signature

**Figure 3:**
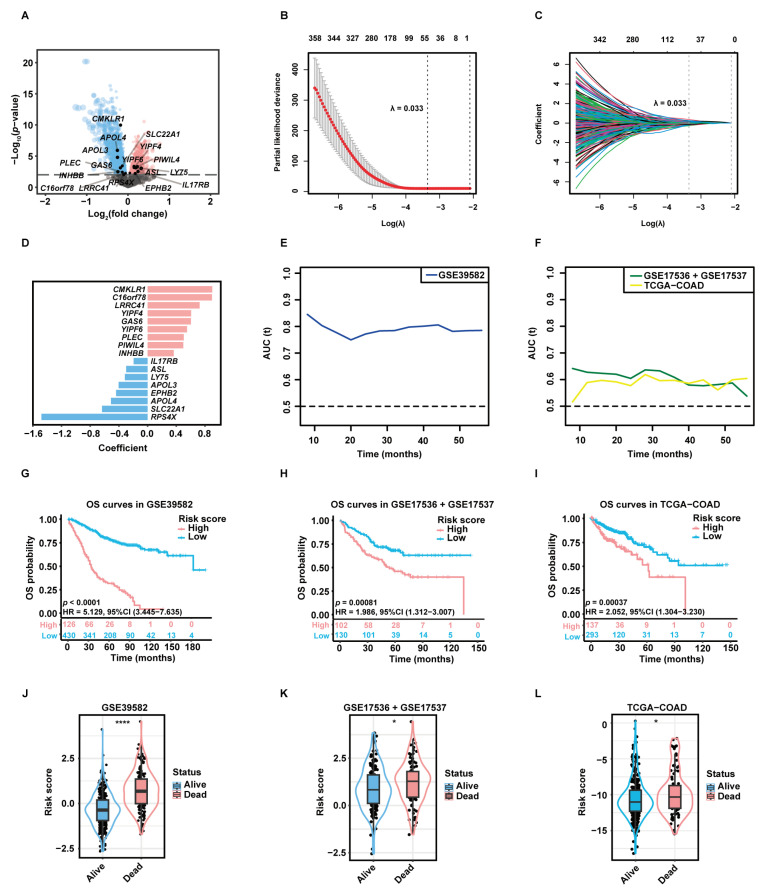
Construction and preliminary validation of the prognostic gene signature. (**A**) The volcano plot exhibiting DEGs between the high- and low-aDC-infiltration groups. Up-regulated genes and down-regulated genes in the high-aDC-infiltration group are marked in red and blue, respectively. (**B**) Tuning parameter (λ) selection of deviance in LASSO regression analysis. The red dot represents the mean cross-validated error (CVM), and the gray line represents the standard error of CVM. (**C**) LASSO coefficient profiles of 812 candidate genes selected by univariate Cox-PH regression analysis. Each curve in different color represents the trajectory of the coefficient of each variable. (**D**) The coefficient for each aDC-related prognostic gene (aDCRG). Genes are marked in red to represent risk factors and blue to represent protective factors. (**E**,**F**) The tROC curves demonstrating the discriminatory ability of the aDC-related gene signature (aDCRS) for predicting OS probabilities in the training set (**E**) and two validation sets (**F**). (**G**–**I**) KM survival curves of patients in the high- and low-risk groups based on the training set (**G**) and two validation sets (**H**,**I**). Numbers of individuals alive at different time points are presented at the bottom. (**J**–**L**) Comparison of the risk scores between alive and deceased patients in the training set (**J**) and two validation sets (**K**,**L**). Statistical significance was determined by Wilcoxon–Mann–Whitney test for the training set and the second validation set, or by Student’s *t*-test for the first validation set. AUC (t): the areas under the curve at different time points. *: *p* < 0.05; ****: *p* < 0.0001.

A total of 3333 aDC-related DEGs, including 1581 up-regulated genes and 1752 down-regulated genes, were screened out in the high-aDC-infiltration group (Figure 3A). Among these genes, 812 candidate genes were associated with the OS rates of CC patients as uncovered by the univariate Cox-PH analysis. The least absolute shrinkage and selection operator (LASSO) regression retained 53 genes with individual nonzero LASSO coefficients when the optimal λ value was 0.033 (Figure 3B,C). Subsequently, the stepwise multivariate Cox-PH regression was employed to identify optimal prognostic markers and construct a gene signature termed “aDCRS”, which was a risk score model composed of 17 aDC-related prognostic genes (aDCRGs): *CMKLR1*, *C16orf78*, *LRRC41*, *YIPF4*, *GAS6*, *YIPF6*, *PLEC*, *PIWIL4*, *INHBB*, *IL17RB*, *ASL*, *LY75*, *APOL3*, *EPHB2*, *APOL4*, *SLC22A1* and *RPS4X*. The coefficient for each aDCRG is displayed in Figure 3D. Furthermore, there are significant discrepancies in the mRNA levels of most of these genes between tumor tissues and adjacent normal tissues based on the training set (Figure S3A) and the second validation set (TCGA-COAD, Figure S3B).

(1) Risk score=0.902×Exp(CMKLR1)+0.897×Exp(C16orf78)+0.724×Exp(LRRC41)+0.606×Exp(YIPF4)+0.604×Exp(GAS6)+0.550×Exp(YIPF6)+0.503×Exp(PLEC)+0.498×Exp(PIWIL4)+0.364×Exp(INHBB)−0.195×Exp(IL17RB)−0.299×Exp(ASL)−0.318×Exp(LY75)−0.405×Exp(APOL3)−0.439×Exp(EPHB2)−0.512×Exp(APOL4)−0.636×Exp(SLC22A1)−1.481×Exp(RPS4X).

### 2.3. Validation of the Discriminatory Ability and Risk-Stratification Capacity of the aDCRS in Predicting CC Prognosis

The time-dependent receiver operating characteristic (tROC) curves of the aDCRS based on the training set (Figure 3E) and two validation sets (Figure 3F) were delineated for OS probabilities. The Akaike information criterion (AIC), concordance index (C-index) and bootstrapping-corrected C-index values were calculated as 2024.739, 0.754, and 0.734, respectively, and the 1-year, 3-year, and 5-year area under the curve (AUC) values were computed as 0.803, 0.798, and 0.782, respectively, based on the training set, indicating that the discriminatory ability of the signature is reliable and stable over time.

To assess the risk-stratification capacity of the aDCRS, we divided CC patients into two groups according to the optimal cutoff value of their risk scores. KM analysis with log-rank tests disclosed that OS probabilities were significantly higher in low-risk patients compared to those in high-risk patients in the training set (Figure 3G), the first validation set (GSE17536 + GSE17537, Figure 3H), the second validation set (Figure 3I), subset GSE17536 (Figure S3C), and subset GSE17537 (Figure S3D).

We further divided CC patients into the alive group and the dead group and compared their risk scores. Patients who remained alive during follow-up had significantly lower risk scores than deceased patients in the training set (Figure 3J) and two validation sets (Figure 3K–L). These results illuminated that the aDCRS has convincing discriminatory power and risk-stratification function in predicting CC prognosis.

### 2.4. Validation of the Pertinence between the aDCRGs and DC Activation and Infiltration and Exploration of Underlying Mechanisms

**Figure 4:**
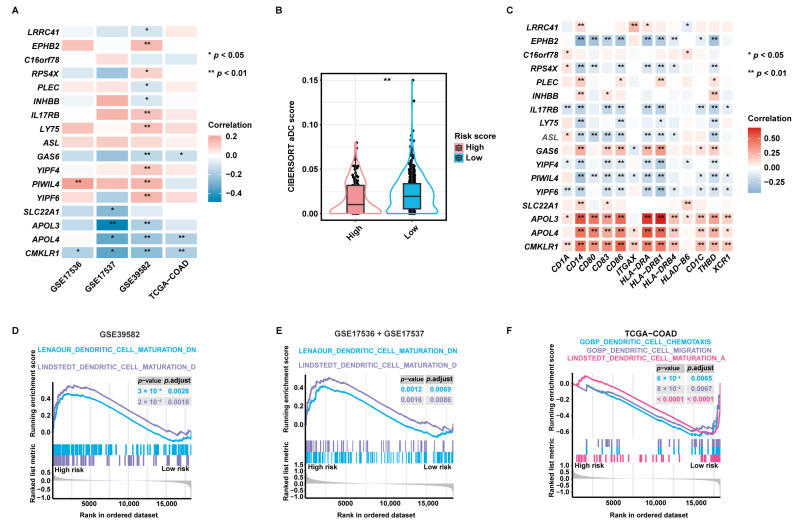
Validation of the pertinence between aDCRGs and DC activation and infiltration and exploration of underlying mechanisms. (**A**) Pearson correlation analysis between aDCRGs and aDC infiltration abundance in the training and validation sets. *: *p* < 0.05; **: *p* < 0.01. (**B**) Comparison of CIBERSORT aDC scores between the high- and low-risk groups using the Wilcoxon–Mann–Whitney test. (**C**) Pearson correlation analysis between aDCRGs and aDC/DC markers in the training set. *: *p* < 0.05; **: *p* < 0.01. (**D**–**F**) DC-relevant enriched gene sets in the high- or low-risk group as probed using GSEA based on the training set (**D**), the first validation set (**E**) and the second validation set (**F**). *p*.adjust: adjusted *p*-value.

Pearson correlation analysis disclosed that 14 of 17 aDCRGs were significantly associated with CIBERSORT aDC scores based on the training set (Figure 4A). *LRRC41*, *PLEC*, *INHBB*, *GAS6*, and *CMKLR1*, which were identified as unfavorable factors for OS by multivariate Cox-PH regression analysis, were shown to be inversely correlated with aDC infiltration, whereas *EPHB2*, *RPS4X*, *IL17RB*, and *LY75*, which were recognized as protective factors for patient survival, were demonstrated to be positively correlated with CIBERSORT aDC scores. Moreover, the infiltration abundance of aDCs was significantly lower in the high-risk group than that in the low-risk group (Figure 4B). Furthermore, each aDCRG had significant correlation with aDC/DC markers (Figure 4C). These results further corroborate the potential function of aDCRGs in DC activation and infiltration.

Additionally, Gene Set Enrichment Analysis (GSEA) based on DC-relevant gene sets uncovered that the DC maturation down-regulation pathway was enriched in the high-risk group in the training set (Figure 4D) and the first validation set (Figure 4E). The DC maturation activation pathway, the DC chemotaxis pathway, and the DC migration pathway were down-regulated in the high-risk group in the second validation set (Figure 4F). These results elucidate that the suppression of DC maturation, chemotaxis, and migration may be the mechanistic underpinning of the poor survival outcomes of CC patients in the high-risk group. Enrichment results with significant differences between the high- and low-risk groups as proved by GSEA based on hallmark gene sets are demonstrated in Figure S3E.

### 2.5. Validation of the Predictive Value of the aDCRS in Chemotherapy and Immunotherapy Response

**Figure 5:**
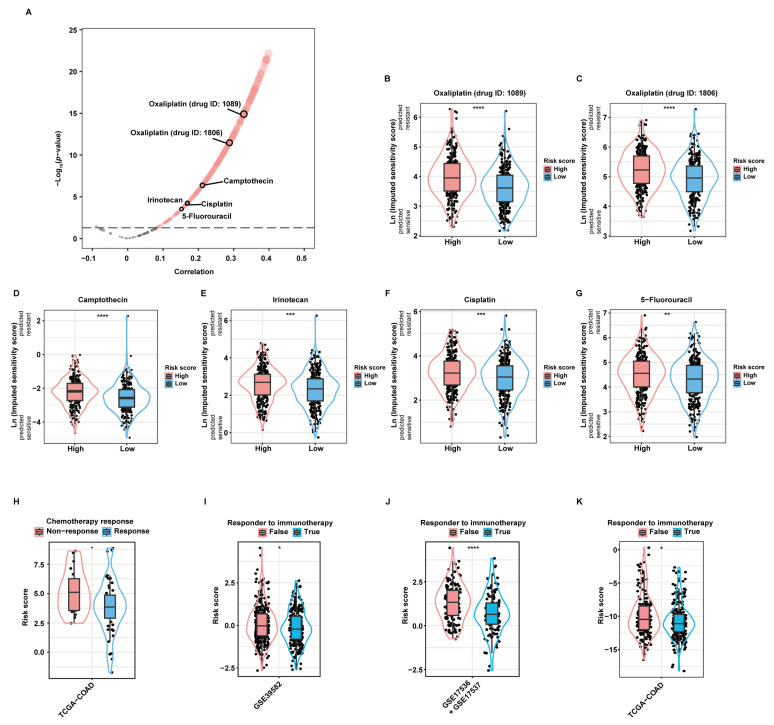
Validation of the practicability of the aDCRS in predicting chemotherapy and immunotherapy response. (**A**) A volcano plot depicting the results of Pearson correlation analysis between the risk score and the imputed sensitivity scores of anticancer drugs generated by the “Oncopredict” package based on 562 patients in the training set. Cor: correlation coefficient. (**B**–**G**) Violin plots depicting the imputed sensitivity score of Oxaliplatin (drug ID: 1089, (**B**)), Oxaliplatin (drug ID: 1806, (**C**)), Camptothecin (**D**), Irinotecan (**E**), Cisplatin (**F**), and 5-Fluorouracil (**G**) in high- and low-risk patients. A higher imputed sensitivity score indicates increased resistance. Statistical significance was determined by Wilcoxon–Mann–Whitney test for Camptothecin, or by Student’s *t*-test for the other drugs. (**H**) Comparison of the risk scores between the response and non-response groups using Student’s *t*-test based on 67 CC patients in the TCGA-COAD dataset obtained from the CTR-DB. (**I**–**K**) Comparison of the risk scores between the response and non-response groups based on the training set (**I**) and two validation sets (**J**,**K**) using TIDE analysis. Statistical significance was determined by Wilcoxon–Mann–Whitney test in the training set and the second validation set, or by Student’s *t*-test in the first validation set. *: *p* < 0.05; **: *p* < 0.01; ***: *p* < 0.001; ****: *p* < 0.0001.

As revealed by the “oncoPredict” package, there was significant correlation between the risk score and the imputed sensitivity scores of many chemotherapeutic drugs (Figure 5A). Among them, Oxaliplatin (drug ID: 1089; Figure 5B), Oxaliplatin (drug ID: 1806; Figure 5C), Camptothecin (Figure 5D), Irinotecan (Figure 5E), Cisplatin (Figure 5F), and 5-Fluorouracil (5-FU; Figure 5G), most of which are first- or second-line chemotherapeutic drugs when used alone or in combination recommended by current guidelines [21], were predicted to yield significantly higher therapeutic resistance in high-risk patients than in low-risk patients. Additionally, we applied the aDCRS to 67 CC patients in the TCGA-COAD dataset, whose treatment information was collected and curated by the Cancer Treatment Response gene signature DataBase (CTR-DB). The corresponding CTR-DB IDs, chemotherapeutic regimens, and response to treatment are depicted in Table S1. Higher risk scores were observed in the non-response group compared with those in the response group (Figure 5H), suggesting the prominent capacity of the aDCRS in predicting chemotherapy response in CC patients.

Subsequently, Tumor Immune Dysfunction and Exclusion (TIDE) analysis disclosed that responders to ICB therapies had lower risk scores than non-responders in the training set (Figure 5I) and two validation sets (Figure 5J,K), indicating the good utility of the aDCRS in forecasting ICB response.

### 2.6. Single-Cell RNA Sequencing (ScRNA-Seq) Analysis of aDCRGs

**Figure 6:**
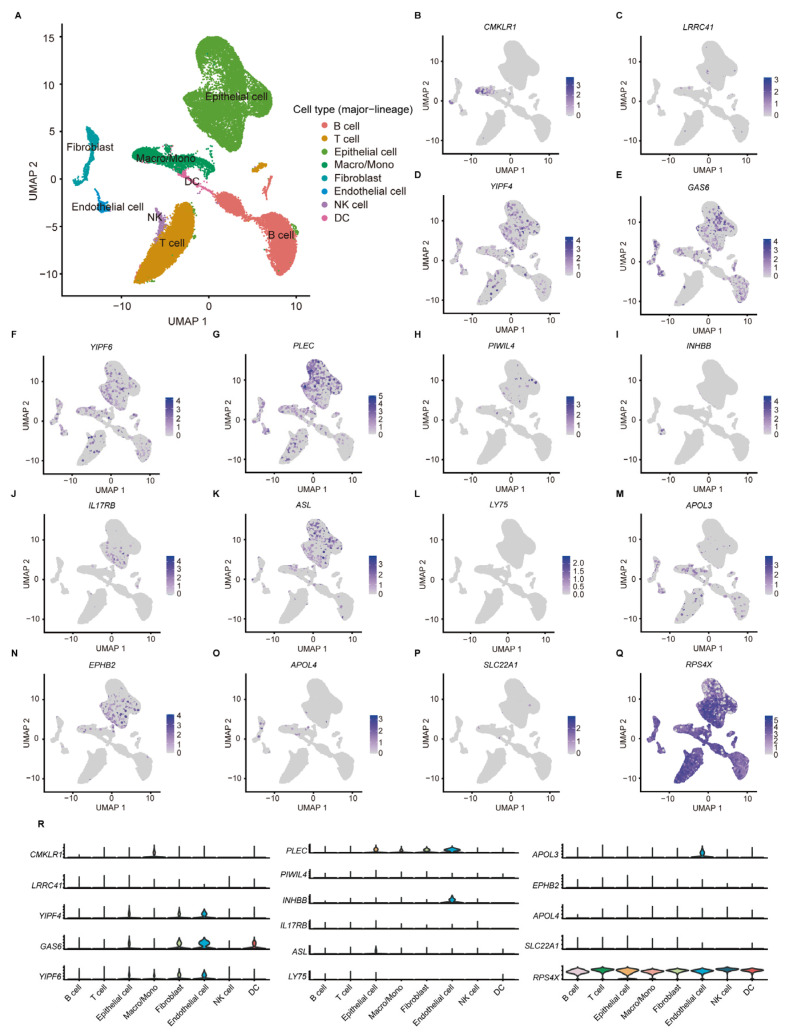
ScRNA-seq analysis of aDCRGs based on 11 tumor tissues of 10 CC patients in GSE166555. (**A**) Annotation of all cell categories in GSE166555. (**B**–**Q**) Expression of each aDCRG in all cell categories. (**R**) Violin diagrams exhibiting the expression of aDCRGs in each cell type. Different colors represent different cell types. Macro/Mono: Macrophage/Monocyte.

According to the scRNA-seq data from 11 tumor tissues of 10 CC patients in GSE166555, the TME of CC can be classified into eight cell subtypes (Figure 6A). Among the 17 aDCRGs, the expression of *RPS4X* is ubiquitous in all cell populations, while *GAS6* and *RPS4X* exhibit relatively high expression in DCs (Figure 6B–R). The expression pattern of *C16orf78* is not available in the dataset. The results of cell clustering and annotation in detail are demonstrated in Figure S4.

### 2.7. Establishment and Verification of a Prognostic Nomogram Constituted by the aDCRS and Clinicopathological Characteristics

**Figure 7:**
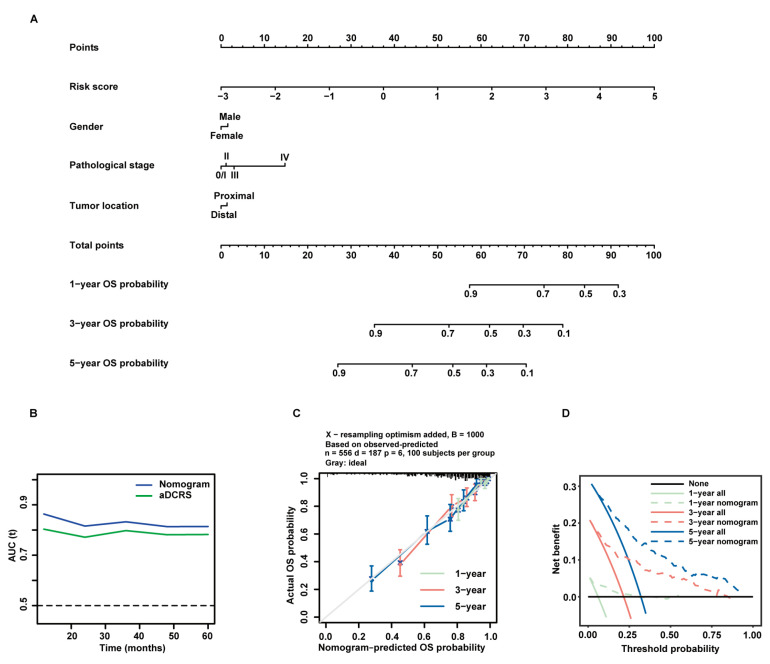
Formulation and validation of a prognostic nomogram incorporating the aDCRS and clinicopathological features based on the training set. (**A**) Nomogram for predicting 1-year, 3-year, and 5-year OS rates. (**B**) tROC curves exhibit that the nomogram had better discriminatory ability than the aDCRS. (**C**) Calibration plots demonstrate a strong pertinence between the actual (*y*-axis) and predicted probabilities (*x*-axis) of 1-year, 3-year, and 5-year OS. The grey line denotes the ideal fit. The green, red, and blue lines represent nomogram prediction, of which a closer fit to the grey line indicates better performance. Circles signify nomogram-predicted probabilities, whereas crosses reflect the bootstrapping-corrected estimates. Error bars stand for the 95% CIs of these estimates. (**D**) DCA of the nomogram at 1-year, 3-year, and 5-year follow-up.

In an attempt to improve the predictive performance of the aDCRS, we investigated the prognostic value of three clinicopathological variables including gender, pathological stage at diagnosis, and tumor location based on patients in the training set. The baseline characteristics of patients in the alive and dead groups are demonstrated in Table 1. Univariate Cox-PH regression analysis elucidated that the risk score and pathological stage were independent predictors for OS probabilities (Table 2). Considering that the selection of predictive factors can rely on both statistical significance and clinical importance, we integrated all of the three clinicopathological variables with the risk score and constructed a nomogram using multivariate Cox-PH regression (Figure 7A). The results of multivariate Cox-PH regression analysis are shown in Table 3.

The tROC curves of the nomogram model and the aDCRS based on the training set were delineated for OS status (Figure 7B). The AIC, C-index, and bootstrapping-corrected C-index values of the nomogram were calculated as 1983.558, 0.781, and 0.774, respectively, and the 1-year, 3-year, and 5-year AUC values of the nomogram were computed as 0.863, 0.833, and 0.814, respectively, indicating that the nomogram is superior to the aDCRS in terms of model fit and discriminative power. Furthermore, calibration analysis verified the reliability of the predicted 1-, 3-, and 5-year OS probabilities (Figure 7C). The decision curves analysis (DCA) further confirmed that the nomogram had prominent clinical application value at 1-, 3-, and 5-year follow-up (Figure 7D).

## 3. Discussion

With the advent of the era of precision medicine, novel prognostic algorithms with enhanced predictive accuracy and clinical usefulness are urgently warranted to identify patients most likely to benefit from specific therapies and thereby improve the long-term outcomes of CRC patients.

Given that the reciprocal interplay between tumor cells and surrounding immune cells markedly impacts all stages of tumorigenesis [22], investigation of the activation and infiltration profiles of immune cells in the TME niche may add a new dimension to current understanding. It is noteworthy that immune responses mediated by T helper 1 cells in CRC tumors signify a favorable outcome, while immune responses mediated by T helper 17 cells denote a poor prognosis [23]. Activated tumor-infiltrating B cells were significantly subdued in CRC with liver metastasis whereas these cells thrived in non-metastatic tumors, and their infiltration in primary tumors has proven to repress the liver metastasis of CRC in animal models [24]. Moreover, the presence of tumor-infiltrating natural killer (NK) cells generally forecasts favorable clinical outcomes in CRC patients [25], and attenuated preoperative NK cell activity correlates with lower postoperative OS rates and lesser cumulative metastasis-free rates in curatively operated stage I–III CRC [26].

Considering that the infiltration abundance of immune cells plays a decisive role in the long-term outcomes of CRC patients, multinational efforts have been made to develop a novel immune-related methodology designated “Immunoscore”, which is highly recommended as an alternative for the TNM system for prognostic classification of CRC patients. The “Immunoscore” is based on immune densities of two lymphocyte populations (CD3+ and CD8+ T cells) that infiltrate the core of the tumor (CT) and invasive margin (IM) as quantified by immunohistochemistry (IHC). It has been revealed to be positively correlated with OS, DFS, and disease-specific survival while inversely correlated with relapse incidence [5,27]. Nevertheless, the intrinsic complexity and protocol variability of IHC inevitably compromise the stability of the results obtained. Additionally, appraisal of anti-tumor immunity via the “Immunoscore” is improper in biopsies, since an accurate delineation of the investigated tumor regions (CT and IM) is often no longer feasible [5].

The intricate TME in the CRC niche necessitates multifaceted investigation of the tumoricidal immunity. DCs are a heterogeneous leukocyte population composed of distinct subsets that perform diverse and almost contradictory functions including antigen presentation, T cell activation and differentiation, and immune tolerance [13,28]. Based on ontogeny, DCs can be basically categorized into conventional DCs (cDCs) and plasmacytoid DCs (pDCs), which exert distinct influence on T cell polarization [14,16]. pDCs are apt at eliciting regulatory T cell generation and thereby leading to immune tolerance and immunosuppression [28,29], while type 1 cDCs are the primary subtype that induce CD8+ T cell-mediated cytotoxicity via antigen cross-presentation [30]. Apart from their mission in adaptive immunity, DCs play an indispensable role in innate immune responses. Under the stimuli of microbial insult, DCs can produce large amounts of cytokines that participate in host defense, including IL-12 and IFNs. DCs can also activate NK cells and NKT cells, innate lymphocyte populations that swiftly kill selected targets and create a milieu rich in IL-4, IL-12, and IFN-γ cytokines [13,16].

Considering the multifunctional role that DCs play in innate and adaptive immunity, it is informative to focus on the prognostic ability of DC-related genes and propose a DC-oriented scoring mechanism to promote the survival stratification of CC patients. In the present study, aDC infiltration was identified and validated as the most robust protective factor for CC prognosis among 22 immune cell types via the CIBERSORT algorithm, univariate Cox-PH regression, and KM analysis. GSEA further revealed that the poor survival outcomes of high-risk CC patients may be partially ascribable to inhibition of DC maturation, chemotaxis, and migration. Previous experimental data exposited that the suppression of DC maturation can be linked to an increased risk of death in CC patients [31], further consolidating our findings.

A total of 17 aDCRGs that are significantly associated with OS status of CC patients were screened out and incorporated into our prognostic gene signature. The previous literature has explored the pertinence between some of these genes and tumor progression and patient survival in CRC. *CMKLR1* is a G-protein coupled receptor that is abundantly expressed in immature DCs [32]. Recent studies expounded that *CMKLR1* expression was positively correlated with the tumor size of CRC patients, suggesting that *CMKLR1* activity may facilitate tumor progression in CRC [33]. The underlying rationales may be that *CMKLR1* is up-regulated in tumors with low vascularity and low budding in response to a hypoxic microenvironment and participates in the regulation of TME [34]. As a γ-carboxyglutamic acid domain-containing protein that can engender platelet-mediated thrombosis, *GAS6* has been implicated to promote cancer cell proliferation in intestinal cancer cell lines [35]. In human CRC specimens, *GAS6* overexpression is detected in more than 70% of CRC by IHC, and is positively correlated with less differentiated histological grading, advanced lymph nodes status, and tumor stage [36]. Furthermore, the expression levels of *PIWIL4* were significantly higher in cancer than in adjacent mucosa, while *EPHB2* expression was higher in adjacent normal tissues than in tumorous tissues. Both overexpression of *PIWIL4* and attenuated expression of *EPHB2* might promote distant metastasis in CRC [37,38]. In our study, multivariate Cox-PH regression analysis identified *CMKLR1*, *GAS6*, and *PIWIL4* as unfavorable factors while treating *EPHB2* as a protective factor for CC prognosis, which is concordant with the aforementioned findings.

Moreover, Huang et al., reported that higher expression of *ASL* was detected in CC tissues in contrast to adjacent normal tissues, and patients with elevated *ASL* levels culminated in poorer survival outcomes [39]. Goto et al., ascertained *IL17RB* as a cancer stem cell (CSC)-specific cell surface marker in human CRC and confirmed that continuous ablation of *IL17RB*-expressing CSCs robustly subdued tumor growth in vivo [40]. Nonetheless, our results of multivariate analysis propose divergent trends compared with previous studies by exhibiting that *ASL* and *IL17RB* were protective factors for the OS status of CC patients. Further experimental and clinical examination are required to clarify the impacts of these genes on CC prognosis. Additionally, the prognostic significance of *C16orf78*, *LRRC41*, *YIPF4*, *YIPF6*, *PLEC*, *INHBB*, *LY75*, *APOL3*, *APOL4*, *SLC22A1* and *RPS4X* in CC remain to be illuminated in subsequent studies.

Previous studies have underscored the pertinence between some of the aDCRGs and anticancer drug response. The *GAS6/AXL* signaling pathway has been preclinically and clinically investigated as a therapeutic target for chemotherapy and immunotherapy in various cancer types, since the combination of *AXL* inhibitors with chemotherapeutic or immunotherapeutic agents can restore sensitivity and overcome therapeutic resistant tumors [41,42,43]. Additionally, in patients with hepatocellular carcinoma, the overexpression of *ASL* may give rise to drug resistance against arginine deprivation therapy [44], while targeting a positive feedback loop involving *EPHB2* may be a promising therapeutic strategy to combat cancer stemness and sorafenib resistance [45]. However, the role of other aDCRGs in anticancer drug response is still far from explicit.

Therefore, we deployed multifaceted approaches including the “oncoPredict” package, CTR-DB, and TIDE analysis for assessing the utility of the aDCRS in predicting chemotherapy and immunotherapy response. The “oncoPredict” package disclosed significant correlation between the risk score and estimated sensitivity scores of multitudinous anticancer drugs. Among them, 5-FU was one of the first chemotherapeutics reported to have anticancer function in different malignancies [46]. Owing to the unsatisfactory therapeutic effectiveness and apparent side effects when 5-FU was administered alone, a combination of 5-FU and its chemoprotectant termed leucovorin (LV) has occupied the mainstream status in systemic treatment of metastatic CRC for a long time [46,47]. Since irinotecan, a semisynthetic derivative of camptothecin, and oxaliplatin, a diaminocyclohexane platinum complex were introduced, clinicians have adopted the FOLFIRI (5-FU/LV plus irinotecan) and FOLFOX (5-FU/LV plus oxaliplatin) as the standard first-line chemotherapy strategy for metastatic CRC [46,48,49], which have proven to extend overall survival of CRC patients by approximately 2 years [50]. Our results imply that patients with higher risk scores were more resistant to these first- or second-line chemotherapeutic agents, thus authenticating the predictive value of our aDCRS in chemotherapy response.

Additionally, to appraise the potential efficacy of ICB treatment, the primary targets of which are programmed death 1, programmed death-ligand 1, and cytotoxic T lymphocyte-associated protein 4 [51], we conducted TIDE analysis and compared the risk scores between supposed responders and non-responders to ICB therapy. Given that publicly available gene expression profiles and survival data of CC patients receiving ICB treatment are currently scarce, we cannot find real response information to draw more convincing conclusions, and further evaluations are warranted to corroborate the ability of our aDCRS in predicting ICB response.

In an attempt to improve the predictive performance of the aDCRS, we incorporated the risk score with clinicopathological variables including gender, pathological stage, and tumor location to formulate a prognostic nomogram based on patients in the training set. Pathological stage at diagnosis is considered the most crucial predictor of survival, with a 5-year survival rate ranging from 91% for localized lesions to 14% for distant diseases [52]. As for anatomic site, proximal CC tends to yield significantly worse 5-year survival and higher mortality rates when compared to distal CC [53]. Gender disparities in overall death and 5-year survival rates are also pronounced in CC patients. Although the incidence of proximal CC is currently lower in men (35%) than in women (44%), indicating more unfavorable tumor locations in women, the mortality rate of women (11 per 100,000) is still lower than that in men (15.7 per 100,000) [52]. Such divergence can be boiled down to both endogenous and environmental factors, including the protective effect of estrogen and tumor molecular traits that presumably impact treatment response [54,55].

In comparison with previous research, the major strength of our study is that the aDCRS-based nomogram exhibited exceptional discriminatory ability since the AUC values for 1-, 3-, and 5-year OS rates were higher than 0.8. Additionally, the calibration plots illuminate that the nomogram-predicted probabilities for 1-, 3-, and 5-year OS are closely consilient with the actual probabilities, and the DCA curves illustrate that our model has abundant net benefit for clinical utilization.

There remain several limitations that should be underscored when interpreting our results. Firstly, the RNA sequencing (RNA-seq) and microarray profiles of 1224 CC patient samples enrolled in this retrospective study were obtained from publicly available datasets only instead of prospective clinical trials, so the prognostic robustness and clinical usefulness of our aDCRS and nomogram should be further validated. Secondly, more detailed basic experiments (both in vitro and in vivo) are warranted to verify the mechanistic underpinning of the observed association between aDC infiltration and patient survival. Lastly, some vital clinicopathological parameters, such as age, T stage, N stage, and M stage were not investigated in this study owing to missing values concerning these parameters of studied patients in the dataset. We will further refine our nomogram model using patient information from other datasets in the future.

## 4. Materials and Methods

### 4.1. Data Sources and Preprocessing

The microarray datasets GSE39582, GSE17536, and GSE17537 were downloaded from the Gene Expression Omnibus (GEO) public database (http://www.ncbi.nlm.nih.gov/geo/, accessed on 2 November 2022). The annotation of gene symbols was anchored in the corresponding probe in the GPL570 platform (Affymetrix Human Genome U133 Plus 2.0 Array). We utilized the “normalize Between Arrays” function in the “limma” R package for normalization of the expression values so that they have a similar distribution in a group of arrays. Moreover, transcriptome RNA-seq profiles and clinical information from The Cancer Genome Atlas (TCGA, https://portal.gdc.cancer.gov, accessed on 8 November 2022)-colon adenocarcinoma (COAD) dataset were downloaded as transcripts per kilobase million values and log2 transformed.

To obtain reliable results from downstream analysis, we excluded samples with missing expression profiles or follow-up information in GEO and TCGA datasets and eventually enrolled 1224 CC patients in this study. Among them, 562 eligible patients from GSE39582 were employed as the training set. Furthermore, 177 CC patients from GSE17536 and 55 CC patients from GSE17537 were integrated as the first independent validation set. Additionally, 430 eligible CC patients from TCGA-COAD were employed as the second independent validation set.

### 4.2. Immune Infiltration Analysis and Assessment of the Prognostic Value of Immune Cell Types

We utilized the “CIBERSORT” R script [56] with the leukocyte signature matrix and 1000 permutations to quantify the infiltration abundance of 22 immune cell types in each tumor sample of patients from all datasets (562 from GSE39582, 177 from GSE17536, 55 from GSE17537, 430 from TCGA-COAD). The results were visualized via heatmaps using the “pheatmap” package. Based on the CIBERSORT scores and OS status of 562 CC patients in the training set, univariate Cox-PH regression was performed using the “survival” package to delve into the pertinence between the infiltration degree of distinct immune cell types and survival outcomes. To consolidate the prognostic value of specific immune cell type, 562 patients were assigned to the high- and low-infiltration groups according to the optimal cutoff value of CIBERSORT scores of each immune cell type using the “surv_cutpoint” function of the “survminer” package. KM analysis with log-rank statistical tests was conducted to compare the OS probabilities between the two groups.

### 4.3. Screening of Hub Genes and Formulation of the aDCRS

Given that aDC was identified as the protective immune cell type with the strongest pertinence, differential gene expression analysis was performed between the high- and low-aDC-infiltration groups using the empirical Bayesian method from the “limma” package [57]. The DEGs significantly correlated with aDC infiltration degree were filtrated with the threshold of *p*-value < 0.01. Based on 556 patients with a follow-up period more than one month in the training set, univariate Cox-PH regression analysis was utilized to select the most valuable prognostic genes among the aDC-related DEGs with the criteria of *p*-value < 0.05. In order to avoid redundancy or overfitting, we then employed LASSO regression, which is a linear regression that imposes shrinkage penalty on the magnitude of the model coefficients using the 10-fold cross-validation approach [58], to identify the genes with individual nonzero coefficients. Subsequently, we adopted the stepwise multivariate Cox-PH regression anchored in the AIC by setting “sls” and “sle” as 0.05. DEGs associated with the OS of CC patients were incorporated into a prognostic gene signature called “aDCRS”. The signature was established by integrating normalized gene expression values weighted by their β coefficients in Cox-PH analysis according to the following formula:

risk score=Coef(X1)×Exp(X1)+…+Coef(Xn)×Exp(Xn),

where Coef(Xn) and Exp(Xn) represent the coefficient and mRNA expression of certain hub gene. Furthermore, the mRNA levels of each aDCRG in tumor tissues and adjacent normal tissues were compared using Student’s *t*-test or the Wilcoxon–Mann–Whitney test based on the training set and the second validation set.

### 4.4. Examination of the Model Fit, Discriminatory Power, and Risk-Stratification Competence of the aDCRS in Predicting CC Prognosis

Performance of the aDCRS was preliminarily appraised via the AIC, C-index, and AUC values. Anchored in the concept of entropy, AIC is a well-documented criterion for measuring the goodness of fit of regression models. A lower AIC value signifies a better model fit [59]. In tandem, the discriminatory ability of the aDCRS was measured as the C-index and bootstrapping-corrected C-index as well as the 1-year, 3-year, and 5-year AUC values using the “survival” package and depicted as tROC curves using the “timeROC” package. To evaluate the risk-stratification capacity of the aDCRS, we divided patients into the high- and low-risk groups according to the optimal cutoff value of risk scores using the “surv_cutpoint” function and utilized the KM method and log-rank tests to assess whether the OS probabilities were significantly different between the two groups. Moreover, we further divided CC patients into the alive group and the dead group and compared their risk scores using Student’s *t*-test or the Wilcoxon–Mann–Whitney test.

### 4.5. Verification of the Pertinence between the aDCRGs and DC Activation and Infiltration and Functional Enrichment Analysis

The CIBERSORT aDC scores in the high- and low-risk groups were compared using the Wilcoxon–Mann–Whitney test based on the training set. The Pearson correlation test was implemented between individual aDCRG and CIBERSORT aDC scores based on the training set, as well as between individual aDCRG and aDC/DC markers based on the training set and two validation sets.

Additionally, we downloaded the hallmark gene sets and DC-relevant gene sets from the Molecular Signatures Database (https://www.gsea-msigdb.org/gsea/msigdb/index.jsp, accessed on 27 January 2023) and performed GSEA (https://www.gsea-msigdb.org/gsea, accessed on 27 January 2023) with the “clusterProfile” package [60]. All transcripts were ranked by the log_2_ (fold change). Each run was conducted with 1000 permutations and a statistical threshold of *p* < 0.05.

### 4.6. Investigation of the Practicability of the aDCRS in Predicting Chemotherapy and Immunotherapy Response

Based on the experimentally screened IC50 values of anticancer drugs in various intestinal tissue cell lines obtained from the Genomics of Drug Sensitivity in Cancer (GDSC, http://www.cancerrxgene.org, accessed on 3 February 2023) 2 database, we deployed the “calcPhenotype” function of the “oncoPredict” package [61] to calculate the imputed sensitivity scores of 562 patients in the training set and further analyzed the correlation between the risk score and drug response imputations using Pearson correlation analysis. We also picked out six chemotherapeutic drugs and compared their predicted therapeutic effects between the high-risk and low-risk patients using Student’s *t*-test or the Wilcoxon–Mann–Whitney test. To further interrogate the practicability of the aDCRS in predicting chemotherapy response in CC patients, we classified 67 CC patients in the TCGA-COAD dataset, the data for whom were collected and curated using the CTR-DB (http://ctrdb.cloudna.cn, accessed on 15 February 2023) into the response and non-response groups, and compared their risk scores using Student’s *t*-test.

Furthermore, TIDE (http://tide.dfci.harvard.edu**,** accessed on 26 February 2023) analysis was carried out to determine the performance of the aDCRS in distinguishing ICB response in CC patients. A higher TIDE score signifies an increased risk of tumor immune escape and a lowered likelihood of gaining benefit from ICB therapy [51]. According to the “responder” information, patients in the training set and two validation sets were categorized into the responder and non-responder groups, respectively, with a threshold of 0 and compared using Student’s *t*-test or the Wilcoxon–Mann–Whitney test.

### 4.7. Processing of ScRNA-Seq Data and Cell Type Annotations

We employed the “Seurat” package [62] in R version 4.2.2 (The R Foundation, Vienna, Austria) to process and visualize the scRNA expression profiles of CC patients in dataset GSE166555 downloaded from GEO. After strict procedures of quality control as reported by Uhlitz et al. [63], the GSE166555 dataset filtered 34,897 cells from 11 tumor tissues of 10 CC patients. After normalizing the cells with the “NormalizeData” function, we then normalized the gene expression matrix to the total unique molecular identifier counts per cell and converted them to the natural log scale using the “ScaleData” function. Anchored in the highly variable genes, we carried out principle component analysis to detect significantly available dimensions, termed principle components (PCs) [64]. Afterwards, the uniform manifold approximation and projection algorithm were applied to reduce dimensionality with 20 initial PCs and to conducting cluster classification across all cells. According to a cutoff threshold of |log_2_ [fold change]| > 0.25, we utilized the “FindAllMarkers” function to select the marker genes of each cell cluster. Subsequently, different cell clusters were annotated automatically with the “singleR” package in conformity with the composition patterns of the marker genes [65]. We then manually scrutinized and corrected the annotations using classical marker genes provided by the CellMarker 2.0 database [66].

### 4.8. Establishment and Validation of a Prognostic Nomogram Incorporating the aDCRS and Clinicopathological Features

In total, 556 CC patients in the training set were categorized into the alive group and the dead group according to their OS status. Three clinicopathological variables that are well-recognized factors for CRC mortality anchored in the previous literature [52,53] and recorded with complete information in 556 patients were chosen as candidate predictors, including gender, pathological stage at diagnosis, and tumor location. Proximal or distal colon cancers were divided at the splenic flexure, and patients were staged according to the AJCC/UICC TNM classification system. Univariate Cox-PH regression analysis was conducted to probe their association with OS probabilities. Considering their clinical importance, we incorporated all of these variables with the risk score to build a prognostic nomogram using multivariate Cox-PH regression.

The goodness of fit of the nomogram model was appraised via the AIC. The discriminatory ability was measured as the C-index and bootstrapping-corrected C-index as well as the 1-year, 3-year, and 5-year AUC values using the “survival” package and depicted as the tROC curves using the “timeROC” package. Moreover, the calibration of the nomogram was assessed by comparing the predicted and actual probabilities of 1-, 3-, and 5-year survival outcomes. Bootstraps with 1000 resamples were employed for these activities. Moreover, the clinical utility of the nomogram was judged via DCA at 1-, 3-, and 5-year follow up.

### 4.9. Bioinformatic and Statistical Analysis

The Shapiro–Wilk test was utilized to examine the distribution of continuous variables. Continuous variables with normal distribution were compared using Student’s *t*-test. Continuous variables with non-normal distribution were compared using the Wilcoxon–Mann–Whitney test. Categorical variables were compared using the Pearson Chi-square test or continuity correction Chi-square test. IBM SPSS Statistics 20 (IBM Corp., Armonk, NY, USA) and R software (version 4.0.3, http://www.r-project.org, accessed on 24 April 2022) were used to analyze data and plot graphs, unless otherwise specified. A *p*-value < 0.05 was considered statistically significant.

## 5. Conclusions

To summarize, based on transcriptome profiles and clinical data of CC samples from the GEO and TCGA databases, we went beyond conventional risk scoring models targeting tumor cells alone and established an aDC-oriented signature constituting 17 DEGs that were significantly correlated with both aDC infiltration abundance and OS probabilities to predict the survival outcomes and response to chemotherapy and immunotherapy of CC patients. Furthermore, we integrated the signature with clinicopathological features to derive a prognostic nomogram, which was corroborated to have enhanced predictive accuracy, prominent discriminative ability, and outstanding clinical utility. The aDCRS serves as a powerful analytical tool that incorporates putative genomic biomarkers predictive of survival outcomes and anticancer drug response of CC patients and will facilitate the development of novel cancer therapeutic strategies in the future.

## Figures and Tables

**Table 1:** Baseline clinicopathological characteristics of 556 patients in the training set.

Variables	Alive	Dead	*p*-Value
	(*n* = 369)	(*n* = 187)	
Risk score (median (IQRs))	−0.366 (−0.947, 0.196)	0.679 (−0.010, 1.352)	<0.0001
Gender (*n* (%))			0.079
Female	175 (47.425)	74 (39.572)	
Male	194 (52.575)	113 (60.428)	
Pathological stage (*n* (%))			<0.0001
0/I	30 (8.130)	6 (3.209)	
II	183 (49.593)	75 (40.107)	
III	136 (36.856)	67 (35.829)	
IV	20 (5.420)	39 (20.856)	
Tumor location (*n* (%))			0.904
Distal	223 (60.434)	114 (60.963)	
Proximal	146 (39.566)	73 (39.037)	

Continuous variables were compared using the Wilcoxon–Mann–Whitney test. Categorical variables including gender and tumor location were compared using the Pearson Chi-square test. The categorical variable pathological stage was compared using the continuity correction Chi-square test. All tests were two-sided. IQR: interquartile range.

**Table 2:** Univariate Cox-PH regression analysis of candidate variables for predicting CC prognosis based on the training set.

Variables	HR (95% CI)	*p*-Value
Risk score	2.718 (2.346–3.150)	<0.0001
Gender		0.061
Female	Reference	
Male	1.323 (0.987–1.774)	
Pathological stage		
0/I	Reference	
II	1.490 (0.648–3.426)	0.347
III	1.912 (0.829–4.409)	0.128
IV	7.760 (3.276–18.382)	<0.0001
Tumor location		0.587
Distal	Reference	
Proximal	1.085 (0.808–1.457)	

HR: hazard ratio; CI: confidence interval.

**Table 3:** Multivariate Cox-PH regression analysis of predictive factors constituting the nomogram based on the training set.

Variables	HR (95% CI)	*p*-Value
Risk score	2.428 (2.079–2.835)	<0.0001
Gender		0.468
Female	Reference	
Male	1.117 (0.829–1.504)	
Pathological stage		
0/I	Reference	
II	1.086 (0.471–2.500)	0.847
III	1.237 (0.534–2.865)	0.620
IV	2.848 (1.176–6.898)	0.020
Tumor location		0.520
Distal	Reference	
Proximal	3.369 (2.228–5.093)	

HRs were adjusted for risk score, gender, pathological stage, and tumor location.

## Data Availability

Publicly available datasets were analyzed in this study. These data can be found here: https://www.ncbi.nlm.nih.gov/geo/query/acc.cgi?acc=GSE39582, accessed on 2 November 2022; https://www.ncbi.nlm.nih.gov/geo/query/acc.cgi?acc=GSE17536, accessed on 2 November 2022; https://www.ncbi.nlm.nih.gov/geo/query/acc.cgi?acc=GSE17537, accessed on 2 November 2022; https://portal.gdc.cancer.gov/projects/TCGA-COAD, accessed on 8 November 2022.

## Supplementary Materials

The following supporting information can be downloaded at: https://www.mdpi.com/article/10.3390/ijms242115959/s1.

## Abbreviations

CC	colon cancer
aDC	activated dendritic cell
aDCRG	aDC-related prognostic gene
aDCRS	aDC-related gene signature
PH	proportional hazard
DEGs	differentially expressed genes
ROC	receiver operating characteristic
KM	Kaplan–Meier
CRC	colorectal cancer
AJCC/UICC	American Joint Committee on Cancer/Union Internationale Contre le Cancer
TME	tumor microenvironment
OS	overall survival
DFS	disease-free survival
CIBERSORT	Cell-type Identification by Estimating Relative Subsets of RNA Transcripts
LASSO	least absolute shrinkage and selection operator
AIC	Akaike information criterion
C-index	concordance index
AUC	area under the curve
GSEA	Gene Set Enrichment Analysis
CTR-DB	Cancer Treatment Response gene signature DataBase
TIDE	Tumor Immune Dysfunction and Exclusion
ICB	Immune checkpoint blockade
ScRNA-seq	Single-Cell RNA Sequencing
DCA	decision curves analysis
IFN-γ	interferon-γ
NK	natural killer
CT	the core of the tumor
IM	invasive margin
IHC	immunohistochemistry
cDCs	conventional DCs
pDCs	plasmacytoid DCs
CSC	cancer stem cell
LV	leucovorin
RNA-seq	RNA sequencing
GEO	Gene Expression Omnibus
PCs	principle components
IQRs	interquartile ranges

## References

[1] Sung H., Ferlay J., Siegel R.L., Laversanne M., Soerjomataram I., Jemal A., Bray F. (2021). Global Cancer Statistics 2020: GLOBOCAN Estimates of Incidence and Mortality Worldwide for 36 Cancers in 185 Countries. CA Cancer J. Clin..

[2] Ciardiello F., Ciardiello D., Martini G., Napolitano S., Tabernero J., Cervantes A. (2022). Clinical management of metastatic colorectal cancer in the era of precision medicine. CA Cancer J. Clin..

[3] Vogel J.D., Felder S.I., Bhama A.R., Hawkins A.T., Langenfeld S.J., Shaffer V.O., Thorsen A.J., Weiser M.R., Chang G.J., Lightner A.L. (2021). The American Society of Colon and Rectal Surgeons Clinical Practice Guidelines for the Management of Colon Cancer. Dis. Colon Rectum.

[4] Argilés G., Tabernero J., Labianca R., Hochhauser D., Salazar R., Iveson T., Laurent-Puig P., Quirke P., Yoshino T., Taieb J. (2020). Localised colon cancer: ESMO Clinical Practice Guidelines for diagnosis, treatment and follow-up. Ann. Oncol..

[5] Galon J., Mlecnik B., Bindea G., Angell H.K., Berger A., Lagorce C., Lugli A., Zlobec I., Hartmann A., Bifulco C. (2014). Towards the introduction of the ‘Immunoscore’ in the classification of malignant tumours. J. Pathol..

[6] Angell H.K., Bruni D., Barrett J.C., Herbst R., Galon J. (2020). The Immunoscore: Colon Cancer and beyond. Clin. Cancer Res..

[7] Bruni D., Angell H.K., Galon J. (2020). The immune contexture and Immunoscore in cancer prognosis and therapeutic efficacy. Nat. Rev. Cancer.

[8] Hanahan D., Weinberg R.A. (2011). Hallmarks of cancer: The next generation. Cell.

[9] Chen F., Zhuang X., Lin L., Yu P., Wang Y., Shi Y., Hu G., Sun Y. (2015). New horizons in tumor microenvironment biology: Challenges and opportunities. BMC Med..

[10] Dzobo K., Senthebane D.A., Dandara C. (2023). The Tumor Microenvironment in Tumorigenesis and Therapy Resistance Revisited. Cancers.

[11] Junttila M.R., de Sauvage F.J. (2013). Influence of tumour micro-environment heterogeneity on therapeutic response. Nature.

[12] Schmitt M., Greten F.R. (2021). The inflammatory pathogenesis of colorectal cancer. Nat. Rev. Immunol..

[13] Mellman I., Steinman R.M. (2001). Dendritic cells: Specialized and regulated antigen processing machines. Cell.

[14] Rescigno M. (2010). Intestinal dendritic cells. Adv. Immunol..

[15] Wang Y., Xiang Y., Xin V.W., Wang X.W., Peng X.C., Liu X.Q., Wang D., Li N., Cheng J.T., Lyv Y.N. (2020). Dendritic cell biology and its role in tumor immunotherapy. J. Hematol. Oncol..

[16] Eisenbarth S.C. (2019). Dendritic cell subsets in T cell programming: Location dictates function. Nat. Rev. Immunol..

[17] Malietzis G., Lee G.H., Jenkins J.T., Bernardo D., Moorghen M., Knight S.C., Al-Hassi H.O. (2015). Prognostic Value of the Tumour-Infiltrating Dendritic Cells in Colorectal Cancer: A Systematic Review. Cell Commun. Adhes..

[18] Karthaus N., Torensma R., Tel J. (2012). Deciphering the message broadcast by tumor-infiltrating dendritic cells. Am. J. Pathol..

[19] Dadabayev A.R., Sandel M.H., Menon A.G., Morreau H., Melief C.J., Offringa R., van der Burg S.H., Janssen-van Rhijn C., Ensink N.G., Tollenaar R.A. (2004). Dendritic cells in colorectal cancer correlate with other tumor-infiltrating immune cells. Cancer Immunol. Immunother..

[20] Nakayama Y., Inoue Y., Minagawa N., Katsuki T., Nagashima N., Onitsuka K., Tsurudome Y., Sako T., Hirata K., Nagata N. (2003). Relationships between S-100 protein-positive cells and clinicopathological factors in patients with colorectal cancer. Anticancer Res..

[21] Benson A.B., Venook A.P., Al-Hawary M.M., Arain M.A., Chen Y.J., Ciombor K.K., Cohen S., Cooper H.S., Deming D., Farkas L. (2021). Colon Cancer, Version 2.2021, NCCN Clinical Practice Guidelines in Oncology. J. Natl. Compr. Cancer Netw..

[22] Greten F.R., Grivennikov S.I. (2019). Inflammation and Cancer: Triggers, Mechanisms, and Consequences. Immunity.

[23] Fridman W.H., Pagès F., Sautès-Fridman C., Galon J. (2012). The immune contexture in human tumours: Impact on clinical outcome. Nat. Rev. Cancer.

[24] Xu Y., Wei Z., Feng M., Zhu D., Mei S., Wu Z., Feng Q., Chang W., Ji M., Liu C. (2022). Tumor-infiltrated activated B cells suppress liver metastasis of colorectal cancers. Cell Rep..

[25] Pietropaolo G., Scarno G., Stabile H., Grimaldi A., Gismondi A., Santoni A., Sciumè G. (2021). NK cell and ILC heterogeneity in colorectal cancer. New perspectives from high dimensional data. Mol. Asp. Med..

[26] Kondo E., Koda K., Takiguchi N., Oda K., Seike K., Ishizuka M., Miyazaki M. (2003). Preoperative natural killer cell activity as a prognostic factor for distant metastasis following surgery for colon cancer. Dig. Surg..

[27] Pagès F., Mlecnik B., Marliot F., Bindea G., Ou F.S., Bifulco C., Lugli A., Zlobec I., Rau T.T., Berger M.D. (2018). International validation of the consensus Immunoscore for the classification of colon cancer: A prognostic and accuracy study. Lancet.

[28] Lee Y.S., Radford K.J. (2019). The role of dendritic cells in cancer. Int. Rev. Cell Mol. Biol..

[29] Fu C., Jiang A. (2018). Dendritic Cells and CD8 T Cell Immunity in Tumor Microenvironment. Front. Immunol..

[30] Hildner K., Edelson B.T., Purtha W.E., Diamond M., Matsushita H., Kohyama M., Calderon B., Schraml B.U., Unanue E.R., Diamond M.S. (2008). Batf3 deficiency reveals a critical role for CD8alpha+ dendritic cells in cytotoxic T cell immunity. Science.

[31] Michielsen A.J., Noonan S., Martin P., Tosetto M., Marry J., Biniecka M., Maguire A.A., Hyland J.M., Sheahan K.D., O’Donoghue D.P. (2012). Inhibition of dendritic cell maturation by the tumor microenvironment correlates with the survival of colorectal cancer patients following bevacizumab treatment. Mol. Cancer Ther..

[32] Wittamer V., Franssen J.D., Vulcano M., Mirjolet J.F., Le Poul E., Migeotte I., Brézillon S., Tyldesley R., Blanpain C., Detheux M. (2003). Specific recruitment of antigen-presenting cells by chemerin, a novel processed ligand from human inflammatory fluids. J. Exp. Med..

[33] Kiczmer P., Seńkowska A.P., Kula A., Dawidowicz M., Strzelczyk J.K., Zajdel E.N., Walkiewicz K., Waniczek D., Ostrowska Z., Świętochowska E. (2020). Assessment of CMKLR1 level in colorectal cancer and its correlation with angiogenic markers. Exp. Mol. Pathol..

[34] Kiczmer P., Mielcarska S., Chrabańska M., Dawidowicz M., Kula A., Rynkiewicz M., Seńkowska A.P., Waniczek D., Piecuch J., Jopek J. (2021). The Concentration of CMKLR1 Expression on Clinicopathological Parameters of Colorectal Cancer: A Preliminary Study. Medicina.

[35] Akitake-Kawano R., Seno H., Nakatsuji M., Kimura Y., Nakanishi Y., Yoshioka T., Kanda K., Kawada M., Kawada K., Sakai Y. (2013). Inhibitory role of Gas6 in intestinal tumorigenesis. Carcinogenesis.

[36] Martinelli E., Martini G., Cardone C., Troiani T., Liguori G., Vitagliano D., Napolitano S., Morgillo F., Rinaldi B., Melillo R.M. (2015). AXL is an oncotarget in human colorectal cancer. Oncotarget.

[37] Li L., Yu C., Gao H., Li Y. (2010). Argonaute proteins: Potential biomarkers for human colon cancer. BMC Cancer.

[38] Oshima T., Akaike M., Yoshihara K., Shiozawa M., Yamamoto N., Sato T., Akihito N., Nagano Y., Fujii S., Kunisaki C. (2008). Overexpression of EphA4 gene and reduced expression of EphB2 gene correlates with liver metastasis in colorectal cancer. Int. J. Oncol..

[39] Huang H.L., Chen W.C., Hsu H.P., Cho C.Y., Hung Y.H., Wang C.Y., Lai M.D. (2017). Silencing of argininosuccinate lyase inhibits colorectal cancer formation. Oncol. Rep..

[40] Goto N., Fukuda A., Yamaga Y., Yoshikawa T., Maruno T., Maekawa H., Inamoto S., Kawada K., Sakai Y., Miyoshi H. (2019). Lineage tracing and targeting of IL17RB^+^ tuft cell-like human colorectal cancer stem cells. Proc. Natl. Acad. Sci. USA.

[41] Tanaka M., Siemann D.W. (2021). Therapeutic Targeting of the Gas6/Axl Signaling Pathway in Cancer. Int. J. Mol. Sci..

[42] Lin J.Z., Wang Z.J., De W., Zheng M., Xu W.Z., Wu H.F., Armstrong A., Zhu J.G. (2017). Targeting AXL overcomes resistance to docetaxel therapy in advanced prostate cancer. Oncotarget.

[43] Goyette M.A., Elkholi I.E., Apcher C., Kuasne H., Rothlin C.V., Muller W.J., Richard D.E., Park M., Gratton J.P., Côté J.F. (2021). Targeting Axl favors an antitumorigenic microenvironment that enhances immunotherapy responses by decreasing Hif-1α levels. Proc. Natl. Acad. Sci. USA.

[44] Hung Y.H., Huang H.L., Chen W.C., Yen M.C., Cho C.Y., Weng T.Y., Wang C.Y., Chen Y.L., Chen L.T., Lai M.D. (2017). Argininosuccinate lyase interacts with cyclin A2 in cytoplasm and modulates growth of liver tumor cells. Oncol. Rep..

[45] Leung H.W., Leung C.O.N., Lau E.Y., Chung K.P.S., Mok E.H., Lei M.M.L., Leung R.W.H., Tong M., Keng V.W., Ma C. (2021). EPHB2 Activates β-Catenin to Enhance Cancer Stem Cell Properties and Drive Sorafenib Resistance in Hepatocellular Carcinoma. Cancer Res..

[46] Vodenkova S., Buchler T., Cervena K., Veskrnova V., Vodicka P., Vymetalkova V. (2020). 5-fluorouracil and other fluoropyrimidines in colorectal cancer: Past, present and future. Pharmacol. Ther..

[47] Thirion P., Michiels S., Pignon J.P., Buyse M., Braud A.C., Carlson R.W., O’Connell M., Sargent P., Piedbois P. (2004). Modulation of fluorouracil by leucovorin in patients with advanced colorectal cancer: An updated meta-analysis. J. Clin. Oncol..

[48] Mocellin S., Baretta Z., Roqué I.F.M., Solà I., Martin-Richard M., Hallum S., Bonfill Cosp X. (2017). Second-line systemic therapy for metastatic colorectal cancer. Cochrane Database Syst. Rev..

[49] Grothey A. (2006). Is there a third-line therapy for metastatic colorectal cancer?. Semin. Oncol..

[50] Gustavsson B., Carlsson G., Machover D., Petrelli N., Roth A., Schmoll H.J., Tveit K.M., Gibson F. (2015). A review of the evolution of systemic chemotherapy in the management of colorectal cancer. Clin. Color. Cancer.

[51] Jiang P., Gu S., Pan D., Fu J., Sahu A., Hu X., Li Z., Traugh N., Bu X., Li B. (2018). Signatures of T cell dysfunction and exclusion predict cancer immunotherapy response. Nat. Med..

[52] Siegel R.L., Wagle N.S., Cercek A., Smith R.A., Jemal A. (2023). Colorectal cancer statistics, 2023. CA Cancer J. Clin..

[53] Wong R. (2010). Proximal tumors are associated with greater mortality in colon cancer. J. Gen. Intern. Med..

[54] Koo J.H., Leong R.W. (2010). Sex differences in epidemiological, clinical and pathological characteristics of colorectal cancer. J. Gastroenterol. Hepatol..

[55] Tsai Y.J., Huang S.C., Lin H.H., Lin C.C., Lan Y.T., Wang H.S., Yang S.H., Jiang J.K., Chen W.S., Lin T.C. (2018). Differences in gene mutations according to gender among patients with colorectal cancer. World J. Surg. Oncol..

[56] Newman A.M., Liu C.L., Green M.R., Gentles A.J., Feng W., Xu Y., Hoang C.D., Diehn M., Alizadeh A.A. (2015). Robust enumeration of cell subsets from tissue expression profiles. Nat. Methods.

[57] Ritchie M.E., Phipson B., Wu D., Hu Y., Law C.W., Shi W., Smyth G.K. (2015). limma powers differential expression analyses for RNA-sequencing and microarray studies. Nucleic Acids Res..

[58] Tibshirani R. (1996). Regression Shrinkage and Selection Via the Lasso. J. R. Stat. Soc. Ser. B Methodol..

[59] Akaike H. (1974). A new look at the statistical model identification. IEEE Trans. Autom. Control.

[60] Yu G., Wang L.G., Han Y., He Q.Y. (2012). clusterProfiler: An R package for comparing biological themes among gene clusters. Omics.

[61] Maeser D., Gruener R.F., Huang R.S. (2021). oncoPredict: An R package for predicting in vivo or cancer patient drug response and biomarkers from cell line screening data. Brief. Bioinform..

[62] Satija R., Farrell J.A., Gennert D., Schier A.F., Regev A. (2015). Spatial reconstruction of single-cell gene expression data. Nat. Biotechnol..

[63] Uhlitz F., Bischoff P., Peidli S., Sieber A., Trinks A., Lüthen M., Obermayer B., Blanc E., Ruchiy Y., Sell T. (2021). Mitogen-activated protein kinase activity drives cell trajectories in colorectal cancer. EMBO Mol. Med..

[64] Lall S., Sinha D., Bandyopadhyay S., Sengupta D. (2018). Structure-Aware Principal Component Analysis for Single-Cell RNA-seq Data. J. Comput. Biol..

[65] Aran D., Looney A.P., Liu L., Wu E., Fong V., Hsu A., Chak S., Naikawadi R.P., Wolters P.J., Abate A.R. (2019). Reference-based analysis of lung single-cell sequencing reveals a transitional profibrotic macrophage. Nat. Immunol..

[66] Hu C., Li T., Xu Y., Zhang X., Li F., Bai J., Chen J., Jiang W., Yang K., Ou Q. (2023). CellMarker 2.0: An updated database of manually curated cell markers in human/mouse and web tools based on scRNA-seq data. Nucleic Acids Res..

