# Supplementary material for: An Activated Dendritic-Cell-Related Gene Signature Indicative of Disease Prognosis and Chemotherapy and Immunotherapy Response in Colon Cancer Patients
Source: Int J Mol Sci. 2023 Nov 3;24(21):15959. doi: 10.3390/ijms242115959 (PMC10647347; doi:10.3390/ijms242115959)
Supplement: Supplementary file 1 [file ijms-24-15959-s001.zip › ijms-2605913-supplementary.pdf]

Supplementary Material

An Activated Dendritic Cell-Related Gene Signature Indicative of Disease Prognosis and Chemotherapy and Immunotherapy Response in Colon Cancer Patients

Yiben Ouyang[1,†], Mingqian Yu[1,†], Tiange Liu[1], Mengying Suo[1], Jingyi Qiao[1], Liqiang Wang[1,*] and Na Li[1,*]

[1] School of Medicine, Nankai University, Tianjin 300071 China; ed1501260@163.com (Y.O.); mingqianyu2022@163.com (M.Y.); nimbus2000e@163.com (T.L.); 15866725760@163.com (M.S.); 18092289178@163.com (J.Q.)
† These authors contributed equally to this work.
* Correspondence: liqiangw301@163.com (L.W.) ; lina08@nankai.edu.cn (N.L.)

1 Supplementary Figures and Tables

1.1 Supplementary Figures

Input Samples	B cells naive	B cells memory	Plasma cells	T cells CD8	T cells CD4 naive	T cells CD4 memory resting	T cells CD4 memory activated	T cells follicular helper	T cells regulatory (Tregs)	T cells gamma delta	NK cells resting	NK cells activated	Monocytes	Macrophages M0	Macrophages M1	Macrophages M2	Dendritic cells resting	Dendritic cells activated	Mast cells resting	Mast cells activated	Eosinophils	Neutrophils	P-value	Pearson correlation
GSM972056	0.0422	0	0.1285	0.0634	0	0.016	0.0013	0.0769	0.0956	0	0.0963	0	0	0.0424	0.028	0.069	0.0433	0.0376	0	0.2257	0	0.0338	0.011	0.2155
GSM972055	0.0083	0	0.2305	0.1354	0	0.0909	0	0.0529	0.0126	0	0.0162	0.0351	0	0.0809	0.0734	0.1413	0.0826	0	0	0.0257	0	0.0144	0.001	0.3769
GSM972054	0.0327	0	0.1149	0	0.0079	0.0666	0	0.061	0.0102	0.0092	0.0695	0	0	0.2861	0.0664	0.1503	0	0.0018	0	0.1027	0	0.0207	0.001	0.3458
GSM972053	0	0.1262	0.1289	0.0571	0	0	0.0608	0.0865	0.0218	0	0.0837	0	0.0238	0.0753	0.1186	0.0289	0.0672	0.053	0	0.0683	0	0	0.019	0.1841
GSM972052	0.0496	0	0.3343	0	0.0844	0	0.0819	0.0556	0.0204	0	0.0645	0	0	0.0516	0.0643	0.0706	0.026	0.0274	0	0.0695	0	0	<0.0001	0.3829
GSM972051	0.017	0	0.0971	0	0	0.0753	0.0484	0.0267	0.0029	0	0.09	0	0	0.1698	0.0444	0.2346	0.021	0.0279	0	0.1293	0	0.0155	0.005	0.2792
GSM972050	0.0394	0	0.4242	0.0084	0.0181	0.1563	0	0.0401	0.0312	0	0.0403	0	0.0204	0.0112	0	0.0259	0.0097	0.0466	0	0.1123	0.0158	0	<0.0001	0.4819
GSM972049	0	0.0788	0.0858	0	0.0126	0.1829	0	0.0183	0.0037	0	0.1113	0	0	0.1499	0.0162	0.0837	0.0033	0.0374	0	0.2146	0	0.0014	0.049	0.1489
GSM972048	0	0	0.3068	0	0.0607	0.1488	0	0.0163	0	0	0.1109	0	0	0.0933	0.0129	0.0715	0.0505	0.0337	0	0.0946	0	0	0.012	0.21
GSM972047	0	0	0.1342	0	0.0841	0.1102	0	0.0077	0.0092	0	0.0888	0	0	0.1371	0.0141	0.137	0.1361	0.0188	0.0486	0.0741	0	0	0.007	0.2529
GSM972046	0.0314	0	0.1366	0.0216	0	0.1778	0.0248	0.0487	0.019	0.079	0.0086	0	0	0.1038	0.0604	0.2032	0.0286	0	0	0.0455	0	0.011	<0.0001	0.3783
GSM972045	0.1086	0	0.1077	0	0	0.1026	0	0.1041	0.0398	0	0.1293	0	0	0.0442	0.0034	0.079	0.0387	0.0464	0	0.1947	0.0016	0	0.092	0.1175
GSM972044	0	0.0313	0.0961	0.0361	0	0.049	0	0.0203	0.0637	0	0.143	0	0.0066	0.0682	0.0091	0.0887	0.0829	0.0281	0	0.2214	0	0.0555	0.092	0.1179
GSM972043	0	0.0718	0.0663	0	0.0199	0.1526	0.1478	0	0.0081	0	0.1055	0	0	0.1639	0.0247	0.0839	0.0358	0.0209	0	0.0743	0.0247	0	0.115	0.109
GSM972042	0	0.006	0.402	0.0038	0	0.1146	0	0.0093	0.0471	0.0106	0.0527	0	0	0.0742	0.0423	0.0895	0.0267	0.0599	0	0.0603	0	0.001	<0.0001	0.4486
GSM972041	0	0.0532	0.0596	0	0.0069	0.0233	0.0697	0.0656	0.019	0	0.0889	0	0	0.2005	0.0384	0.0686	0.0143	0.0445	0	0.2029	0	0.0446	0.025	0.1779
GSM972040	0	0.0028	0.1215	0	0.0181	0.1582	0.0557	0.0244	0	0	0.1313	0	0.0002	0.113	0.0184	0.1067	0.0734	0.0635	0	0.1127	0	0	0.183	0.085
GSM972039	0.0194	0	0.3936	0.0277	0	0.0643	0.0133	0.0513	0	0.0465	0.0245	0	0.0371	0.0174	0.0121	0.0627	0	0.034	0.0169	0.1555	0	0.0235	<0.0001	0.4911
GSM972038	0.0349	0	0.0934	0	0	0.0218	0.0529	0.0909	0.018	0.0604	0.072	0	0	0.0473	0.04	0.1355	0.0275	0.1499	0	0.1436	0.0076	0.0042	0.063	0.1354
GSM972037	0	0.0224	0.273	0	0.012	0.0866	0	0.0839	0	0	0.0772	0	0	0.0676	0.0425	0.0577	0.0158	0.0526	0	0.1892	0	0.0195	0.001	0.34
GSM972036	0.027	0	0.2053	0.061	0	0.1508	0.0722	0.0247	0.0227	0	0.1029	0	0	0.0749	0.0407	0.0935	0.0238	0.0018	0	0.0933	0	0.0054	0.007	0.2613
GSM972035	0	0.021	0.1324	0.0163	0	0.1499	0.0262	0.0146	0.0135	0	0.132	0	0.0001	0	0.016	0.1932	0.1173	0.0595	0	0.1079	0	0	0.337	0.0498
GSM972034	0	0.0523	0.2761	0.0169	0.0344	0.0373	0.0128	0	0.0672	0	0.0947	0	0	0.0241	0.0401	0.1031	0.0571	0.053	0	0.131	0	0	0.01	0.2264
GSM972033	0.0886	0	0.0889	0	0.0905	0	0.104	0.078	0.0686	0	0.1062	0	0	0.0463	0.0193	0.0123	0.0787	0	0	0.1912	0	0.0274	0.588	0.0151
GSM972032	0.0216	0.0025	0.1231	0	0.0818	0.217	0.073	0.0568	0	0.0066	0.0788	0	0	0.1036	0.0178	0.0693	0.0102	0.0309	0	0.1069	0	0	0.035	0.1638
GSM972031	0	0	0.209	0	0.0242	0.1008	0.0349	0.0492	0.0208	0	0.0772	0	0.0027	0.1781	0.048	0.1001	0.0283	0.0183	0	0.1084	0	0	0.009	0.2355
GSM972030	0	0.0059	0.2414	0.0335	0.0665	0.0261	0	0.0156	0.0086	0	0.0663	0	0.0197	0.1793	0.0397	0.1528	0.0077	0.0434	0	0.0768	0	0.017	<0.0001	0.4743
GSM972029	0.0475	0	0.1846	0.0577	0.0328	0.087	0.0373	0.0325	0	0	0.0978	0	0	0.172	0.0136	0.0888	0.0143	0	0.0169	0.1172	0	0	<0.0001	0.4223
GSM972028	0.0085	0	0.2878	0.0723	0.0143	0.0848	0	0.0392	0.0143	0	0.0849	0	0	0.001	0.0227	0.1171	0.047	0.0445	0	0.1111	0	0.0505	<0.0001	0.518
GSM972027	0	0.0987	0.131	0.0056	0.0626	0	0.0609	0.0377	0	0	0.0926	0	0	0.2194	0	0.1018	0.004	0.0511	0	0.1168	0	0.0177	0.068	0.131
GSM972026	0.0471	0	0.0999	0.0502	0.0527	0	0.1038	0.025	0	0	0.0588	0	0.0266	0	0.0445	0.2325	0.0852	0.0151	0	0.1584	0.0001	0	<0.0001	0.456
GSM972025	0	0	0.2931	0.0989	0	0.1686	0	0.0155	0.005	0	0.0427	0	0	0.0025	0.0257	0.1078	0.092	0.0149	0	0.1246	0.005	0.0038	0.001	0.3146
GSM972024	0	0.0188	0.0951	0.0217	0	0.1769	0	0.0429	0.071	0	0.1197	0	0	0.1269	0.0073	0.05	0.0419	0.032	0	0.1959	0	0	0.214	0.0756
GSM972023	0.0714	0	0.1094	0	0.0419	0.1018	0	0	0.026	0	0.0854	0	0	0.1843	0.0171	0.0644	0	0.0078	0	0.2811	0	0.0095	0.01	0.2316
GSM972022	0	0.024	0.2109	0.1138	0	0.1308	0	0.0644	0.0297	0	0.0028	0.0123	0	0.0744	0.0813	0.1569	0.0617	0.0017	0.0019	0.0258	0	0.0076	<0.0001	0.4636
GSM972021	0	0.0203	0.1063	0.013	0	0.1338	0	0.0976	0.0014	0	0.0673	0	0	0.1826	0.0696	0.1685	0.0206	0	0	0.1143	0	0.0047	0.007	0.262
GSM972020	0.0223	0	0.3144	0	0	0.1229	0.0398	0.0637	0	0	0.0628	0	0	0.0419	0.0602	0.0898	0.0563	0.0276	0.0464	0.0319	0.0156	0.0044	<0.0001	0.4286
GSM972019	0.0367	0	0.1984	0.0671	0	0.0704	0.0312	0.1481	0	0.0887	0	0	0	0.0583	0.0991	0.0867	0.0333	0.0145	0	0.0675	0	0	<0.0001	0.5598
GSM972018	0	0	0.1094	0	0	0.0323	0.0056	0.0995	0.0053	0	0	0.057	0	0.0866	0.2066	0.1507	0.0656	0.0031	0	0.1036	0.0029	0.0717	<0.0001	0.4464
GSM972017	0.032	0	0.1827	0.0391	0.0132	0.0115	0.0445	0.0231	0.0531	0.0171	0.0808	0	0	0.2549	0.026	0.1184	0	0	0	0.1036	0	0	<0.0001	0.4324
GSM972016	0.029	0	0.1453	0	0.0354	0.0586	0	0.0548	0.0082	0	0.0556	0	0	0.1886	0.0091	0.0888	0	0.0238	0	0.2755	0	0.0273	0.001	0.3392
GSM972015	0	0.0019	0.0341	0.0832	0	0.0172	0.0174	0.0441	0.0651	0	0.0794	0	0	0.1675	0.1297	0.1201	0.004	0.0371	0	0.1321	0	0.0671	0.045	0.1536
GSM972014	0	0.0044	0.0724	0.0232	0	0.0552	0	0.0422	0.0349	0	0.0624	0	0	0.0833	0.0425	0.1236	0.0362	0.0285	0	0.3237	0.0385	0.029	0.011	0.2162
GSM972013	0	0.0004	0.1786	0.0058	0.0137	0.105	0.0569	0.0178	0.0023	0	0.1237	0	0	0.0439	0.0252	0.1121	0.0044	0.0377	0	0.2036	0	0.0689	0.09	0.1196
GSM972012	0	0	0.2455	0	0.0082	0.2279	0	0.018	0	0	0.0714	0	0	0.1261	0.0224	0.089	0	0.05	0	0.1416	0	0	0.012	0.2098
GSM972011	0.0571	0	0.3067	0.0911	0	0.0192	0	0.0801	0	0.0401	0	0.012	0.0078	0.0393	0.1202	0.1219	0.0231	0	0.0046	0.0752	0.0015	0	<0.0001	0.5989
GSM972010	0.0287	0	0.1298	0.0475	0.0049	0.045	0.067	0.0243	0	0	0.0333	0	0	0.17	0.0304	0.1261	0	0.0355	0	0.2116	0.0192	0.0268	0.006	0.268
GSM972009	0.0306	0	0.3607	0.0623	0	0.0759	0	0.0722	0	0	0.0482	0	0	0.0094	0.0189	0.0723	0.0306	0.0345	0	0.1667	0.0101	0.0077	<0.0001	0.4954
GSM972008	0.0067	0	0.0833	0.0482	0.0221	0	0.0271	0.0141	0.0144	0	0.0406	0	0.0357	0.1081	0.0175	0.1936	0.071	0.0607	0	0.0541	0.0182	0.1844	<0.0001	0.4061
GSM972007	0.0226	0	0.1767	0.0631	0	0.0462	0	0.0602	0.0786	0	0.043	0	0.0242	0.1252	0.0549	0.1591	0.0749	0	0	0.0648	0	0.0064	<0.0001	0.5188
GSM972006	0.0263	0	0.1861	0.0174	0	0.0841	0.0165	0.0941	0.0119	0.0202	0.0524	0	0	0.1259	0.0514	0.1426	0.0272	0.0052	0	0.0722	0.0587	0.0077	0.001	0.3508
GSM972005	0.021	0	0.0312	0.019	0.0229	0.07	0	0.035	0.0704	0	0.0488	0	0	0	0.0058	0.0459	0.0699	0	0	0.5419	0.0051	0.013	0.198	0.0801
GSM972004	0	0	0.3029	0.0416	0	0.1284	0.0098	0.1041	0.0111	0	0.0819	0	0	0.0478	0.0632	0.1074	0.0106	0.0228	0	0.0672	0	0.001	<0.0001	0.4699
GSM972003	0	0.0253	0.3313	0	0.0318	0.1156	0.0098	0.0579	0.0146	0	0.12	0	0	0.0119	0.0321	0.0243	0.0194	0.0547	0	0.1425	0	0.0087	<0.0001	0.4168
GSM972002	0.022	0	0.102	0.0279	0.0627	0.0769	0	0	0	0	0.0425	0.0033	0	0.1184	0.0431	0.1187	0.0079	0.025	0	0.2889	0	0.0606	0.007	0.2635
GSM972001	0.0234	0	0.3817	0.0295	0	0.1053	0.0334	0.0307	0.0257	0	0.0926	0	0	0.0601	0.0278	0.0561	0.0136	0.0467	0	0.0594	0.0139	0	<0.0001	0.54
GSM972000	0	0.0921	0.058	0	0.0172	0.0829	0	0.0599	0.0473	0.0499	0	0	0	0.1491	0.0884	0.2319	0.0516	0	0	0.0717	0	0	0.005	0.2792
GSM971999	0.0347	0	0.2197	0.0122	0	0.139	0.0687	0.0637	0	0.0131	0.0806	0	0	0.0796	0.0499	0.1041	0.0014	0.016	0	0.1152	0.0003	0.0017	<0.0001	0.4037
GSM971998	0.0085	0	0.2844	0.0053	0.0098	0.1157	0.0031	0	0.0544	0.0148	0.0637	0	0	0.0296	0.103	0.1743	0.021	0.0039	0.078	0.0304	0	0	0.001	0.3719
GSM971997	0.0952	0	0.1398	0.1307	0	0	0.0956	0.0417	0.0154	0.0375	0.0472	0	0	0.0683	0.1805	0.1111	0.015	0	0	0.0222	0	0	<0.0001	0.6919
GSM971996	0.0316	0	0.1305	0.0859	0	0	0.0546	0.0777	0	0.0108	0.0173	0.0005	0	0.1933	0.0928	0.1806	0	0.0165	0	0.0984	0	0.0094	<0.0001	0.4348
GSM971995	0.0479	0	0.2449	0.0025	0	0.056	0	0.0383	0.0493	0.0765	0	0.0047	0	0.0338	0.0923	0.172	0.0354	0	0	0.1226	0.0238	0	0.001	0.3652
GSM971994	0.0548	0	0.2331	0	0.0144	0.2209	0	0.0179	0	0	0.1148	0	0	0.0264	0.0163	0.0694	0.0597	0.0281	0	0.1327	0.0114	0	0.007	0.2555
GSM971993	0	0.0093	0.2054	0.0523	0	0.0757	0.0664	0.0839	0.0107	0.011	0.024	0	0	0.0275	0.1024	0.1278	0.0702	0.0064	0	0.0806	0.0074	0.039	0.003	0.3023
GSM971992	0.0312	0	0.2695	0	0.051	0.1206	0.0888	0.0136	0.0127	0	0.0795	0	0	0.0543	0.0225	0.0965	0.0028	0.0313	0	0.0985	0	0.0273	0.003	0.3018
GSM971991	0	0	0.1716	0.0531	0	0.0546	0.0103	0.0632	0.0087	0	0.038	0	0	0.32	0.0627	0.0785	0	0	0	0.1261	0	0.0133	<0.0001	0.406
GSM971990	0	0.039	0.2122	0.0654	0	0.0961	0.1012	0.0255	0.0218	0	0.0379	0	0	0.1199	0.075	0.0928	0.0534	0.0008	0.0259	0.007	0.0035	0.0229	0.148	0.0952
GSM971989	0	0	0.3726	0.0193	0	0.2325	0	0.0396	0.0167	0	0.0676	0	0	0.013	0.0172	0.0186	0.0688	0.0426	0	0.0914	0	0	<0.0001	0.4107
GSM971988	0	0.0384	0.1764	0.1011	0	0.1102	0	0.0125	0.0647	0	0.1058	0	0	0.0445	0.0454	0.118	0.0443	0.0342	0	0.1045	0	0	0.01	0.2278
GSM971987	0.0372	0	0.2332	0.0376	0.0288	0.1398	0	0.0355	0	0.0863	0	0	0	0.0204	0.0255	0.1078	0.0809	0.0269	0.0719	0.0229	0.0454	0	<0.0001	0.5202
GSM971986	0.0209	0	0.1435	0.0205	0.0332	0.0662	0.0403	0.0549	0.0289	0	0.1186	0	0	0.1146	0.0811	0.1268	0	0	0	0.1504	0	0	<0.0001	0.4514
GSM971985	0.025	0	0.3028	0.0362	0	0.2931	0.0152	0	0.0384	0	0.0739	0	0.0004	0.0203	0.0111	0.0501	0.0364	0.0223	0	0.0748	0	0	<0.0001	0.4918
GSM971984	0	0.0031	0.1216	0.1055	0	0	0.1008	0.0784	0	0.0408	0.0503	0	0	0.0974	0.1668	0.1599	0.0332	0	0	0.0404	0	0.0016	<0.0001	0.4242
GSM971983	0.0189	0	0.0773	0.0538	0	0.0439	0.0398	0.087	0.0049	0.0157	0.0274	0	0	0.1449	0.1123	0.1435	0.0088	0.0023	0	0.1427	0.0094	0.0674	<0.0001	0.4471
GSM971982	0.0038	0	0.057	0.0514	0	0	0.063	0.0507	0.0172	0	0.0658	0	0	0.1494	0.1811	0.1931	0.0203	0	0	0.0861	0	0.061	0.004	0.2927
GSM971981	0.0144	0	0.1338	0.1602	0	0	0.058	0.0565	0.0146	0.0139	0.0081	0.0002	0	0	0.1398	0.2421	0.0503	0.0021	0	0.0803	0	0.0259	<0.0001	0.5476
GSM971980	0.0429	0	0.2084	0.0373	0	0.0542	0.0797	0.0734	0	0.0851	0	0	0	0.0166	0.1513	0.0775	0.0179	0.0349	0.0009	0.0552	0.0176	0.047	0.001	0.3228
GSM971979	0.012	0	0.0293	0	0	0.0877	0	0.0161	0	0.0029	0.0213	0	0	0.2271	0.0215	0.0481	0	0.0106	0	0.4484	0.0017	0.0735	<0.0001	0.3961
GSM971978	0.0306	0	0.1555	0.1323	0	0	0.1453	0.0906	0	0.0097	0.0671	0.0027	0	0.0217	0.1178	0.1316	0.0432	0	0	0.0437	0	0.0081	<0.0001	0.4538
GSM971977	0.0128	0	0.1024	0.0318	0	0.0425	0.0369	0.1051	0.0374	0.0191	0.0307	0	0	0.1778	0.1072	0.1685	0.0291	0	0	0.0458	0.0098	0.0433	<0.0001	0.455
GSM971976	0.0153	0	0.1773	0	0.0103	0.1779	0.1181	0.002	0	0	0.1528	0	0	0.0465	0.0564	0.021	0.0592	0.0375	0	0.1173	0	0.0085	0.087	0.122
GSM971975	0	0.0176	0.161	0.0134	0.0332	0.0483	0.0683	0.0427	0	0	0.1047	0	0	0.2039	0.0157	0.0991	0.0057	0.0138	0	0.1342	0	0.0384	0.018	0.1853
GSM971974	0.0495	0	0.222	0.0101	0.0476	0.1788	0	0.0221	0.0278	0	0.1117	0	0.0152	0.0741	0.0089	0.011	0.0017	0.048	0	0.1714	0	0	0.011	0.214
GSM971973	0.0254	0	0.035	0.0921	0	0	0	0.1073	0.0336	0	0.0632	0	0	0.1838	0.044	0.1863	0.0284	0.0087	0	0.1337	0	0.0585	0.01	0.2223
GSM971972	0	0	0.2024	0.1286	0	0.0496	0.0844	0.0643	0	0	0.0854	0	0.0235	0.0535	0.0975	0.1461	0.0445	0	0.0034	0.0169	0	0	0.004	0.2962
GSM971971	0.0087	0	0.1431	0.112	0	0.0076	0.0465	0.1019	0.0339	0.035	0.0171	0.0046	0	0.0973	0.1235	0.113	0.0498	0.0086	0	0.0963	0	0.0011	<0.0001	0.3957
GSM971970	0	0.0031	0.0714	0.0362	0	0.0673	0.0587	0.1052	0	0	0.1405	0	0.0297	0.0975	0.077	0.0862	0.0025	0.0199	0	0.188	0	0.0168	0.043	0.1551
GSM971969	0.0236	0.025	0.1563	0.0761	0	0.0247	0.0529	0.0616	0.0161	0.0722	0.0366	0	0	0.1337	0.0979	0.175	0.0303	0.0056	0.0108	0	0	0.0017	<0.0001	0.5075
GSM971968	0.0044	0	0.0605	0.0229	0	0.0705	0.0182	0.0295	0.0322	0.0193	0.0037	0	0.0407	0.2827	0.0415	0.2011	0.0598	0.0118	0.0513	0.0121	0.0106	0.0272	<0.0001	0.429
GSM971967	0.0011	0	0.0333	0	0	0.2048	0	0.0334	0.0223	0	0.0883	0	0	0.0558	0.03	0.1365	0.0551	0.0311	0	0.26	0	0.0484	0.019	0.1844
GSM971966	0.064	0	0.1465	0.0195	0	0.0231	0	0.0507	0.0276	0	0.1238	0	0	0.0352	0.0578	0.121	0.0462	0.0422	0	0.1602	0	0.0823	0.001	0.3213
GSM971965	0.0196	0	0.2697	0	0.0371	0.1089	0.0023	0.0049	0.0171	0	0.0875	0	0	0.1459	0.0285	0.2045	0	0.0306	0	0.0436	0	0	<0.0001	0.3797
GSM971964	0.019	0	0.2872	0	0	0.172	0.0285	0.0374	0	0	0.0896	0	0	0.0282	0	0.0783	0.0263	0.049	0	0.1447	0.0281	0.0118	0.001	0.3177
GSM971963	0.0037	0	0.087	0.2236	0	0	0.0951	0.0441	0.0152	0.0511	0.0307	0	0	0.1223	0.1737	0.1204	0.0047	0	0	0.028	0	0.0004	<0.0001	0.5945
GSM971962	0.0258	0	0.1486	0.0136	0	0.0093	0	0.0458	0.038	0.0261	0.0586	0	0	0.2249	0.0576	0.1408	0	0	0	0.1878	0.0146	0.0084	<0.0001	0.5389
GSM971961	0.0052	0	0.1549	0.0103	0	0.005	0.001	0.0359	0.0163	0.0097	0.0107	0	0.0219	0.4176	0.0199	0.2169	0	0	0	0.0547	0	0.0199	<0.0001	0.7274
GSM971960	0.0557	0	0.3202	0	0.0551	0.0776	0.0818	0.0246	0.031	0	0.0987	0	0.0183	0.0672	0.0196	0.0592	0.0224	0.0161	0.0034	0.0493	0	0	<0.0001	0.491
GSM971959	0	0.021	0.2129	0	0.0111	0	0	0.056	0.0412	0	0.0359	0	0	0.0701	0.013	0.1073	0.0132	0.0155	0	0.3903	0.006	0.0067	0.009	0.2361
GSM971958	0	0	0.1427	0.0354	0.0223	0	0.0214	0.023	0	0.0496	0.0295	0	0	0.2939	0.0272	0.1898	0.0015	0.0021	0	0.1268	0	0.035	<0.0001	0.6255
GSM971957	0.0231	0	0.0954	0.0596	0	0	0.0636	0.0661	0.0327	0	0.0386	0.0219	0	0.1542	0.0668	0.0465	0.0305	0.0289	0	0.1994	0.0178	0.0549	0.008	0.2447

CIBERSORT scores: 0.5, 0.4, 0.3, 0.2, 0.1, 0.0

Supplementary Figure S1. Continued

Input Samples	B cells naive	B cells memory	Plasma cells	T cells CD8	T cells CD4 naive	T cells CD4 memory resting	T cells CD4 memory activated	T cells follicular helper	T cells regulatory (Tregs)	T cells gamma delta	NK cells resting	NK cells activated	Monocytes	Macrophages M0	Macrophages M1	Macrophages M2	Dendritic cells resting	Dendritic cells activated	Mast cells resting	Mast cells activated	Eosinophils	Neutrophils	P-value	Pearson correlation
GSM972156	0.0302	0	0.2689	0	0	0.1437	0	0.0186	0	0.0227	0.0632	0	0	0.0141	0.0152	0.1335	0.0448	0.0215	0	0.1884	0.0309	0.0044	0.001	0.3394
GSM972155	0.0502	0	0.2009	0	0.0753	0.0806	0.1006	0.0394	0	0	0.1319	0	0	0	0	0.0679	0.0455	0.0462	0	0.1193	0.0207	0.0214	0.088	0.1215
GSM972154	0	0	0.171	0	0.0308	0.2979	0	0	0	0	0.0884	0	0	0.0525	0.0572	0.0891	0.0224	0.0463	0	0.1444	0	0	0.042	0.1573
GSM972153	0	0.0293	0.295	0.007	0	0.091	0	0.1249	0.0089	0	0.0121	0.0161	0	0.0068	0.0608	0.0966	0.0396	0.0304	0	0.0937	0.0601	0.0277	<0.0001	0.4373
GSM972152	0.0415	0	0.2311	0	0.0214	0.0521	0.0997	0.028	0.0173	0.0135	0.0713	0	0	0.0981	0.0205	0.0656	0.0275	0.0303	0	0.1819	0	0	0.004	0.2886
GSM972151	0	0.0246	0.2728	0.0323	0	0.1547	0.0538	0.0186	0	0	0.0736	0	0.0051	0.1109	0.0451	0.0944	0.0209	0.0402	0	0.0531	0	0	0.001	0.3443
GSM972150	0.0194	0	0.34	0	0	0.1518	0.0168	0.0261	0.0128	0	0.0887	0	0	0.0501	0.0389	0.097	0.0418	0.0323	0.0338	0.035	0.0004	0.015	<0.0001	0.4768
GSM972149	0	0.0331	0.2895	0	0.0695	0.124	0	0	0.0127	0	0.0732	0	0	0.0181	0.0326	0.1182	0.0005	0.0336	0	0.1866	0	0.0083	0.001	0.3566
GSM972148	0	0	0.1033	0.162	0.014	0	0.0825	0.101	0.0075	0.0102	0.0202	0	0	0.072	0.0999	0.1749	0.0269	0.035	0	0.0696	0.0059	0.0151	0.01	0.2212
GSM972147	0	0.0303	0.055	0.0267	0.036	0	0.1256	0.0971	0	0.0051	0.0914	0	0	0.0506	0.1567	0.1838	0.0065	0.0356	0	0.0809	0	0.0187	0.025	0.1782
GSM972146	0.0261	0	0.2601	0.0348	0	0.126	0.0089	0.0064	0.0176	0	0.0524	0	0	0.109	0.0565	0.1162	0.0178	0.0107	0	0.1132	0	0.0443	<0.0001	0.4628
GSM972145	0.067	0	0.2487	0	0	0.1517	0	0.0758	0.0269	0.0195	0.0675	0	0	0.0637	0.0621	0.0973	0.0022	0	0	0.1123	0.0051	0	0.003	0.3037
GSM972144	0.0463	0	0.2927	0	0.0798	0.1605	0.0782	0	0	0	0.1492	0	0	0.0088	0	0.0511	0.0277	0.0166	0	0.0815	0	0.0076	0.008	0.2403
GSM972143	0	0	0.1474	0.0471	0	0.0644	0.0424	0.0867	0.0117	0	0.0709	0	0	0.1158	0.117	0.0852	0.0246	0.0188	0	0.1395	0	0.0285	0.008	0.2478
GSM972142	0.0421	0	0.2292	0	0	0.2032	0.0044	0.0188	0.028	0.0091	0.0905	0	0.0138	0.0253	0.0533	0.0529	0.0179	0.0369	0	0.1508	0.0237	0	0.005	0.2733
GSM972141	0.007	0.0024	0.0123	0	0.0384	0.1119	0.0818	0.0034	0	0	0.0945	0	0	0.1423	0.068	0.0529	0.0227	0.0136	0	0.2192	0.0055	0.124	0.059	0.138
GSM972140	0.0068	0	0.0297	0	0	0.0758	0	0.039	0.026	0.0103	0.0448	0	0	0.3559	0.0613	0.2342	0	0	0	0.1059	0	0.0104	<0.0001	0.4506
GSM972139	0	0.0005	0.2635	0	0.0306	0.1019	0.0445	0.0221	0.0177	0	0.1028	0	0	0.1135	0	0.1453	0	0.0534	0	0.0946	0	0.0097	0.008	0.2386
GSM972138	0.0327	0	0.3491	0.1393	0	0.0142	0.0014	0.0384	0.0307	0	0.0206	0	0	0	0.0212	0.1734	0.0347	0.03	0	0.1113	0	0.0031	0.001	0.3714
GSM972137	0	0	0.2919	0.0447	0	0.1254	0.011	0.0053	0.0409	0	0.0824	0	0	0.1099	0.0294	0.0923	0.0278	0.0228	0	0.1163	0	0	0.001	0.3665
GSM972136	0.0441	0	0.1768	0	0.0255	0.2345	0.0025	0	0.0176	0	0.0966	0	0	0.1114	0.0163	0.0984	0.0096	0.0285	0	0.1195	0	0.0186	0.123	0.1051
GSM972135	0	0.0565	0.089	0.096	0	0.059	0	0	0.1083	0	0.1212	0	0	0	0.0138	0.1773	0.1044	0.0321	0	0.1423	0	0	0.077	0.1259
GSM972134	0.0418	0.0348	0.2074	0	0.0124	0.0471	0	0.0782	0	0.0071	0.045	0	0	0.0536	0.0492	0.0945	0.0175	0.0381	0	0.1609	0.0613	0.0509	0.004	0.2982
GSM972133	0	0	0.2713	0.0364	0	0.1038	0	0.0768	0.0091	0	0.0021	0.0114	0	0.1028	0.0563	0.2079	0.0284	0.0124	0	0.0645	0.0032	0.0136	<0.0001	0.4041
GSM972132	0.0616	0	0.1374	0	0	0.1329	0.0722	0.0251	0	0	0.0912	0	0	0.2359	0.0373	0.0823	0.0088	0	0.0029	0.1123	0	0	0.007	0.2584
GSM972131	0.0393	0	0.2489	0.009	0.0039	0.0126	0	0.081	0.0231	0.0689	0.0195	0	0	0.2715	0.0647	0.1016	0	0.0003	0	0.0534	0	0.0023	<0.0001	0.5354
GSM972130	0	0.0126	0.0442	0	0.0974	0.1071	0.0934	0.0081	0.0551	0	0.1206	0	0	0.18	0.0836	0	0.0764	0	0	0.1215	0	0	0.133	0.1005
GSM972129	0.0015	0	0.3284	0.0785	0	0	0.0076	0.1194	0.0121	0	0.0288	0	0	0.0699	0.0742	0.1315	0.0599	0.0036	0	0.0667	0.0141	0.0039	<0.0001	0.4828
GSM972128	0	0	0.1228	0	0.0142	0.1242	0.0096	0.0166	0.0327	0	0.0976	0	0	0.2154	0.0395	0.1015	0.0116	0.0024	0	0.1591	0	0.0529	<0.0001	0.3843
GSM972127	0	0.0203	0.2618	0.0257	0	0.0327	0.0409	0.102	0.0048	0	0.1063	0	0	0.0509	0.0424	0.168	0.0265	0.0206	0	0.091	0.0061	0	<0.0001	0.4175
GSM972126	0	0.1406	0.1188	0.0133	0	0.083	0.1206	0.0427	0	0	0.139	0	0.0317	0.065	0.0523	0	0.0109	0.0578	0	0.1244	0	0	0.052	0.1445
GSM972125	0.0048	0	0.3169	0.063	0	0.0828	0	0.031	0	0	0.0614	0	0	0.109	0.0191	0.1821	0.0187	0.0111	0.0308	0.0532	0.0122	0.0038	<0.0001	0.4975
GSM972124	0.0115	0	0.2447	0.0518	0.0173	0.0774	0	0.01	0.0179	0.0198	0.053	0	0	0.0857	0.0641	0.2184	0.032	0.0006	0.0451	0.0509	0	0	<0.0001	0.4708
GSM972123	0.0316	0	0.2189	0.0113	0.1043	0.0298	0.1133	0.0595	0	0	0.148	0	0	0.0846	0.0446	0.0048	0.0202	0.0481	0	0.0809	0	0	0.005	0.2809
GSM972122	0	0.0269	0.1757	0.1213	0.0316	0.0589	0	0.0198	0.0208	0	0.0842	0	0	0.0258	0.0193	0.1542	0.0455	0.0358	0	0.1804	0	0	0.071	0.1298
GSM972121	0.0088	0	0.4249	0.0479	0	0.1119	0	0.0381	0.0317	0	0.0877	0	0	0.0667	0.0194	0.0694	0	0.0099	0	0.0825	0	0.0009	<0.0001	0.454
GSM972120	0.0235	0	0.1868	0.0271	0	0	0.0574	0.1011	0.0279	0.0122	0.0693	0	0	0.099	0.1165	0.1256	0.0246	0.0412	0	0.0706	0	0.0172	<0.0001	0.4714
GSM972119	0.0618	0	0.1457	0.0265	0	0.1379	0.1288	0.0382	0.0421	0	0.1017	0	0	0.0435	0.0095	0.0876	0.0531	0.0058	0	0.0944	0.0073	0.016	0.165	0.0903
GSM972118	0	0	0.1813	0	0.0755	0.0708	0	0.0017	0.09	0.0001	0.0996	0	0	0.0311	0	0	0.092	0.0662	0	0.216	0.0759	0	0.031	0.1698
GSM972117	0.0407	0	0.1465	0.07	0	0.0681	0.1169	0.0707	0	0.0632	0.0517	0	0	0.0632	0.1465	0.107	0.0167	0.0124	0	0.0264	0	0	<0.0001	0.4612
GSM972116	0	0	0.3793	0.0466	0	0.1941	0	0.0176	0.0065	0	0.0474	0	0	0	0.0119	0.0944	0.0765	0.0203	0.0505	0.055	0	0	<0.0001	0.4043
GSM972115	0	0.061	0.1633	0	0.1026	0.0762	0.0765	0.0353	0	0.0009	0.1001	0	0	0.0483	0.0139	0.07	0.0486	0.0389	0	0.1252	0	0.0391	0.035	0.1638
GSM972114	0	0.0477	0.1601	0.0059	0.0792	0.0651	0	0.0066	0.0174	0	0.029	0	0	0.1025	0.0102	0.0836	0.0069	0.0345	0	0.252	0.0024	0.0971	0.001	0.3185
GSM972113	0	0.0067	0.2349	0.0431	0	0.1407	0.0005	0.0198	0.0147	0.0559	0.004	0	0	0.1445	0.0253	0.2144	0.0296	0.004	0	0.0405	0	0.0213	<0.0001	0.5567
GSM972112	0	0.0044	0.1058	0.0189	0	0	0	0.0706	0.0332	0.0964	0	0	0	0.2392	0.0728	0.1673	0.031	0.0017	0	0.1332	0.0012	0.0245	<0.0001	0.4231
GSM972111	0	0.0146	0.4423	0.1283	0	0.0495	0.0182	0.0116	0.0244	0	0.0589	0	0	0.0122	0.0202	0.0732	0.0404	0.0052	0	0.101	0	0	<0.0001	0.5813
GSM972110	0	0	0.3456	0.0557	0	0.1131	0.0238	0.0509	0.0026	0	0.068	0	0	0.0297	0.0481	0.143	0.027	0.0197	0	0.0685	0	0.0043	<0.0001	0.5506
GSM972109	0	0.0457	0.1126	0.022	0	0.0731	0.0373	0.0486	0	0	0.0908	0	0	0.2133	0.0735	0.1039	0.0118	0	0	0.1455	0	0.022	0.049	0.1483
GSM972108	0.0094	0	0.2177	0.0321	0	0.1069	0.0788	0.0518	0.0212	0.0229	0.0681	0	0	0.0879	0.0665	0.0991	0.0336	0.0011	0	0.1031	0	0	0.033	0.1674
GSM972107	0.012	0	0.2185	0.0865	0.0009	0	0.0357	0.0944	0.0091	0.0103	0.0655	0	0.0087	0.121	0.0499	0.1177	0.0151	0.023	0	0.1105	0	0.0211	<0.0001	0.4239
GSM972106	0.0263	0	0.0808	0.1329	0.1255	0	0	0.0196	0.0081	0	0.1033	0	0	0.0238	0.0722	0.1985	0.0855	0.0527	0	0.0709	0	0	0.037	0.1614
GSM972105	0	0.0326	0.1378	0.12	0	0.0244	0	0.0758	0.0444	0	0.1083	0	0	0.1345	0.0391	0.1704	0	0.033	0	0.0798	0	0	0.009	0.2328
GSM972104	0.0046	0	0.2908	0	0	0.1424	0.0176	0.0288	0.0188	0.0283	0.047	0	0	0.08	0.0511	0.1099	0.0395	0	0.0477	0.0613	0.0229	0.0092	<0.0001	0.583
GSM972103	0.0025	0	0.222	0.0929	0	0.0079	0.0451	0.0706	0.0461	0	0.0745	0	0	0.0151	0.1713	0.1571	0.0353	0.0129	0	0.0467	0	0	<0.0001	0.4876
GSM972102	0.0269	0	0.3176	0.0601	0	0.1155	0.0015	0.0625	0	0.0743	0.0547	0	0	0.0113	0.0501	0.1369	0.0067	0.0001	0	0.0703	0.0114	0	<0.0001	0.496
GSM972101	0.0026	0	0.2945	0.0297	0	0.2248	0	0.046	0.0004	0.0258	0.0463	0	0	0	0.0443	0.0824	0.11	0.015	0	0.0676	0.0106	0	<0.0001	0.3883
GSM972100	0	0	0.2029	0.0905	0	0.0095	0	0.0733	0.0339	0	0.0541	0	0	0.1298	0.038	0.1288	0.0065	0.0364	0	0.1854	0	0.0108	0.198	0.08
GSM972099	0	0	0.1954	0.0932	0.0598	0.0114	0.1614	0.0253	0	0	0.0854	0	0.0239	0	0	0.0741	0.0614	0.031	0	0.1468	0.0309	0	0.074	0.1286
GSM972098	0	0.0227	0.1671	0.1646	0	0.1623	0	0.0486	0.0494	0	0.0337	0.0028	0	0	0.0116	0.1713	0.0836	0.0371	0	0.0452	0	0	0.052	0.1446
GSM972097	0.0218	0	0.1909	0.0156	0.0383	0.1658	0	0.0814	0	0	0.0658	0	0	0.0567	0.0308	0.2103	0.0694	0	0	0.0532	0	0	0.001	0.3658
GSM972096	0	0.1411	0.1207	0	0.1834	0.0945	0	0.0039	0.0651	0	0.02	0	0	0.071	0.0175	0	0.0456	0.0227	0	0.1969	0	0.0176	0.052	0.1442
GSM972095	0.0112	0	0.1204	0.0082	0	0.0176	0.0244	0.0831	0.0193	0.006	0.0763	0	0	0.1575	0.1239	0.1367	0.028	0.0252	0	0.1483	0	0.0139	0.006	0.2665
GSM972094	0.0076	0	0.4499	0.0939	0	0.0124	0.0423	0.0234	0	0.0165	0.0285	0	0	0.1144	0.0584	0.0643	0	0.0169	0	0.0543	0	0.0174	<0.0001	0.6597
GSM972093	0.0229	0	0.3023	0	0	0.1013	0.0295	0.0615	0	0	0.0951	0	0	0.1086	0.0338	0.1029	0.0173	0.0198	0	0.0646	0.009	0.0314	0.001	0.3383
GSM972092	0	0.0248	0.0805	0.0288	0	0.0643	0.0122	0.0966	0.0441	0	0.0091	0.0203	0	0.135	0.1523	0.1905	0.0796	0.006	0.0198	0.0227	0	0.0133	<0.0001	0.388
GSM972091	0.0587	0	0.1647	0	0.0539	0.014	0.1395	0.0755	0.0362	0.0194	0.0882	0	0	0.0363	0.0239	0.0391	0.0388	0.0461	0	0.1658	0	0	0.052	0.145
GSM972090	0	0.0039	0.1491	0.0321	0	0.1619	0	0	0	0	0.1139	0	0	0	0.0163	0.2291	0.1116	0.0544	0	0.1276	0	0	0.413	0.0386
GSM972089	0.0718	0	0.136	0.0338	0	0.0761	0	0.0819	0	0	0.0513	0	0.0085	0	0.1008	0.2327	0.0651	0.0186	0.0473	0.0578	0	0.0183	<0.0001	0.4386
GSM972088	0.1736	0	0.3255	0	0	0.0491	0	0.0786	0.0005	0.0751	0.0382	0	0	0	0.0287	0.1028	0	0.0282	0	0.083	0.0168	0	<0.0001	0.547
GSM972087	0.0394	0	0.2799	0.0102	0.0529	0.0832	0.0326	0.0259	0	0.0291	0.075	0	0	0.108	0.0509	0.0264	0	0.031	0	0.1556	0	0	0.005	0.2798
GSM972086	0.02	0	0.2314	0.0556	0	0	0	0.1359	0.0545	0.0075	0.0321	0	0	0.0978	0.1271	0.0623	0.0518	0.0085	0	0.0979	0.0177	0	0.001	0.3648
GSM972085	0.0141	0	0.3197	0.045	0	0.1271	0.0668	0.018	0.0028	0	0.0626	0	0	0.055	0.0329	0.084	0.024	0.0301	0	0.0598	0	0.0582	<0.0001	0.4491
GSM972084	0.0112	0.0066	0.304	0.0263	0	0.2504	0.0147	0.0349	0	0.0242	0.0289	0	0	0	0.0318	0.1409	0.0358	0.0179	0.0108	0.0414	0.0161	0.0042	<0.0001	0.5304
GSM972083	0.0064	0	0.1626	0.04	0	0.1729	0.0112	0.0793	0.0247	0	0.018	0.0001	0	0.0503	0.0776	0.1387	0.0265	0.033	0	0.1282	0.001	0.0294	<0.0001	0.4699
GSM972082	0.0133	0	0.2242	0.0427	0	0.142	0.0201	0.0414	0	0.0296	0.0257	0	0	0.0641	0.0574	0.1797	0.036	0.0173	0	0.099	0	0.0075	<0.0001	0.5009
GSM972081	0.0358	0	0.339	0.018	0	0.1688	0	0.0005	0.0263	0	0.0549	0	0	0.0078	0.0398	0.1301	0.0768	0.0277	0	0.0663	0	0.008	<0.0001	0.4516
GSM972080	0.0208	0	0.1784	0.0225	0	0.0992	0	0.0152	0.0063	0	0.058	0	0	0.1911	0.0783	0.1275	0.0117	0	0	0.1351	0	0.0559	0.001	0.3189
GSM972079	0.0271	0	0.1702	0.1561	0	0	0.0132	0.0309	0.0486	0	0.0814	0	0.005	0.0873	0.043	0.1683	0.0441	0	0	0.1	0	0.0246	<0.0001	0.5159
GSM972078	0.0075	0	0.1396	0.0003	0.0153	0.0552	0.0011	0.0534	0.0311	0	0.057	0	0	0.2386	0.0686	0.1496	0.033	0.0074	0	0.1173	0	0.0249	<0.0001	0.3807
GSM972077	0.0394	0	0.3467	0.0641	0.0462	0	0	0.0533	0.0535	0.0072	0.0632	0	0	0.0337	0.0165	0.1019	0.0329	0	0	0.1348	0	0.0067	<0.0001	0.6166
GSM972076	0.0132	0	0.1126	0.0912	0	0.0346	0.0483	0.0642	0.024	0	0.0633	0	0	0.1468	0.1204	0.1276	0.0407	0	0	0.0906	0.0035	0.0191	<0.0001	0.4267
GSM972075	0	0	0.2113	0.083	0	0.1091	0.0345	0.0159	0.0126	0.0198	0.0397	0	0	0.1474	0.0628	0.1482	0.0348	0	0	0.068	0	0.0129	0.001	0.3327
GSM972074	0.0185	0	0.222	0	0	0.1237	0.086	0.031	0	0	0.0819	0	0	0.1275	0.025	0.0965	0.0465	0.0399	0	0.0795	0	0.0219	0.005	0.2808
GSM972073	0	0.0039	0.2333	0.0014	0	0.0719	0.0616	0.0561	0	0.021	0.0415	0	0	0.1704	0.0882	0.156	0.0155	0.007	0	0.0472	0	0.0252	<0.0001	0.4887
GSM972072	0	0	0.1424	0.0479	0	0.0466	0.0744	0.0826	0.0121	0.1129	0.0079	0.0097	0	0.0907	0.1353	0.1464	0.0405	0.0238	0.0034	0.0079	0	0.0156	<0.0001	0.5149
GSM972071	0.0342	0	0.0903	0	0.0104	0.1338	0	0.0187	0.037	0.0088	0.0747	0	0	0.2717	0.0219	0.107	0.0054	0.0165	0	0.1696	0	0	0.004	0.2823
GSM972070	0.0102	0	0.3608	0.0103	0	0.1731	0	0	0.0248	0.0033	0.0411	0	0	0.0083	0.0114	0.1735	0.053	0.0215	0.0663	0.0257	0.0133	0.0035	<0.0001	0.498
GSM972069	0.0342	0	0.312	0.0035	0	0.1136	0.0451	0.0555	0.0056	0	0.0718	0	0	0.101	0.0298	0.0992	0.0137	0.0137	0	0.052	0.013	0.0364	0.001	0.364
GSM972068	0	0	0.1406	0.043	0	0.0226	0	0.0303	0.019	0.028	0.0335	0	0	0.2859	0.0293	0.1908	0.0326	0	0	0.1149	0	0.0295	<0.0001	0.6134
GSM972067	0.0287	0	0.2558	0.0693	0	0.0512	0.0278	0.0784	0.0274	0.0272	0.0378	0	0	0.0394	0.0801	0.161	0.0657	0	0	0.0414	0	0.0089	<0.0001	0.5861
GSM972066	0.0466	0	0.2674	0	0.0164	0.1314	0.0419	0.0117	0	0	0.0691	0	0	0.1468	0.0251	0.0854	0	0.0153	0	0.1191	0	0.0239	<0.0001	0.4543
GSM972065	0.0359	0	0.2642	0.0281	0	0.093	0.0686	0.0239	0	0	0.0482	0	0	0.0553	0.033	0.1593	0.0458	0.0278	0	0.078	0.0136	0.0251	<0.0001	0.3837
GSM972064	0	0.0162	0.1922	0.026	0	0.0239	0.02	0.0514	0.0017	0.0042	0.0397	0	0	0.1269	0.0389	0.1411	0.025	0	0	0.2277	0	0.065	<0.0001	0.5645
GSM972063	0.0286	0	0.2174	0	0.0141	0.1256	0.0512	0.0061	0	0	0.0711	0	0	0.0929	0.0473	0.1381	0.057	0.0212	0	0.1021	0.0151	0.0123	<0.0001	0.4418
GSM972062	0.0265	0	0.2532	0.0353	0	0.1192	0.0189	0.0262	0	0	0.0375	0	0.0127	0.0297	0.014	0.1547	0.1041	0.0184	0	0.1339	0	0.0159	<0.0001	0.438
GSM972061	0	0.0019	0.2909	0.1284	0.0096	0.0097	0.0455	0.0224	0.0208	0	0.0832	0	0	0.1377	0.0385	0.1033	0	0	0	0.1061	0	0.0022	<0.0001	0.5043
GSM972060	0.0261	0	0.1071	0.0952	0	0.0244	0.0625	0.0669	0.0347	0	0.0313	0	0	0.0818	0.1011	0.231	0.0857	0.0157	0	0.0345	0	0.0022	<0.0001	0.427
GSM972059	0.0348	0	0.2297	0.1048	0.0074	0	0.024	0.0445	0.0059	0	0.1196	0	0.0001	0.0853	0.0681	0.1246	0.0201	0.0051	0	0.1116	0	0.0145	<0.0001	0.5629
GSM972058	0.0419	0	0.106	0	0	0.1346	0.033	0.0502	0	0.0062	0.0523	0	0	0.1126	0.0573	0.1042	0.0041	0.0264	0	0.1592	0	0.1118	<0.0001	0.3856
GSM972057	0	0	0.1528	0.1084	0	0	0.0305	0.0496	0.0299	0	0.0824	0	0	0	0.0442	0.2035	0.0338	0.0305	0	0.17	0	0.0644	0.02	0.1819

CIBERSORT scores

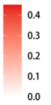

Supplementary Figure S1. Continued

Input Samples	B cells naive	B cells memory	Plasma cells	T cells CD8	T cells CD4 naive	T cells CD4 memory resting	T cells CD4 memory activated	T cells follicular helper	T cells regulatory (Tregs)	T cells gamma delta	NK cells resting	NK cells activated	Monocytes	Macrophages M0	Macrophages M1	Macrophages M2	Dendritic cells resting	Dendritic cells activated	Mast cells resting	Mast cells activated	Eosinophils	Neutrophils	P-value	Pearson correlation
GSM972256	0.0458	0	0.2689	0.0605	0.0718	0	0.0174	0.0527	0	0	0.1317	0	0	0.0652	0.0044	0.0348	0.0242	0.0247	0	0.1696	0	0.0282	0.019	0.1837
GSM972255	0	0.0078	0.0967	0.0573	0	0.1176	0	0.0775	0.0302	0	0.0542	0	0	0.2028	0.0344	0.2143	0.0131	0	0	0.0775	0	0.0166	0.267	0.0632
GSM972254	0.0083	0	0.1332	0	0.0055	0.002	0	0.0567	0.0686	0.022	0.0629	0	0	0.3845	0.029	0.074	0	0.0097	0	0.1397	0	0.0039	<0.0001	0.4796
GSM972253	0.0194	0	0.3382	0.0066	0.0627	0.0549	0.0293	0.0235	0	0	0.1005	0	0	0.0602	0.0185	0.1005	0.0239	0.0463	0	0.1065	0	0.0088	<0.0001	0.4457
GSM972252	0.0147	0	0.1847	0.0737	0	0.0311	0.0063	0.1253	0.0317	0.0018	0.0477	0	0.0012	0	0.0836	0.2222	0.0667	0	0	0.1036	0	0.0058	<0.0001	0.4889
GSM972251	0.0547	0	0.0739	0	0	0.1012	0.0667	0.0684	0.0453	0.0531	0	0.0236	0	0.068	0.0644	0.1396	0.0282	0.0245	0.0768	0.0591	0.0244	0.0279	0.082	0.1244
GSM972250	0.0335	0	0.188	0.1202	0	0.0736	0.0948	0.0131	0	0	0.0897	0	0.0375	0.1193	0.009	0.0701	0.0531	0.004	0	0.0942	0	0	0.012	0.2118
GSM972249	0	0	0.2968	0.0681	0	0.0774	0.0587	0.0501	0	0.0186	0.0348	0	0	0.0414	0.0783	0.1782	0.0414	0	0	0.0489	0	0.0074	0.005	0.2725
GSM972248	0	0	0.3688	0.0509	0	0.0427	0	0.0469	0.0484	0	0.0825	0	0	0.0262	0.0375	0.1397	0.0122	0.0276	0	0.109	0	0.0075	<0.0001	0.4542
GSM972247	0	0.0042	0.1437	0.0751	0	0.0336	0	0.0394	0.0224	0	0.0446	0	0	0.2697	0.0511	0.1047	0.0249	0.0018	0	0.1848	0	0	0.003	0.3044
GSM972246	0	0.0539	0.1267	0.0719	0.0048	0.0741	0.0594	0.0383	0	0	0.1166	0	0	0.0806	0.0507	0.1218	0.024	0.0491	0	0.1128	0	0.0153	0.025	0.1784
GSM972245	0	0	0.329	0.071	0	0.0252	0	0.0431	0.0509	0	0.0505	0	0	0.0297	0.0404	0.1794	0.018	0.026	0	0.1258	0	0.011	<0.0001	0.4275
GSM972244	0.0415	0	0.4138	0.0894	0.0447	0.0208	0.0249	0.016	0.0115	0	0.048	0	0	0.0254	0.0504	0.0773	0.0023	0.0063	0	0.1138	0	0.014	<0.0001	0.5895
GSM972243	0.01	0	0.3022	0.048	0	0.1508	0.038	0.0349	0	0.0344	0.0372	0	0	0.0307	0.0469	0.0926	0.074	0.0112	0	0.0736	0	0.0156	0.001	0.3343
GSM972242	0	0	0.092	0.102	0	0.0875	0.0678	0.0645	0.0204	0	0.0517	0	0	0.0938	0.0923	0.1773	0.0778	0	0	0.0661	0	0.0067	0.001	0.3584
GSM972241	0	0.0492	0.0709	0	0	0.1632	0.0376	0.0474	0.0143	0.0692	0.1304	0	0.0068	0.0967	0.1057	0.0703	0.0311	0	0	0.1048	0.0023	0	0.016	0.1999
GSM972240	0	0.0132	0.0546	0.0979	0	0.0094	0.013	0.0886	0.0326	0	0	0.0267	0	0.1542	0.175	0.238	0.0614	0	0.0032	0.0103	0	0.0219	<0.0001	0.5045
GSM972239	0	0	0.2739	0.0634	0	0.0539	0.045	0.0359	0.0216	0.0198	0	0.0089	0	0.1702	0.082	0.133	0.0446	0	0	0.0359	0	0.012	<0.0001	0.6064
GSM972238	0.0187	0	0.193	0.0142	0	0.047	0.0053	0.0888	0.029	0	0.0286	0	0	0.1203	0.1106	0.1802	0.0331	0.032	0.0486	0.011	0	0.0396	0.001	0.3178
GSM972237	0	0.0168	0.1492	0.0291	0.0178	0.1114	0.0306	0.0533	0.0458	0	0.0361	0.0193	0	0.1113	0.0329	0.1131	0.058	0.0626	0.0378	0.0715	0	0.0035	0.016	0.2004
GSM972236	0	0	0.1308	0.0919	0.0688	0	0.0044	0.0497	0.0349	0	0.0286	0	0	0.1185	0.0565	0.1947	0.0561	0.0219	0	0.1239	0	0.0193	0.008	0.2433
GSM972235	0.0147	0	0.1094	0	0.023	0.0751	0.0962	0.0504	0.0257	0.0031	0.0779	0	0	0.129	0.0461	0.2298	0.0104	0	0	0.1093	0	0	0.007	0.2548
GSM972234	0.0274	0	0.1199	0	0.0091	0.1466	0	0.0298	0.0079	0	0.058	0	0	0.2073	0.0432	0.1811	0.0235	0	0	0.1241	0.0097	0.0123	0.026	0.176
GSM972233	0	0.0056	0.0685	0	0.0199	0.077	0.0416	0.0075	0	0.0229	0.0691	0	0	0.3422	0.0316	0.1443	0.0095	0	0	0.147	0	0.0134	<0.0001	0.4206
GSM972232	0.0369	0	0.1104	0.0424	0	0.0213	0.0231	0.0428	0.0391	0.0391	0.0621	0	0	0.2458	0.0301	0.1967	0	0.0118	0	0.077	0	0.0214	0.016	0.197
GSM972231	0.0145	0	0.1761	0.0145	0	0.0622	0.0315	0.0323	0	0	0.0331	0	0	0.2087	0.0319	0.0896	0.0069	0.0234	0	0.1564	0	0.1188	<0.0001	0.5506
GSM972230	0	0	0.1362	0.04	0	0.0468	0	0.1011	0.0757	0	0.0753	0	0	0.1525	0.0661	0.138	0.018	0.0487	0	0.0918	0	0.0096	0.018	0.1864
GSM972229	0.017	0	0.0304	0.0129	0	0	0.0046	0.0498	0.0103	0.0448	0	0.0271	0	0.2203	0.0385	0.0705	0.0011	0.002	0	0.3927	0.0211	0.0568	<0.0001	0.3981
GSM972228	0	0	0.2027	0.0793	0	0.1523	0	0.0562	0.045	0	0.0186	0	0	0.0142	0.0484	0.1595	0.0888	0.0155	0	0.1132	0	0.0065	0.006	0.2702
GSM972227	0	0	0.2962	0.0293	0	0.1927	0	0	0.0402	0	0.1031	0	0.0183	0	0.0219	0.1049	0.0563	0.0172	0	0.1198	0	0	<0.0001	0.4216
GSM972226	0	0.0092	0.2042	0.0195	0	0.1733	0	0	0.0066	0	0.096	0	0	0.0768	0.0199	0.1215	0	0.0227	0	0.1909	0.0198	0.0397	0.001	0.3346
GSM972225	0.0186	0	0.1261	0.0893	0	0.0926	0.0759	0.1007	0.0074	0	0.06	0	0.0066	0	0.0567	0.0663	0.064	0.0262	0	0.1954	0.014	0	0.001	0.3242
GSM972224	0.0209	0	0.0752	0.0936	0	0	0.0064	0.0881	0.0378	0.015	0.0137	0	0	0.1392	0.1185	0.1582	0.0126	0.0042	0	0.1424	0	0.0743	<0.0001	0.3992
GSM972223	0	0.0045	0.1946	0	0	0.1436	0.0259	0.0344	0	0	0.0547	0	0	0.1291	0.0681	0.1273	0.0588	0	0	0.0473	0.0007	0.111	<0.0001	0.3915
GSM972222	0	0	0.1658	0.0334	0	0.1364	0	0.0381	0	0	0.0313	0.0056	0	0.0666	0.0552	0.2468	0.0737	0.0215	0	0.1145	0.0037	0.0075	0.303	0.0548
GSM972221	0.022	0	0.1973	0.0281	0.0034	0.1593	0.0153	0.0339	0	0.0249	0.0598	0	0	0.1267	0.0359	0.0775	0.0345	0.0282	0	0.1533	0	0	0.001	0.3255
GSM972220	0.0558	0	0.0968	0	0	0.0225	0.0778	0.097	0.0311	0.0773	0.0469	0	0	0.1248	0.1247	0.102	0.0104	0	0	0.1171	0	0.0159	<0.0001	0.4966
GSM972219	0.0348	0	0.172	0.0674	0	0.0913	0	0.0286	0.0409	0	0.0932	0	0	0.192	0.038	0.0699	0.0023	0.0237	0	0.1458	0	0	0.016	0.2005
GSM972218	0.017	0	0.1659	0.0324	0	0.2548	0	0.0418	0.0248	0	0.083	0	0	0	0.0219	0.1326	0.0886	0.03	0	0.1071	0	0	0.011	0.2181
GSM972217	0.0034	0.0022	0.2022	0.0773	0	0.0287	0	0.0983	0	0	0.0592	0	0	0.1245	0.0681	0.2011	0.0286	0.0171	0	0.0892	0	0	0.003	0.3059
GSM972216	0.0557	0	0.2456	0	0.0439	0.0379	0.096	0.0411	0.0099	0.018	0.0908	0	0	0.1286	0	0.0482	0.0219	0.039	0	0.1054	0	0.0179	0.001	0.3716
GSM972215	0.0141	0	0.086	0.006	0.0034	0	0.0774	0.1352	0	0.0162	0.0564	0	0	0.0961	0.1083	0.2019	0.0112	0.0358	0	0.1067	0.0256	0.0195	0.001	0.3442
GSM972214	0	0.0217	0.14	0	0	0.1706	0	0.0164	0	0.0152	0.0667	0	0	0.0954	0.0081	0.1675	0.0163	0.0495	0	0.1085	0.0914	0.0325	0.01	0.2318
GSM972213	0	0.0876	0.0848	0.0512	0	0	0.0127	0.1045	0.0093	0	0.0375	0.0011	0	0.4318	0.0016	0.102	0.0322	0	0	0.0341	0	0.0096	0.032	0.1678
GSM972212	0.0477	0	0.1737	0	0.053	0.1457	0.0044	0.022	0.0172	0.0388	0.0794	0	0.0008	0.0207	0.0168	0.1161	0.0618	0.0375	0	0.1644	0	0	0.026	0.1763
GSM972211	0	0.0543	0.0767	0.0394	0	0.0571	0	0.0439	0.0794	0	0.0805	0	0	0.2107	0.0289	0.1502	0.035	0.0198	0	0.124	0	0	0.007	0.2661
GSM972210	0.0492	0	0.139	0	0.001	0	0.0574	0.0627	0.0436	0.0034	0.0788	0	0	0.2414	0.0519	0.1201	0.0199	0	0	0.1062	0.0253	0	<0.0001	0.4342
GSM972209	0.0348	0.0104	0.1241	0.0858	0	0	0.0712	0.0804	0	0.0717	0.0272	0	0	0.1196	0.0923	0.2119	0.0038	0	0	0.0403	0	0.0264	<0.0001	0.417
GSM972208	0	0.0112	0.2569	0.0262	0	0.0408	0.054	0.0598	0.0147	0.0584	0.0018	0	0	0.1482	0.142	0.1322	0.003	0	0	0.0337	0	0.0173	<0.0001	0.4824
GSM972207	0.0353	0	0.307	0	0	0.107	0	0.0643	0.0409	0	0.0719	0	0	0.0415	0.0766	0.0717	0.0129	0.0261	0	0.1301	0.0121	0.0026	<0.0001	0.412
GSM972206	0.018	0.0131	0.2102	0.0452	0.0002	0.0526	0.0708	0.0277	0.0146	0	0.1154	0	0	0	0	0.1984	0.0897	0.0328	0	0.1111	0	0	0.004	0.2981
GSM972205	0.0446	0	0.1053	0.1038	0	0.0587	0.1383	0.0836	0	0.0249	0.0475	0	0.0081	0.001	0.1014	0.0979	0.0659	0.0021	0	0.117	0	0	<0.0001	0.4263
GSM972204	0.006	0	0.3498	0	0	0.2152	0.0394	0.0265	0	0.021	0.0653	0	0	0.06	0.0167	0.0741	0.029	0.0093	0	0.0832	0	0.0044	<0.0001	0.5405
GSM972203	0.017	0	0.1966	0.1145	0	0.059	0.0405	0.0754	0.0265	0.0017	0.0593	0	0	0.0417	0.0826	0.1508	0.1021	0.0102	0.003	0.0192	0	0	<0.0001	0.4727
GSM972202	0.0259	0	0.2572	0.0086	0.0083	0.0623	0.0413	0.0349	0.0285	0.0184	0.0592	0	0	0.0559	0.0429	0.1281	0.0425	0.0263	0	0.0847	0.0251	0.0499	<0.0001	0.4105
GSM972201	0.0101	0	0.3773	0.0077	0	0.0087	0	0.0981	0.0124	0.0476	0.0037	0.0009	0	0.1043	0.0612	0.1095	0.0111	0.0094	0	0.1167	0.0198	0.0015	<0.0001	0.5595
GSM972200	0.0369	0	0.269	0.0636	0	0.0273	0.0413	0.0635	0.0375	0	0.0407	0	0.0015	0.0403	0.0455	0.1223	0.0483	0.0288	0	0.1335	0	0	<0.0001	0.4877
GSM972199	0	0.0015	0.2689	0.168	0.0166	0.0126	0.0054	0.0655	0.0415	0	0.0809	0	0.0012	0.0262	0.0415	0.0997	0.0948	0.0016	0.0006	0.0734	0	0	0.001	0.3394
GSM972198	0.0358	0	0.1825	0.0575	0	0.0836	0.0677	0	0.0519	0	0.1237	0	0	0.0261	0	0.1153	0.0507	0.0424	0	0.1461	0.0166	0	0.03	0.1711
GSM972197	0.0306	0	0.3397	0.0788	0	0.0109	0.0199	0.0649	0.0771	0	0.021	0	0	0.1256	0.0143	0.0562	0.0239	0	0	0.125	0	0.012	<0.0001	0.4462
GSM972196	0	0.0271	0.1484	0.1359	0.0243	0	0.0583	0.0834	0	0	0.0751	0	0	0.0843	0.1046	0.0373	0.0106	0	0	0.1433	0	0.0673	0.007	0.259
GSM972195	0.028	0	0.2648	0.0449	0	0.0247	0.0368	0.0756	0.02	0	0.0903	0	0	0.0242	0.0548	0.0913	0.0486	0.0192	0	0.1592	0	0.0176	0.001	0.3374
GSM972194	0.0564	0	0.2176	0.0999	0.0188	0	0.103	0.0664	0.0397	0	0.0709	0	0.0116	0.1136	0	0.0254	0.0439	0	0	0.1219	0.011	0	<0.0001	0.3892
GSM972193	0.047	0	0.2693	0.1561	0	0.0088	0	0.1722	0.0642	0	0.0377	0	0	0.0775	0.0307	0.0322	0.0183	0.0443	0	0.0417	0	0	<0.0001	0.3904
GSM972192	0.0068	0	0.3094	0.0073	0	0.0414	0.0059	0.0716	0.0127	0	0.0591	0	0	0.1113	0.0987	0.0847	0.0007	0.0712	0	0.1017	0	0.0174	<0.0001	0.5385
GSM972191	0	0	0.1725	0	0.0806	0.2009	0.0014	0.0056	0.0085	0	0.1283	0	0.02	0.1059	0.0543	0.054	0.0183	0	0	0.1497	0	0	0.09	0.1195
GSM972190	0.0551	0	0.2095	0.0027	0.0663	0.1069	0.0251	0.0195	0.0372	0	0.1808	0	0	0.1059	0	0.015	0.0123	0	0	0.1357	0	0.028	0.008	0.2481
GSM972189	0	0.0544	0.0523	0.0485	0	0	0.1624	0.0463	0.0289	0.118	0.0458	0	0	0.0681	0.1259	0.1735	0.0389	0.0115	0.0255	0	0	0	<0.0001	0.4828
GSM972188	0	0	0.1885	0	0.08	0.0751	0	0.0213	0.0273	0	0.0656	0	0	0.0009	0.0098	0.2193	0.118	0.0418	0	0.1447	0	0.0075	0.004	0.2928
GSM972187	0.0296	0	0.3317	0	0	0	0	0.075	0.0512	0.0248	0.0339	0	0	0.1693	0.0337	0.1031	0.014	0.0055	0	0.1029	0	0.0253	<0.0001	0.5359
GSM972186	0	0	0.139	0.1021	0	0.0403	0	0.0872	0.037	0.0081	0.034	0	0.0441	0	0.0226	0.32	0.0288	0.0413	0.0149	0.068	0.0126	0	<0.0001	0.4907
GSM972185	0	0	0.2958	0.127	0.0471	0	0	0.0511	0.0388	0	0.0325	0.0077	0.0018	0.0096	0.0217	0.1084	0.1585	0.0023	0	0.0977	0	0	0.004	0.2949
GSM972184	0	0	0.0329	0.0892	0	0.1561	0	0.0478	0	0.0327	0.0234	0.0324	0	0.0981	0.0776	0.1815	0.0951	0	0	0.1061	0	0.0271	<0.0001	0.3821
GSM972183	0.0473	0	0.3246	0	0.1217	0.1461	0	0	0.0379	0	0.0758	0	0.0142	0.0144	0	0.001	0.0443	0.0269	0	0.1397	0	0.006	<0.0001	0.3932
GSM972182	0.0486	0	0.2455	0.0147	0.0611	0.1385	0	0.0043	0.0023	0	0.1647	0	0	0.0445	0	0	0.0435	0.026	0	0.1967	0	0.0096	0.017	0.1935
GSM972181	0	0	0.0325	0.0063	0.0071	0.0508	0	0	0.0005	0	0.0578	0	0.0829	0.2814	0.0226	0.1136	0	0.0081	0	0.2603	0	0.0759	<0.0001	0.5748
GSM972180	0	0.0167	0.0532	0.338	0	0	0.1548	0.075	0	0.0294	0.0362	0.0057	0.006	0	0.0754	0.0903	0.0185	0.0152	0	0.0857	0	0	<0.0001	0.505
GSM972179	0.0887	0	0.2848	0.0652	0.0009	0	0.0088	0.0468	0	0.0089	0.0216	0	0	0.1805	0.0583	0.089	0.001	0	0	0.1356	0	0.0099	<0.0001	0.5213
GSM972178	0	0.0015	0.2932	0.1507	0	0.0657	0	0.0474	0.0518	0	0.0763	0	0.0012	0	0.0212	0.1022	0.0904	0.0101	0	0.0869	0.0014	0	0.001	0.3643
GSM972177	0.0202	0	0.2579	0.0681	0	0.0061	0.0025	0.0235	0.0939	0	0.097	0	0	0.1452	0.0263	0.1169	0.0412	0.0003	0	0.1009	0	0	0.001	0.3705
GSM972176	0.0384	0	0.2222	0.0758	0.0265	0	0.0164	0.081	0.0478	0	0.0867	0	0	0.0966	0.1531	0.0509	0.045	0	0	0.0595	0	0	<0.0001	0.4869
GSM972175	0.0208	0	0.3221	0.1549	0.0018	0	0.0497	0.0246	0.0404	0	0.0676	0	0	0.0039	0.0385	0.0372	0.1217	0.0116	0	0.0956	0	0.0095	<0.0001	0.4836
GSM972174	0	0.0098	0.3106	0.1617	0	0	0	0.1584	0.0228	0.0023	0.0453	0	0	0.0511	0.0405	0.068	0.0018	0.0197	0	0.1079	0	0	<0.0001	0.4184
GSM972173	0	0	0.2362	0.0797	0.0477	0.0538	0.1088	0	0	0	0.0658	0	0	0.0666	0.0393	0.0983	0.0826	0.0155	0	0.0688	0	0.0369	0.007	0.2559
GSM972172	0	0.0226	0.1912	0.0786	0.0315	0	0	0.0696	0.071	0	0.0555	0	0	0.0109	0.0273	0.1069	0.0712	0.0796	0	0.184	0	0	0.008	0.2465
GSM972171	0.0512	0	0.1174	0.0399	0.0236	0.0209	0.1574	0.0496	0	0.0572	0	0.0111	0	0.0236	0.1774	0.1631	0.0143	0.0453	0	0.0264	0	0.0216	<0.0001	0.5078
GSM972170	0.0593	0	0.2636	0	0.0872	0.0011	0.0717	0.0778	0	0	0.0507	0	0	0.0927	0.0509	0.077	0.0248	0.0076	0	0.1357	0	0	<0.0001	0.4385
GSM972169	0.024	0	0.1458	0	0.0137	0.166	0.0709	0.0526	0	0	0.0983	0	0	0.0302	0.0313	0.0749	0.0318	0.0548	0	0.1999	0	0.0059	0.065	0.1331
GSM972168	0	0.0036	0.0804	0	0	0.1113	0	0.0338	0.0225	0.0512	0.0223	0	0	0.1211	0.092	0.1241	0	0.0197	0	0.2033	0	0.1146	<0.0001	0.3853
GSM972167	0.0087	0	0.3332	0	0	0.2033	0.0098	0.0299	0.0193	0	0.0524	0	0	0.0932	0.0309	0.0787	0.0574	0.0137	0	0.0382	0.0144	0.0168	0.004	0.2967
GSM972166	0	0	0.2221	0.0197	0	0.17	0	0.034	0	0	0.0601	0	0	0.0658	0.0096	0.1246	0.0435	0.0148	0	0.1892	0.0133	0.0333	0.008	0.2393
GSM972165	0.0351	0	0.264	0.0129	0	0.075	0	0.0551	0.0148	0	0.0598	0	0	0.0764	0.0441	0.1754	0.0258	0.0148	0	0.0986	0.0261	0.022	<0.0001	0.4284
GSM972164	0.0339	0	0.2252	0.0307	0.0188	0.0372	0.0462	0.0199	0	0	0.0443	0	0	0.2158	0.0534	0.0845	0.0124	0.0064	0	0.1114	0	0.0601	0.001	0.3668
GSM972163	0	0.0097	0.3971	0.0177	0	0.166	0	0.0391	0.0136	0	0.0753	0	0	0.0456	0.0316	0.0985	0.0119	0.0155	0	0.0687	0	0.0098	<0.0001	0.5287
GSM972162	0	0.0111	0.0552	0	0	0.0577	0.0117	0.0902	0.0295	0	0.0549	0	0	0.2142	0.1505	0.1302	0.0134	0.0088	0	0.1374	0	0.0351	0.007	0.2606
GSM972161	0	0	0.2016	0.0749	0.0177	0.127	0	0.0038	0	0	0.1066	0	0	0.0312	0.0117	0.1621	0.093	0.0363	0	0.1261	0	0.008	0.181	0.0871
GSM972160	0	0	0.185	0.0206	0	0.0779	0	0.0135	0.117	0	0.063	0.047	0	0.0608	0.0693	0.1628	0.0901	0.0081	0.0819	0	0.0029	0	0.001	0.3201
GSM972159	0	0	0.2948	0	0	0.1454	0	0.1025	0.0076	0	0.0555	0	0	0.1089	0.072	0.1176	0	0.0176	0	0.0678	0	0.0102	0.001	0.3675
GSM972158	0.0198	0	0.2402	0.0313	0	0.0416	0.0184	0.0758	0	0.0404	0.0039	0	0	0.1665	0.0739	0.1168	0.0098	0.0103	0	0.118	0	0.0333	<0.0001	0.4312
GSM972157	0	0.0022	0.2701	0.0067	0.0168	0.0526	0.0454	0.0729	0	0.0122	0.0527	0	0	0.0238	0.0677	0.1483	0.0373	0.0326	0.0157	0.0936	0.0304	0.0191	0.011	0.2168

CIBERSORT scores: 0.4, 0.3, 0.2, 0.1, 0.0

Supplementary Figure S1. Continued

Input Samples	B cells naive	B cells memory	Plasma cells	T cells CD8	T cells CD4 naive	T cells CD4 memory resting	T cells CD4 memory activated	T cells follicular helper	T cells regulatory (Tregs)	T cells gamma delta	NK cells resting	NK cells activated	Monocytes	Macrophages M0	Macrophages M1	Macrophages M2	Dendritic cells resting	Dendritic cells activated	Mast cells resting	Mast cells activated	Eosinophils	Neutrophils	P-value	Pearson correlation
GSM972356	0.0082	0	0.2623	0.0177	0	0.1384	0.0649	0.094	0.0037	0.0002	0.0689	0	0	0.0602	0.0476	0.087	0.031	0.0155	0	0.0612	0.0081	0.0311	0.001	0.3741
GSM972355	0	0.0163	0.12	0.1339	0	0.0769	0.0047	0.0665	0	0	0.0476	0	0	0.0742	0.1158	0.2175	0.0318	0.0081	0.0124	0.0465	0.0046	0.023	0.016	0.2
GSM972354	0	0.057	0.0679	0.1715	0	0	0.0807	0.1304	0	0	0.0138	0.0605	0.018	0.076	0.1358	0.0306	0.0237	0.0242	0	0.1099	0	0	<0.0001	0.3875
GSM972353	0.0249	0	0.2513	0.0328	0	0.0065	0.0702	0.0899	0.0004	0.0067	0.0297	0.0066	0	0.0781	0.1749	0.1109	0.0372	0.0456	0.0002	0.0261	0	0.008	<0.0001	0.4992
GSM972352	0	0.03	0.1428	0	0	0.1087	0	0.0429	0.0356	0	0.0075	0.0026	0	0.0165	0.0616	0.3416	0.0663	0.0146	0	0.0825	0.0179	0.0288	<0.0001	0.3898
GSM972351	0	0	0.4112	0.0112	0.0396	0.0663	0.0086	0.0312	0.0231	0.0102	0.068	0	0	0.0843	0	0.1181	0.0214	0.0198	0	0.0757	0	0.0115	<0.0001	0.4546
GSM972350	0	0.0641	0.2718	0.0056	0	0.1083	0	0.0679	0.0779	0.0155	0.0548	0	0.0216	0.0839	0.0149	0.0139	0.0162	0.0252	0	0.1585	0	0	0.015	0.2011
GSM972349	0.0141	0	0.4627	0.0647	0.0164	0.0042	0	0.0562	0	0	0.0093	0.005	0	0.1502	0.0552	0.0981	0	0	0.0127	0.038	0.0039	0.0094	<0.0001	0.6641
GSM972348	0	0.0329	0.213	0.0161	0	0.1773	0.09	0.0427	0	0	0.1191	0	0.0019	0.0197	0.0113	0.0847	0.0329	0.0828	0	0.0695	0.0061	0	0.008	0.2441
GSM972347	0.0241	0	0.3032	0.1052	0	0	0.0385	0.0731	0.0046	0.0203	0.0036	0.0199	0	0.0748	0.1122	0.1206	0.0211	0.0383	0.0054	0.0276	0	0.0077	<0.0001	0.4187
GSM972346	0	0.0291	0.1603	0.0834	0	0.0924	0	0.0407	0.0466	0	0.1208	0	0	0.0459	0.0149	0.0748	0.007	0.0481	0	0.1926	0	0.0436	0.025	0.179
GSM972345	0	0.0057	0.0734	0.0401	0	0.0078	0	0.0707	0.0635	0	0.0454	0	0	0.1024	0.1475	0.3296	0.0377	0.0089	0.0021	0.0605	0	0.0047	<0.0001	0.3981
GSM972344	0	0.0088	0.1152	0	0	0.1461	0.0453	0.0392	0.0158	0	0.1176	0	0	0.13	0.0285	0.1472	0.0339	0.0265	0	0.1457	0	0	0.031	0.1695
GSM972343	0.0334	0	0.1091	0.0413	0	0.1381	0.0305	0.0573	0.0518	0	0.0508	0	0.0064	0.0458	0.0674	0.1493	0.133	0	0	0.0845	0	0.0015	0.001	0.3128
GSM972342	0	0	0.2791	0.0435	0	0.0306	0.005	0.0829	0.012	0.015	0.0571	0	0	0.2123	0.0556	0.1233	0.005	0.0183	0	0.0603	0	0	<0.0001	0.3988
GSM972341	0.0334	0	0.1279	0.1255	0.0175	0	0.0209	0.0644	0	0	0.039	0.054	0	0.1932	0.0522	0.1615	0.0308	0.0163	0	0.0559	0	0.0077	0.002	0.3101
GSM972340	0.0039	0.0002	0.0361	0	0.049	0.0408	0.0364	0.0137	0.0142	0	0.0927	0	0	0.2924	0	0.066	0	0.0116	0	0.2956	0.0067	0.0408	0.001	0.321
GSM972339	0	0.018	0.344	0.0084	0	0.1245	0.0062	0.0401	0.0141	0	0.0681	0	0.0009	0.0149	0.0675	0.1665	0.0529	0.0241	0	0.0462	0	0.0038	<0.0001	0.4198
GSM972338	0.0062	0	0.2875	0.0435	0.0704	0.1171	0.0617	0.0048	0	0	0.0757	0	0.0236	0.0224	0.0261	0.0914	0.0422	0.0248	0	0.1026	0	0	0.001	0.3118
GSM972337	0.058	0	0.1259	0.1377	0	0	0.1339	0.0727	0	0.0799	0.0175	0	0	0.0459	0.1464	0.1037	0.0168	0	0.0061	0.0461	0	0.0095	<0.0001	0.5449
GSM972336	0.003	0	0.2416	0.0255	0	0.1106	0	0.0465	0.0295	0	0.043	0	0	0.101	0.0175	0.1716	0.0885	0.023	0.0419	0.0373	0.0143	0.0053	0.001	0.3693
GSM972335	0.0337	0	0.3047	0	0	0.1025	0.0573	0.0411	0.001	0	0.0591	0	0	0.0472	0.0262	0.1289	0.0218	0.0249	0	0.1324	0	0.0191	0.001	0.3446
GSM972334	0.0074	0	0.0942	0.0767	0.0038	0	0.093	0.0848	0	0.0068	0.0585	0	0	0.0734	0.1568	0.1694	0.0132	0.0113	0	0.1319	0	0.0187	<0.0001	0.5264
GSM972333	0.0349	0	0.3083	0	0.0581	0.1192	0	0.005	0.0413	0	0.0527	0	0	0.0994	0.0265	0.1229	0.058	0.0143	0	0.034	0.0164	0.0091	<0.0001	0.4135
GSM972332	0.0035	0	0.1427	0.0806	0	0	0	0.1161	0.0301	0	0.0517	0.0131	0	0.0939	0.0381	0.1557	0.0458	0.0256	0	0.1422	0	0.0609	0.005	0.2752
GSM972331	0.0344	0	0.1363	0	0.0262	0.1401	0.0685	0.0206	0.0028	0	0.1053	0	0	0.1821	0	0.0883	0	0.0311	0	0.1535	0	0.0108	0.017	0.1897
GSM972330	0	0	0.1723	0	0.0021	0.0609	0.0358	0.0631	0	0.0336	0.0393	0	0	0.1595	0.1429	0.0757	0	0	0	0.1865	0	0.0283	<0.0001	0.3794
GSM972329	0.0132	0	0.24	0.0473	0	0.0551	0	0.119	0.0302	0.0147	0.0193	0.01	0	0.0344	0.0875	0.114	0.1251	0	0	0.0529	0.0075	0.0299	<0.0001	0.4933
GSM972328	0	0	0.1218	0	0.0393	0.0655	0	0.0613	0.0261	0	0.1024	0	0	0.2131	0.0184	0.0951	0.0348	0.0496	0	0.1465	0.0034	0.0227	0.014	0.2058
GSM972327	0	0	0.1862	0.0742	0	0.0931	0.0357	0.0248	0.0063	0.0045	0.0203	0	0.0071	0.0798	0.0708	0.163	0.0869	0.0021	0	0.0776	0.0429	0.0247	<0.0001	0.4959
GSM972326	0.0414	0	0.1269	0	0	0.23	0	0.0157	0.106	0.0192	0.1003	0	0	0.0561	0.008	0.0985	0.0152	0.0456	0	0.1372	0	0	0.01	0.2315
GSM972325	0	0.0327	0.1513	0.0832	0	0.0344	0.0157	0.029	0.0281	0.0183	0.0047	0	0	0.1752	0.0277	0.0551	0.0293	0.0264	0	0.2314	0	0.0574	0.004	0.2932
GSM972324	0	0.036	0.0619	0	0.0015	0.0661	0.1948	0.0476	0	0.0687	0.1076	0.0049	0.0226	0.1618	0.0482	0.0648	0.0819	0	0.0034	0.0283	0	0	0.018	0.1883
GSM972323	0.0094	0.0058	0.306	0	0.0461	0	0.055	0.0515	0.025	0	0.1185	0	0	0.124	0.0307	0.0847	0.0409	0.0041	0	0.0984	0	0	<0.0001	0.4661
GSM972322	0.0724	0	0.2612	0.0069	0	0.1432	0.0281	0.0667	0.0232	0.0072	0.0893	0	0	0.027	0.0708	0.0754	0.0303	0.0055	0	0.0841	0.0014	0.0074	<0.0001	0.529
GSM972321	0.0257	0.0181	0.3534	0.0714	0.0021	0	0	0.0764	0.0179	0.0313	0.0454	0	0.0001	0.0327	0.0393	0.1277	0.0429	0.0227	0.0163	0.0352	0.0066	0.0348	<0.0001	0.5357
GSM972320	0.0585	0	0.2177	0.0104	0	0.1188	0.0115	0.0358	0.0246	0	0.1039	0	0	0.0329	0.0474	0.0496	0.1134	0	0	0.1641	0.0114	0	0.008	0.2429
GSM972319	0	0.0187	0.1506	0.1671	0	0	0.042	0.0591	0.0342	0	0.0755	0	0	0.0153	0.129	0.1246	0.0718	0.0314	0.015	0.0656	0	0	0.011	0.2139
GSM972318	0	0	0.2703	0.0423	0.0349	0.0265	0.0364	0.033	0.0203	0.0145	0.082	0	0	0.0013	0.0189	0.1532	0.0174	0.0231	0	0.1889	0.0085	0.0286	<0.0001	0.4272
GSM972317	0	0.0711	0.0586	0.1457	0	0	0.0425	0.0708	0.027	0	0.0311	0.0333	0	0.0313	0.1819	0.1617	0.0537	0	0	0.0823	0	0.0088	<0.0001	0.4218
GSM972316	0.0273	0	0.2226	0	0	0.1129	0	0.0475	0.022	0	0.0138	0.0249	0	0.1159	0.0353	0.1355	0.0446	0.0165	0	0.134	0.0002	0.047	0.001	0.3317
GSM972315	0.0394	0	0.2219	0.0658	0	0.1574	0	0.0115	0.0143	0	0.0663	0	0	0	0.0498	0.1766	0.0622	0.0285	0	0.1028	0	0.0036	0.004	0.2982
GSM972314	0.0114	0	0.0623	0.1543	0	0	0.0584	0.1041	0.0266	0	0	0.0719	0	0.0894	0.1447	0.1809	0.0503	0	0	0.0269	0	0.0188	0.001	0.3653
GSM972313	0	0	0.1051	0	0.0176	0.2699	0.1861	0.0177	0	0	0.0966	0.0168	0.0297	0.0444	0.0595	0.0105	0.088	0.0457	0.0122	0	0	0	0.074	0.1282
GSM972312	0.0312	0.0073	0.1315	0.0906	0	0.0673	0.0468	0.0352	0	0	0.1103	0	0	0.1414	0.0197	0.0731	0.0212	0.0324	0	0.15	0	0.042	0.013	0.2066
GSM972311	0	0.0512	0.1394	0.1121	0	0.029	0	0.0677	0.0599	0	0	0.0282	0	0	0.0639	0.3722	0.0216	0	0.0322	0	0	0.0228	0.45	0.0332
GSM972310	0.0439	0	0.1647	0	0	0.2053	0.01	0.0575	0.0003	0	0.13	0	0	0.1068	0.072	0.0347	0.009	0.0405	0	0.1209	0	0.0043	0.001	0.3388
GSM972309	0	0.0976	0.1806	0.0757	0.0368	0	0.1399	0.0909	0	0	0.078	0	0.0534	0.0557	0.0129	0.0029	0.0373	0.0754	0	0.063	0	0	0.131	0.1017
GSM972308	0.0439	0	0.1553	0.1346	0	0	0.0151	0.0954	0	0.0561	0	0.0862	0	0.084	0.0991	0.1582	0.0282	0.0148	0	0.0291	0	0	<0.0001	0.5197
GSM972307	0	0.0496	0.1163	0.1053	0	0.0599	0.0897	0.082	0	0.0182	0.1033	0	0	0.0122	0.0901	0.0137	0.0602	0.0434	0	0.1349	0	0.0213	0.034	0.1666
GSM972306	0.0171	0	0.1988	0.0158	0.0663	0.0699	0.0403	0.0259	0	0	0.1055	0	0	0.1557	0.0005	0.0298	0.0298	0.0655	0	0.1463	0	0.033	0.026	0.1765
GSM972305	0.0565	0	0.1329	0.0712	0	0	0.0145	0.0557	0.0173	0.025	0.0416	0	0	0.1741	0.0235	0.1211	0	0.0403	0	0.2104	0.0066	0.0093	<0.0001	0.4492
GSM972304	0.0159	0	0.2585	0.0187	0.0125	0.1501	0.0626	0.0204	0.0149	0	0.079	0	0	0.1429	0.0505	0.0178	0.0049	0.0314	0	0.1151	0	0.0049	0.001	0.3742
GSM972303	0	0.0444	0.1249	0.0552	0	0.1412	0.0755	0.0465	0	0	0.1019	0	0.0048	0.1008	0.0454	0.0583	0	0.0653	0	0.1359	0	0	0.249	0.0681
GSM972302	0.0709	0	0.222	0	0.015	0.1175	0.0147	0.0495	0	0	0.0769	0	0.02	0.0336	0.0245	0.0993	0.0656	0.0333	0	0.1571	0	0	0.004	0.2924
GSM972301	0.023	0	0.3013	0.0263	0	0.0819	0.0247	0.0292	0.0136	0.0303	0.0334	0	0	0.1027	0.0651	0.1263	0.0365	0.0091	0	0.0564	0.0146	0.0255	<0.0001	0.5176
GSM972300	0.0099	0	0.2325	0.0149	0	0.2844	0.0045	0	0.0014	0.0133	0.0648	0	0	0.0012	0.0347	0.0979	0.1412	0.008	0	0.0853	0.0062	0	0.001	0.3704
GSM972299	0.0592	0	0.1297	0	0.0074	0.1577	0.1157	0.0231	0	0	0.1019	0	0	0.0914	0.1247	0.0861	0.0466	0.0065	0	0.05	0	0	0.001	0.358
GSM972298	0	0.0186	0.2037	0.0335	0	0.0213	0.0408	0.0968	0.0084	0	0.0545	0	0	0.1551	0.0811	0.1487	0.0074	0.024	0	0.0829	0	0.0234	0.001	0.3422
GSM972297	0.0166	0	0.1973	0.0387	0.0077	0	0	0.0713	0.015	0	0.0162	0.0189	0	0.1279	0.0794	0.1318	0	0	0	0.1652	0	0.1139	0.001	0.3776
GSM972296	0.0599	0	0.3579	0.0625	0	0.0773	0.0081	0.0732	0.0148	0	0.0897	0	0	0.0714	0.0253	0.0259	0	0.0336	0	0.1003	0	0	<0.0001	0.4652
GSM972295	0	0.0022	0.2154	0.1326	0	0	0.0107	0.1173	0.0662	0	0.0514	0	0.0197	0.0801	0.0074	0.0977	0.0557	0.0655	0	0.078	0	0	0.007	0.2627
GSM972294	0	0.0107	0.0352	0.2471	0	0	0.1528	0.0811	0	0.0922	0	0.0751	0	0.0036	0.0972	0.0732	0.0153	0.02	0	0.0821	0.0033	0.0113	<0.0001	0.4178
GSM972293	0.0309	0	0.2486	0.0429	0	0.0646	0.0003	0.1073	0.0066	0.0303	0.0415	0	0	0.026	0.0989	0.1233	0.015	0.01	0	0.1455	0	0.0083	<0.0001	0.4348
GSM972292	0.0114	0	0.0753	0.0481	0	0.0738	0.0243	0.0519	0.0343	0	0.05	0.002	0	0.2465	0.0273	0.0457	0	0.0422	0	0.2259	0	0.0413	0.004	0.2985
GSM972291	0	0	0.1785	0.0219	0	0.0542	0.0479	0.067	0.0115	0.0872	0.0398	0	0	0.064	0.0453	0.1475	0.122	0.0155	0	0.0738	0.0088	0.0153	0.001	0.3338
GSM972290	0.0232	0	0.2316	0.053	0	0.0038	0.0328	0.0725	0	0.001	0.0648	0	0	0.1372	0.098	0.0875	0.0067	0.0236	0	0.1168	0.0208	0.0268	<0.0001	0.5081
GSM972289	0.045	0	0.2744	0	0.0622	0.1422	0	0.0251	0.0688	0	0.0941	0	0.0124	0.0184	0.0015	0.0621	0.0149	0.0384	0	0.1356	0	0.0049	0.001	0.3319
GSM972288	0.0516	0	0.1669	0	0	0.1589	0.0131	0.0306	0.0227	0	0.1165	0	0	0.1549	0.0287	0.1223	0	0	0	0.1088	0	0.0249	0.012	0.2099
GSM972287	0.0081	0	0.0225	0.0041	0.0274	0	0	0.0486	0.0099	0.0277	0	0.0326	0.005	0.0623	0.0388	0.0718	0.0125	0.0299	0	0.5139	0.0011	0.0838	<0.0001	0.4867
GSM972286	0.0296	0	0.1174	0.068	0	0.056	0.0592	0.0994	0.0144	0	0.0709	0	0	0.1593	0.0994	0.1278	0.0206	0.0087	0	0.0639	0	0.0054	0.011	0.219
GSM972285	0.0415	0	0.1667	0	0.0428	0.0446	0	0.026	0.0135	0	0.0707	0	0	0.1384	0.0187	0.085	0.0054	0.0274	0	0.2551	0.0213	0.0429	0.096	0.1154
GSM972284	0.074	0	0.1466	0.0183	0.0442	0.0943	0.1043	0.0548	0	0	0.1146	0	0.0144	0.0618	0.0031	0.099	0.0053	0.0735	0	0.0815	0	0.0102	0.017	0.1915
GSM972283	0.001	0	0.1614	0.0697	0	0	0.0425	0.0564	0.0173	0	0.1333	0	0	0.1853	0.1331	0.0848	0	0.0164	0	0.0974	0	0.0015	<0.0001	0.4943
GSM972282	0.0373	0	0.178	0.0227	0.066	0.0135	0.0377	0.0291	0	0.0132	0.0546	0	0	0.2054	0.0449	0.1066	0	0.0288	0	0.1509	0	0.0113	0.001	0.3433
GSM972281	0.032	0	0.2661	0	0	0.237	0.0933	0	0	0.0191	0.0343	0	0	0.1477	0.0476	0.0483	0	0.0135	0	0.0611	0	0	0.001	0.3425
GSM972280	0.0217	0	0.0886	0	0	0	0.0053	0.0821	0.0289	0.0247	0.0384	0	0	0.2764	0.0419	0.0874	0	0	0	0.2536	0.0003	0.0508	<0.0001	0.4519
GSM972279	0.0392	0	0.2724	0.0191	0	0.0042	0.0327	0.066	0.0092	0.0232	0.0306	0	0	0.1149	0.0355	0.1486	0	0.0388	0	0.1096	0.0083	0.0476	<0.0001	0.5382
GSM972278	0	0.0818	0.1189	0	0	0.0351	0.0326	0.0728	0.0249	0.0936	0.0945	0	0	0.1095	0.1533	0.0897	0.013	0.0439	0	0.0306	0	0.0058	0.004	0.286
GSM972277	0.0036	0	0.1285	0.044	0	0.0535	0.018	0.0635	0.0336	0	0.0651	0	0	0.1484	0.0758	0.1059	0	0.0366	0	0.1876	0	0.0359	0.01	0.2254
GSM972276	0	0.0467	0.1537	0.0832	0	0.0022	0.0905	0.0896	0	0	0.0944	0	0	0.0552	0.1335	0.0654	0.0105	0.0507	0	0.086	0.0209	0.0175	0.001	0.3405
GSM972275	0.0262	0	0.1197	0.1648	0	0	0.0978	0.0478	0	0.0044	0.0112	0.0309	0.001	0.0501	0.1003	0.2132	0.0208	0.0055	0	0.0411	0.0229	0.0421	<0.0001	0.4505
GSM972274	0.0041	0	0.1891	0.1215	0	0	0.0152	0.0457	0.0038	0.026	0.0933	0	0	0.1248	0.1323	0.116	0.0075	0.0081	0	0.1038	0	0.0087	<0.0001	0.5353
GSM972273	0.0022	0	0.3279	0.041	0	0.2038	0	0.0328	0.0158	0	0.0686	0	0	0	0.0185	0.1481	0.0444	0.0273	0	0.0695	0	0	<0.0001	0.4584
GSM972272	0.0676	0	0.1689	0.0796	0.0073	0.0419	0	0.0908	0.0334	0	0.1552	0	0	0	0.0181	0.1118	0.0228	0.0504	0	0.1424	0	0.0097	0.042	0.1565
GSM972271	0.0057	0	0.1006	0.2114	0	0	0.0832	0.0372	0.0044	0	0.1074	0	0.0152	0.0849	0.0643	0.1193	0.0707	0.0275	0	0.0596	0	0.0086	0.036	0.1632
GSM972270	0.0325	0	0.3288	0	0	0	0.0603	0.0723	0	0.0663	0.0003	0.0277	0	0.0442	0.0358	0.1225	0.0035	0.0274	0	0.1545	0	0.0241	<0.0001	0.4839
GSM972269	0.0078	0	0.3216	0.0182	0	0.1691	0.0059	0	0.0184	0	0.085	0	0	0.1492	0.0152	0.0941	0	0.0174	0	0.0982	0	0	<0.0001	0.5245
GSM972268	0.0112	0	0.2479	0.0982	0	0.1313	0	0.0166	0.0195	0	0.0919	0	0	0	0.0025	0.1524	0.0756	0.0193	0	0.1253	0.0021	0.0062	0.008	0.2424
GSM972267	0	0	0.3127	0.1186	0	0	0	0.0359	0.0253	0	0.0574	0	0	0	0.0378	0.1492	0.1196	0.0119	0	0.1307	0	0.001	0.005	0.281
GSM972266	0	0.1033	0.1814	0.0202	0.0115	0.0532	0	0.1282	0	0	0.1314	0	0.049	0.0396	0.0409	0.0421	0.0071	0.052	0	0.114	0.0259	0	0.017	0.191
GSM972265	0.0836	0	0.1518	0.0752	0	0	0	0.0739	0.0383	0	0.023	0	0	0.0232	0.0341	0.2556	0.0809	0.0424	0	0.1084	0	0.0097	0.001	0.3259
GSM972264	0	0.0002	0.0543	0.0072	0	0.0231	0.0569	0.0756	0	0.0084	0.0712	0	0	0.1226	0.1611	0.1876	0.0147	0.0111	0	0.1749	0	0.031	0.001	0.3751
GSM972263	0.021	0	0.232	0	0.0256	0	0.0582	0.0663	0.0385	0.0347	0.0563	0	0	0.0679	0.0676	0.1132	0.0002	0.0656	0	0.1268	0	0.026	<0.0001	0.3886
GSM972262	0	0.0485	0.0473	0.0848	0	0.0283	0	0.0945	0	0.0413	0	0	0	0.03	0.133	0.3213	0.0684	0	0.0278	0.0339	0	0.041	<0.0001	0.5099
GSM972261	0.0138	0	0.2538	0	0	0.1296	0.0428	0.0266	0	0.0256	0.041	0	0	0.0651	0.0934	0.0746	0.0138	0.0164	0	0.1655	0	0.038	<0.0001	0.3883
GSM972260	0.004	0	0.1498	0.0332	0	0.1662	0	0.0191	0.0345	0	0.1321	0	0	0.1101	0.0158	0.0674	0.067	0.0372	0	0.1636	0	0	0.053	0.1411
GSM972259	0.0117	0	0.3188	0.0835	0	0.0461	0	0.056	0.0751	0.0077	0.0263	0	0	0.0276	0.059	0.1853	0.0199	0.0131	0	0.0634	0	0.0064	<0.0001	0.606
GSM972258	0.0303	0	0.0811	0	0.1142	0	0.1489	0.0367	0.0086	0	0.1113	0	0	0.0722	0.0353	0.1149	0.0426	0.0061	0	0.1709	0	0.0267	0.029	0.1726
GSM972257	0	0.0158	0.2174	0	0.0267	0.0577	0.1021	0.03	0	0.0539	0.0721	0	0	0.0946	0.0675	0.1002	0.0253	0	0	0.1262	0	0.0104	0.001	0.3251

CIBERSORT scores: 0.5, 0.4, 0.3, 0.2, 0.1, 0.0

Supplementary Figure S1. Continued

Input Samples	B cells naive	B cells memory	Plasma cells	T cells CD8	T cells CD4 naive	T cells CD4 memory resting	T cells CD4 memory activated	T cells follicular helper	T cells regulatory (Tregs)	T cells gamma delta	NK cells resting	NK cells activated	Monocytes	Macrophages M0	Macrophages M1	Macrophages M2	Dendritic cells resting	Dendritic cells activated	Mast cells resting	Mast cells activated	Eosinophils	Neutrophils	P-value	Pearson correlation
GSM972458	0.0181	0	0.0572	0.0793	0	0.0253	0.1312	0.088	0.0307	0	0.1165	0	0	0.1144	0.1024	0.0941	0.0099	0.0014	0	0.1127	0	0.019	<0.0001	0.4174
GSM972457	0.0363	0	0.2641	0.0371	0	0.1016	0	0.0822	0.0485	0.0074	0.0947	0	0.0156	0.0453	0.0279	0.0314	0.0156	0.037	0	0.1352	0.0201	0	0.005	0.2759
GSM972456	0	0	0.2293	0.0997	0	0.0766	0	0.0338	0.0227	0	0.0603	0	0	0.0469	0.0638	0.2252	0.0636	0.0042	0	0.0694	0	0.0045	0.001	0.3403
GSM972455	0.0183	0	0.0451	0	0.0013	0.0846	0	0.0501	0	0	0.0453	0	0.0223	0.1493	0.0634	0.1007	0	0.015	0	0.2856	0	0.119	<0.0001	0.5278
GSM972454	0.0502	0	0.2815	0.0476	0	0.0755	0.0594	0.0783	0	0	0.0545	0	0	0.0853	0.0748	0.063	0.0186	0	0	0.0874	0.0238	0	<0.0001	0.5691
GSM972453	0	0.0663	0.0561	0	0.0439	0.2422	0	0	0.017	0	0.0908	0	0	0.2197	0.0245	0.0481	0.0223	0.0087	0	0.1604	0	0	0.052	0.1447
GSM972452	0.0326	0	0.1224	0.1303	0	0	0.121	0.0507	0	0.0043	0.0647	0	0	0.1413	0.1072	0.1564	0	0.0026	0	0.0582	0	0.0082	<0.0001	0.4903
GSM972451	0	0.0335	0.1058	0.0324	0	0.2395	0.0106	0	0	0	0.0832	0	0	0.0788	0.0329	0.1002	0.1019	0.012	0	0.1254	0	0.0437	0.088	0.1218
GSM972450	0.0563	0	0.1883	0.0341	0.0023	0.1552	0.0104	0.043	0.0201	0	0.0888	0	0	0.0682	0.0289	0.0558	0.0241	0.041	0	0.1834	0	0	0.006	0.2706
GSM972449	0.059	0	0.1714	0	0.0654	0.1132	0	0.0574	0.0666	0	0.0563	0	0.0114	0.0211	0.0137	0.1354	0.0454	0.0409	0	0.1431	0	0	0.001	0.3272
GSM972447	0.0371	0	0.1816	0	0	0.1149	0	0.0281	0.0243	0.0317	0.0817	0	0	0.0509	0.024	0.1518	0.0112	0.0288	0	0.1746	0.0435	0.0155	0.001	0.3452
GSM972445	0.0141	0	0.0543	0.1928	0	0	0.1157	0.0732	0.0262	0	0.0256	0.0091	0	0.025	0.1157	0.0941	0.0093	0.0932	0	0.1258	0.0052	0.0206	0.001	0.3755
GSM972444	0.0201	0	0.214	0	0.0344	0.0191	0.0373	0.0617	0	0.0196	0.0598	0	0	0.1732	0.1028	0.1088	0	0.0199	0	0.0974	0	0.032	<0.0001	0.4944
GSM972443	0.0634	0	0.0755	0	0.0115	0.0394	0.007	0.0567	0.0244	0.051	0.081	0	0	0.2924	0.0419	0.0965	0	0.0238	0	0.1356	0	0	0.017	0.1913
GSM972442	0.1213	0.0107	0.1269	0.1443	0	0.1074	0.0545	0.0199	0.009	0.0186	0.0369	0	0	0.0393	0.1626	0.1138	0.0252	0	0.0045	0.005	0	0	<0.0001	0.5796
GSM972441	0.0252	0	0.1624	0.0105	0	0.1304	0.0376	0.0453	0.0095	0.0603	0.0496	0	0	0.0445	0.0424	0.1743	0.0591	0.0262	0	0.0868	0.0232	0.0128	<0.0001	0.5177
GSM972440	0.0065	0	0.2604	0.0254	0	0.0409	0	0.0673	0.0058	0.0312	0	0.0261	0	0.0861	0.1319	0.1287	0	0.0012	0	0.1567	0	0.0317	<0.0001	0.396
GSM972439	0.0524	0	0.4072	0	0.0149	0.0859	0.0976	0.0174	0	0	0.0445	0	0.0307	0	0	0.1182	0.0155	0.0318	0.0634	0.0205	0	0	<0.0001	0.3809
GSM972438	0.0361	0	0.0923	0	0	0.1052	0	0.0706	0.0261	0.0171	0.0244	0.008	0.0403	0.1728	0.0897	0.1195	0	0	0	0.0882	0	0.1096	0.001	0.3776
GSM972437	0.018	0	0.3184	0.0291	0	0.029	0.0108	0.0469	0.0117	0.0024	0.0352	0	0	0.1605	0.0538	0.177	0.007	0	0	0.0698	0	0.0306	<0.0001	0.4364
GSM972436	0	0.0629	0.0333	0.0109	0.0199	0.0614	0	0	0.0119	0	0.0557	0	0	0.292	0.0011	0.239	0.0386	0	0	0.1432	0	0.0301	0.003	0.3084
GSM972435	0	0	0.268	0.0037	0	0.1771	0.055	0	0	0	0.1176	0	0.0116	0.0243	0	0.1563	0.0309	0.0443	0	0.1059	0	0.0052	0.001	0.3689
GSM972434	0.0542	0	0.4993	0	0.0241	0.0849	0.0511	0.0045	0.002	0	0.0755	0	0	0.0003	0	0.0758	0.0283	0.0378	0	0.051	0	0.011	<0.0001	0.4911
GSM972433	0	0.0619	0.1383	0	0	0.0686	0	0.0816	0.0057	0.0867	0.0417	0	0	0.1438	0.0259	0.1178	0.0116	0.017	0	0.1609	0.0059	0.0328	<0.0001	0.4565
GSM972432	0	0.0032	0.0791	0.1005	0.0409	0	0.0759	0.0431	0	0	0.0436	0	0	0	0.0283	0.2694	0.1624	0	0	0.1251	0	0.0284	0.02	0.1816
GSM972431	0.0547	0	0.0938	0.0058	0	0.1844	0.073	0.0992	0	0.0759	0.0461	0	0	0.0096	0.0331	0.0928	0.0338	0.037	0	0.1289	0.0319	0	0.001	0.3334
GSM972430	0.0637	0	0.2216	0.0494	0	0.0293	0.0412	0.0702	0.015	0	0.074	0	0	0.0796	0.0379	0.098	0.022	0.0684	0	0.0754	0.0028	0.0514	0.017	0.1905
GSM972429	0.0223	0	0.4524	0.0502	0.011	0.0067	0	0.0323	0.0482	0	0.0478	0	0	0	0.0427	0.0543	0.0516	0	0	0.1474	0.0147	0.0185	<0.0001	0.5781
GSM972428	0	0.0168	0.0911	0.072	0	0.0628	0	0.0682	0.0179	0	0.0584	0	0	0.1306	0.012	0.1065	0.002	0.0346	0	0.2818	0	0.0452	0.001	0.3319
GSM972427	0.0253	0	0.1519	0	0.04	0.2089	0.0982	0	0	0	0.1335	0	0	0.127	0.0244	0.0427	0.0091	0	0	0.1149	0	0.024	0.129	0.1025
GSM972426	0.0332	0	0.2095	0.083	0.0227	0	0.0361	0.0497	0.0485	0.057	0.0306	0	0	0.0902	0.0601	0.0717	0.0244	0	0	0.1618	0.0216	0	<0.0001	0.5585
GSM972425	0	0	0.0794	0.0306	0	0.0492	0	0.0321	0.0383	0	0.0642	0	0	0.3506	0.0231	0.0538	0	0	0	0.2085	0	0.0702	<0.0001	0.4235
GSM972424	0.0394	0	0.0893	0	0.0892	0.1581	0.0624	0	0	0	0.1597	0	0	0.2002	0.0174	0.0011	0.0346	0	0	0.1418	0	0.0069	0.058	0.1387
GSM972423	0.0184	0	0.2221	0.0606	0	0.203	0.0053	0.0639	0.0218	0	0.0908	0	0	0.0924	0.0345	0.0831	0.0136	0.0391	0.0175	0.0339	0	0	0.001	0.315
GSM972422	0.0023	0.0073	0.157	0.0939	0	0	0.035	0.0738	0	0	0.0132	0	0	0.1502	0.1042	0.1949	0.0684	0	0	0.087	0	0.0127	<0.0001	0.6494
GSM972421	0	0.0342	0.0364	0	0	0.0861	0.0536	0.0359	0	0.0078	0.0806	0	0	0.2217	0.038	0.2802	0.0067	0	0.0072	0.0814	0	0.0303	0.005	0.2779
GSM972420	0.0137	0	0.35	0.1539	0	0	0.0611	0.0602	0.0218	0.0454	0	0	0	0	0.0435	0.1094	0.0115	0.0537	0	0.0612	0	0.0146	<0.0001	0.6347
GSM972419	0	0	0.1133	0.0283	0	0.0919	0	0.026	0.0392	0	0.0563	0	0.0002	0.0853	0.0138	0.1489	0.1329	0.0499	0	0.1882	0	0.026	0.001	0.3342
GSM972418	0.0424	0	0.1177	0	0.0235	0.0611	0.1053	0.0875	0.0216	0.0001	0.0641	0	0	0.217	0.0739	0.0713	0.0193	0	0	0.0855	0	0.0097	0.004	0.2969
GSM972417	0	0.0307	0.0222	0	0	0.0022	0.0262	0.0362	0.0355	0.0144	0.0534	0	0	0.5478	0.0165	0.1768	0.0076	0	0	0.0304	0	0	<0.0001	0.6617
GSM972416	0	0.0674	0.0987	0.0531	0	0.0067	0	0.0295	0.0233	0	0.0606	0	0	0.4299	0.008	0.0918	0.0028	0	0	0.1281	0	0	<0.0001	0.5117
GSM972415	0.0476	0	0.3351	0	0.1168	0.0106	0.0792	0.0377	0	0	0.1047	0	0	0.0262	0	0	0.0321	0.0558	0	0.154	0	0	0.008	0.2501
GSM972414	0	0	0.2792	0.005	0	0.1104	0.0232	0.0621	0.0083	0	0.0591	0	0	0.0531	0.0322	0.0496	0.0577	0.0339	0	0.1624	0.0396	0.0243	0.011	0.2198
GSM972413	0.0002	0	0.1688	0.0479	0	0.0455	0	0.0389	0.0103	0.0045	0	0	0	0.3525	0.0271	0.1638	0.0024	0	0	0.1169	0.0009	0.0206	<0.0001	0.4776
GSM972412	0.0398	0	0.4038	0.0604	0	0.0758	0	0.1192	0	0	0.0331	0	0	0.0478	0.0594	0.0721	0.0132	0	0	0.0484	0.0269	0	<0.0001	0.5308
GSM972411	0.051	0	0.1853	0	0.0953	0.0541	0.1284	0.0454	0.0025	0	0.1325	0	0	0.0745	0	0.0568	0.0115	0.0415	0	0.121	0	0	0.017	0.1925
GSM972410	0	0	0.056	0.0155	0	0.0843	0	0.0186	0.0189	0	0.0647	0	0	0.445	0.0393	0.1113	0.0183	0	0	0.1281	0	0	<0.0001	0.423
GSM972409	0.0029	0	0.0927	0.133	0	0.019	0.1021	0.0372	0.0224	0.0198	0.0283	0	0.0021	0.1213	0.1393	0.217	0.0392	0.0132	0.0063	0	0	0.0041	<0.0001	0.7099
GSM972408	0	0.0041	0.0465	0	0.027	0.0388	0	0.0467	0.0009	0.0208	0.0005	0	0	0.1018	0.0056	0.0964	0.0318	0.0518	0	0.3412	0	0.1861	0.001	0.3162
GSM972407	0.0291	0	0.1282	0	0.0468	0.0647	0.0608	0.0172	0.01	0.005	0.0811	0	0	0.3344	0	0.0781	0	0.014	0	0.1306	0	0	0.001	0.3708
GSM972406	0	0	0.1133	0.0253	0	0.0132	0.0618	0.0552	0.0133	0.01	0.0801	0	0	0.219	0.0863	0.1683	0.0089	0.0078	0	0.1179	0	0.0195	<0.0001	0.4269
GSM972405	0	0.0179	0.2593	0.0127	0.0037	0.0408	0.0119	0.0132	0	0.0082	0.0511	0	0	0.1806	0.0175	0.199	0.0107	0.0285	0	0.0914	0.0091	0.0444	0.001	0.37
GSM972404	0.0304	0	0.1965	0.0305	0.0253	0.0825	0	0.0036	0.0313	0.0227	0.0751	0	0	0.0748	0.0444	0.1298	0.0265	0.0096	0	0.1787	0.0364	0.0018	0.001	0.3471
GSM972403	0.0058	0	0.1389	0.0018	0	0.0677	0.0236	0.0288	0.0034	0.0032	0.0358	0	0	0.1833	0.0888	0.1121	0.0076	0	0	0.2332	0	0.066	0.001	0.3729
GSM972402	0.0068	0	0.2451	0.0324	0.0323	0.0474	0	0.0295	0.0311	0	0.0729	0	0	0.1664	0.0267	0.1116	0.0227	0.0248	0	0.1338	0	0.0165	<0.0001	0.4433
GSM972401	0.0086	0	0.1749	0	0	0.0073	0	0.0544	0	0.0189	0.0124	0.0002	0.0535	0.0763	0.0279	0.1094	0	0.0103	0	0.2677	0.0349	0.1433	<0.0001	0.5994
GSM972400	0	0.0121	0.2686	0.0109	0	0.0411	0	0.0576	0.0248	0.0153	0.0045	0.017	0	0.0972	0.0291	0.1675	0.0397	0.0096	0	0.1561	0.0261	0.0228	0.008	0.2504
GSM972399	0	0.0247	0.1723	0.0689	0	0.1228	0.0093	0.0369	0.0388	0	0.0519	0	0	0.102	0.021	0.1346	0.1134	0.026	0	0.0775	0	0	0.003	0.302
GSM972398	0	0	0.2372	0	0	0.2106	0.0736	0.0309	0	0.0133	0.0606	0	0	0.0651	0.0469	0.047	0.0797	0.0121	0	0.0426	0.0039	0.0766	0.004	0.2984
GSM972397	0	0.0483	0.2213	0.0267	0	0.1526	0.0079	0	0.0156	0.0394	0.0976	0	0	0.0482	0.0765	0.1123	0.0425	0.0077	0	0.0762	0.0085	0.0188	0.01	0.2242
GSM972396	0	0.0617	0.1386	0	0	0.3943	0	0	0.0065	0	0.0672	0	0.0194	0.048	0.0268	0.0419	0.0487	0.0463	0	0.1006	0	0	0.123	0.1049
GSM972395	0	0.0247	0.1176	0	0	0.1973	0.05	0.0406	0	0	0.0561	0	0	0.0899	0.1388	0.1064	0.0429	0	0	0.0846	0.0513	0	0.005	0.2718
GSM972394	0.0382	0	0.258	0.0437	0.0202	0	0	0.0666	0.0391	0.0166	0.0883	0	0	0.0682	0.0296	0.144	0.0383	0	0	0.1493	0	0	<0.0001	0.5359
GSM972393	0.0041	0	0.1958	0.0617	0.017	0.0485	0.0282	0.055	0.0037	0	0.0912	0	0.006	0.2075	0.0342	0.0706	0	0.0218	0	0.1546	0	0	0.004	0.2901
GSM972392	0.0206	0	0.2571	0.0738	0	0.0042	0.033	0.0781	0.0204	0	0.0717	0	0	0.1249	0.0511	0.1156	0.0116	0.0211	0	0.1059	0	0.0109	<0.0001	0.413
GSM972391	0	0	0.0638	0.0449	0	0.1382	0.0355	0.0014	0	0	0.055	0.0134	0	0.2264	0.0406	0.0985	0.0027	0.0423	0	0.2063	0	0.031	0.001	0.3412
GSM972390	0.0469	0	0.4403	0.1123	0.026	0	0	0.0355	0.06	0	0.0667	0	0	0.0316	0.004	0.0414	0.0102	0	0	0.1114	0	0.0138	<0.0001	0.5983
GSM972389	0.062	0	0.1534	0	0	0.2259	0.0814	0	0.0214	0.0188	0.0722	0	0	0.1056	0.1143	0.0655	0.0498	0	0	0.0296	0	0	0.004	0.2892
GSM972388	0	0.0182	0.025	0	0	0.1268	0.1621	0	0	0	0.1867	0	0.0342	0.1472	0.0641	0.0014	0.0221	0.0536	0	0.1585	0	0	0.054	0.14
GSM972387	0.0274	0	0.2028	0.0176	0.0097	0.1596	0.1036	0.0101	0	0	0.0943	0	0	0.129	0.0292	0.0112	0.0039	0.0517	0	0.1185	0	0.0314	0.019	0.1835
GSM972386	0	0	0.2914	0.0243	0	0.0963	0	0.0326	0.0247	0	0.0908	0	0	0.0612	0.0289	0.172	0.0469	0.005	0	0.1135	0.0122	0	<0.0001	0.3923
GSM972385	0.0284	0	0.1359	0.0839	0.0345	0	0.0315	0.0513	0.0155	0.0519	0.023	0	0	0.2512	0.0595	0.1648	0	0.0095	0	0.0507	0	0.0082	<0.0001	0.548
GSM972384	0.0132	0	0.0923	0	0.0012	0.0951	0	0.0468	0.021	0	0.0588	0	0	0.0032	0.0163	0.0909	0.0355	0.0724	0	0.2914	0.0515	0.1104	0.004	0.2877
GSM972383	0.0123	0	0.4405	0.041	0.0168	0.0779	0.0039	0	0.0187	0	0.0651	0	0	0.0417	0.0178	0.1244	0.0467	0.0182	0.0438	0.0312	0	0	<0.0001	0.5184
GSM972382	0.0228	0	0.0422	0	0.0444	0.0566	0.0963	0.0197	0.0078	0	0.1068	0	0	0.4112	0.0344	0.0308	0.0066	0.0267	0	0.0936	0	0	0.001	0.3145
GSM972381	0	0.0568	0.0645	0.033	0	0	0	0.0126	0.0656	0	0.0278	0	0	0.3801	0	0.2531	0	0	0	0.0847	0	0.0218	0.001	0.3334
GSM972380	0.0039	0.0093	0.267	0.0211	0	0.0571	0.0316	0.133	0	0.0162	0.0598	0	0	0.0696	0.1041	0.0969	0.0062	0.0254	0	0.0657	0	0.0332	<0.0001	0.6422
GSM972379	0	0	0.3304	0.1212	0	0.1391	0.0344	0.0172	0.0193	0	0.0678	0	0	0.0496	0.0248	0.1116	0	0.017	0	0.0677	0	0	0.001	0.3194
GSM972378	0.0144	0	0.1879	0	0.0426	0.0347	0	0	0.0229	0.0122	0.0837	0	0	0.275	0.0042	0	0	0.015	0	0.2655	0	0.042	<0.0001	0.3861
GSM972377	0.0098	0	0.1505	0.0084	0.0155	0.016	0.0155	0.0496	0.0502	0	0.0735	0	0	0.2804	0.0678	0.1179	0	0.0131	0	0.1059	0	0.0258	<0.0001	0.3815
GSM972376	0.0333	0	0.0005	0	0.0367	0.0569	0.0877	0.017	0	0	0.1143	0	0	0.393	0.0592	0.0839	0	0	0	0.1175	0	0	0.014	0.2057
GSM972375	0.0449	0	0.1128	0.0765	0	0.1216	0	0.0063	0.0145	0	0.0987	0	0	0.07	0.0624	0.153	0.0509	0.0433	0	0.0892	0	0.0559	0.001	0.3252
GSM972374	0.0526	0	0.267	0.0555	0	0.0175	0.0447	0.0862	0.027	0.0134	0.0537	0	0	0.0238	0.1056	0.1468	0.0228	0.0009	0	0.0824	0	0	<0.0001	0.5032
GSM972373	0	0.1201	0.065	0.0165	0.0751	0.0789	0.069	0.0112	0	0	0.0972	0	0	0.1896	0.023	0.0802	0.0441	0.0389	0	0.0912	0	0	0.232	0.0711
GSM972372	0	0	0.2812	0.0134	0	0.0631	0.0085	0.0693	0.0065	0	0.0283	0	0	0.0751	0.0698	0.1499	0.038	0.0052	0	0.1504	0.0021	0.0392	0.001	0.3612
GSM972371	0.0551	0.0096	0.0744	0.0313	0.014	0.0674	0	0.1177	0	0	0.1057	0	0	0.205	0.0138	0.1116	0.0308	0.0112	0	0.1318	0.0206	0	0.011	0.2125
GSM972370	0.0101	0	0.2908	0.0545	0.0384	0.0394	0.0666	0.0216	0.0246	0	0.0731	0	0	0.0766	0.0424	0.1211	0.0282	0	0	0.1128	0	0	<0.0001	0.5045
GSM972369	0.0021	0	0.0372	0.0386	0	0.0003	0.0093	0.0328	0.0166	0	0	0.0233	0	0.2233	0.0472	0.1659	0.0222	0	0	0.2658	0.0013	0.1141	0.004	0.2909
GSM972368	0.0383	0	0.1725	0.0899	0	0.0296	0.1051	0.0425	0	0	0.1111	0	0	0.1187	0.0182	0.0679	0.0054	0.0251	0	0.163	0	0.0126	0.018	0.1879
GSM972367	0.0136	0	0.2543	0.0561	0	0.0798	0.0086	0.027	0.0323	0	0.0288	0	0	0.2409	0.0474	0.1218	0.0168	0	0	0.0708	0	0.0018	<0.0001	0.5132
GSM972366	0.0138	0	0.1721	0.0897	0	0.2459	0	0.0889	0.0113	0	0.0435	0	0.0095	0.0051	0.0264	0.1413	0.0713	0.0031	0	0.0664	0.0087	0.0029	0.035	0.1638
GSM972365	0.0556	0	0.1251	0.0175	0	0.0638	0.0036	0.0628	0	0	0.0629	0	0.0208	0.1713	0.0513	0.0954	0.03	0.0158	0	0.1627	0.031	0.0303	<0.0001	0.3925
GSM972364	0.0336	0	0.0897	0.0461	0	0.1605	0	0.0114	0.0387	0	0.149	0	0	0.0769	0.0096	0.1574	0	0.0225	0	0.2047	0	0	0.026	0.1769
GSM972363	0	0	0.1517	0.1023	0	0	0	0.0678	0.0222	0	0.0895	0	0	0.1991	0.06	0.1293	0.0386	0.0155	0	0.0788	0	0.0452	0.004	0.2922
GSM972362	0.0304	0	0.3633	0.0489	0	0.1527	0.0422	0.0616	0.0057	0	0.0651	0	0	0.01	0.0251	0.0758	0.0165	0.033	0	0.0696	0	0	<0.0001	0.5108
GSM972361	0	0.0084	0.1415	0.1874	0	0	0.1013	0.0556	0.0017	0.1197	0	0.0107	0	0.024	0.1372	0.178	0.0214	0	0	0.0123	0	0.0008	<0.0001	0.6358
GSM972360	0	0	0.0547	0.1138	0	0.058	0	0.1268	0.0204	0	0.1064	0.0146	0	0.0056	0.1466	0.2225	0.0734	0.0277	0.027	0.0026	0	0	0.001	0.3505
GSM972359	0.036	0	0.2567	0.0163	0	0.1123	0	0.0594	0.0068	0.0234	0.0745	0	0	0	0.0352	0.1312	0.1037	0	0.0535	0.0608	0	0.0302	<0.0001	0.501
GSM972358	0.0125	0	0.2709	0	0	0.0933	0.01	0.0541	0.0342	0	0.046	0	0	0.1343	0.0505	0.1214	0.044	0.0193	0	0.0667	0.0337	0.0092	<0.0001	0.4257
GSM972357	0	0.0166	0.3353	0.0769	0	0.0682	0	0.0874	0.0311	0	0.1016	0.0033	0	0	0.0128	0.1441	0.0309	0.023	0	0.068	0	0.0008	0.118	0.106

CIBERSORT scores
0.5
0.4
0.3
0.2
0.1
0.0

Supplementary Figure S1. Continued

Input Samples	B cells naive	B cells memory	Plasma cells	T cells CD8	T cells CD4 naive	T cells CD4 memory resting	T cells CD4 memory activated	T cells follicular helper	T cells regulatory (Tregs)	T cells gamma delta	NK cells resting	NK cells activated	Monocytes	Macrophages M0	Macrophages M1	Macrophages M2	Dendritic cells resting	Dendritic cells activated	Mast cells resting	Mast cells activated	Eosinophils	Neutrophils	P-value	Pearson correlation
GSM972522	0	0.0295	0.2309	0.0761	0	0.0928	0.0156	0.0327	0.0169	0.0005	0.0639	0	0	0.1178	0.072	0.1316	0.0403	0.0023	0	0.0722	0	0.005	<0.0001	0.4489
GSM972521	0.0373	0	0.1012	0.0538	0	0	0.0944	0.099	0	0.0227	0.0632	0	0	0.1279	0.1212	0.1997	0.0166	0.0116	0	0.0493	0	0.002	<0.0001	0.4186
GSM972520	0.0147	0	0.2555	0	0	0.1681	0.0305	0.0457	0	0.0028	0.0346	0	0	0.0561	0.0681	0.1281	0.0175	0.0192	0	0.0946	0.0299	0.0347	<0.0001	0.4003
GSM972519	0	0	0.2373	0.0873	0	0.0211	0	0.0589	0.0259	0	0.0561	0	0	0.068	0.0137	0.1233	0.0061	0.0263	0	0.2309	0.0104	0.0345	<0.0001	0.4084
GSM972518	0.0186	0	0.222	0.0712	0	0	0	0.0906	0.0272	0.0024	0.0089	0	0	0.0921	0.0557	0.2047	0.0786	0	0	0.073	0	0.0552	<0.0001	0.5358
GSM972517	0.0371	0	0.2171	0	0.0228	0.1325	0.044	0.0263	0	0	0.1029	0	0	0.0629	0.0634	0.1232	0.0481	0	0	0.0967	0.0047	0.0183	0.004	0.2966
GSM972516	0	0.0498	0.0886	0.2007	0	0	0.0854	0.063	0.0641	0	0.0706	0	0.0104	0.0806	0.0567	0.0518	0.0775	0	0	0.101	0	0	0.08	0.1252
GSM972515	0	0.0364	0.1792	0	0	0.2106	0.0206	0.0097	0.0255	0	0.1108	0	0	0.0017	0.0223	0.1001	0.0509	0.0819	0	0.1322	0	0.0181	0.071	0.1297
GSM972514	0	0.0465	0.0853	0	0	0.1858	0.0436	0.0902	0.003	0	0.0526	0	0	0.0529	0.0846	0.1193	0.1074	0.0144	0	0.0782	0.0048	0.0315	0.018	0.1884
GSM972513	0.0146	0	0.1114	0.0359	0	0.1052	0.0225	0.0308	0.0196	0	0.0869	0	0	0.0716	0.0384	0.2718	0.0338	0.0363	0	0.1015	0	0.0197	0.039	0.1587
GSM972512	0.0531	0	0.3004	0	0	0.0719	0	0.0786	0.0348	0.0025	0.0591	0	0	0.1042	0.0052	0.0828	0	0.0535	0	0.1385	0.0153	0	0.001	0.3145
GSM972511	0	0.0719	0.0958	0	0.0243	0.137	0.0562	0.0263	0	0.0306	0.0458	0	0	0.0339	0.0679	0.162	0.1047	0	0	0.1216	0.0157	0.0063	0.006	0.2686
GSM972510	0.044	0	0.2929	0.0292	0	0.1241	0	0.0183	0.0513	0.0128	0.0452	0	0.0145	0	0	0.1587	0.0267	0.0351	0	0.1472	0	0	<0.0001	0.3967
GSM972509	0.0511	0	0.1348	0	0.0478	0	0.1095	0.0624	0.044	0	0.0948	0	0	0.0861	0.0545	0.0605	0.0519	0.0461	0	0.1068	0.0497	0	0.015	0.2014
GSM972508	0.037	0	0.2756	0.0309	0	0.1156	0.0777	0.0534	0	0.0113	0.0656	0	0.0029	0.0511	0.0513	0.0775	0.0125	0.0357	0	0.0892	0.0124	0	<0.0001	0.4191
GSM972507	0.0619	0	0.261	0.0355	0	0.073	0	0.0987	0.0057	0.0248	0	0.0284	0.013	0.0292	0.1376	0.1218	0.0317	0.0236	0.0182	0.0359	0	0	<0.0001	0.4399
GSM972506	0	0.0057	0.2368	0.0855	0	0.0742	0.0036	0.0569	0.0028	0.0116	0.0336	0	0	0.0383	0.0307	0.1655	0.0744	0.003	0	0.1557	0	0.0216	<0.0001	0.5785
GSM972505	0.0137	0	0.3478	0.0745	0	0.0136	0.0069	0.0291	0.0165	0	0.0994	0	0	0.1759	0.0236	0.0567	0	0.0241	0	0.1181	0	0	<0.0001	0.5276
GSM972504	0	0.0212	0.1919	0.0894	0	0	0.0084	0.1143	0.0121	0	0.0609	0	0	0.2217	0.0406	0.1478	0	0	0	0.0879	0	0.0037	<0.0001	0.5256
GSM972503	0.0971	0	0.2751	0.1279	0	0	0.0218	0.1165	0.022	0	0.0559	0	0	0.0467	0.0128	0.1539	0	0.0225	0	0.0322	0.003	0.0126	<0.0001	0.556
GSM972502	0	0	0.2781	0.0339	0.1289	0.0089	0	0.0216	0.0096	0	0.0592	0	0	0.0535	0.01	0.1569	0.0975	0.0195	0	0.1225	0	0	0.004	0.2888
GSM972501	0.0262	0	0.3706	0.0705	0.0082	0	0.027	0.0412	0.02	0.0315	0.0242	0	0	0.1251	0.0194	0.066	0.0073	0.0213	0	0.1282	0	0.0132	<0.0001	0.6469
GSM972499	0	0.007	0.2203	0.0883	0	0	0.0338	0.0552	0.0249	0	0.0245	0.0047	0	0.0435	0.0907	0.0766	0	0.1267	0	0.1396	0	0.0641	<0.0001	0.4557
GSM972498	0.0234	0	0.0991	0	0	0.2649	0	0.0018	0.0457	0	0.1182	0	0	0.0657	0.0273	0.0677	0.1279	0.0511	0.0218	0.0684	0	0.0169	0.1	0.1124
GSM972497	0.0208	0	0.243	0.0049	0	0.0648	0.0268	0.037	0.0348	0	0.076	0	0	0.2893	0.032	0.0885	0.041	0	0	0.0414	0	0	<0.0001	0.4094
GSM972496	0.0318	0	0.2916	0.0725	0	0.1613	0.0148	0.0865	0	0	0.0737	0	0	0.0255	0.0291	0.0595	0.0067	0.0351	0	0.1119	0	0	0.003	0.3048
GSM972495	0	0	0.094	0.098	0	0.0657	0.0781	0.0698	0	0	0.0763	0	0.0076	0.0553	0.1022	0.0744	0.2007	0.0021	0	0.076	0	0	0.005	0.2726
GSM972494	0	0.0314	0.1149	0.0785	0	0	0.0351	0.1482	0.0346	0.1001	0.0141	0	0	0.0646	0.1492	0.0507	0.0639	0.0337	0	0.081	0	0	<0.0001	0.4931
GSM972493	0.0334	0	0.1688	0.0676	0	0.1068	0.0321	0.0675	0.0042	0	0.0924	0	0	0.1148	0.0829	0.115	0.0285	0.0279	0	0.0543	0	0.0039	0.009	0.2352
GSM972492	0.0886	0	0.081	0	0	0.0131	0.1178	0.0775	0.0024	0	0.1452	0.0116	0.0084	0	0.025	0.2062	0.0404	0.0533	0	0.1206	0	0.0091	0.004	0.2863
GSM972491	0	0.0024	0.0971	0.0364	0	0.0696	0	0.0473	0.0112	0	0.078	0	0	0.1539	0.0577	0.172	0.0533	0	0	0.1775	0	0.0436	0.008	0.2401
GSM972490	0.066	0	0.1171	0	0.0014	0.041	0.0531	0.0359	0.0425	0.0333	0.1183	0	0	0.1338	0.0285	0.0445	0.0081	0.0035	0	0.2335	0	0.0395	0.009	0.2353
GSM972489	0	0	0.3651	0.0395	0	0.1095	0	0.0379	0.0104	0.0395	0.0529	0	0	0.051	0.0304	0.1707	0.0003	0.0189	0	0.0712	0	0.0027	<0.0001	0.5104
GSM972488	0.011	0	0.4629	0	0	0.1385	0	0.0231	0.0245	0.0243	0.0413	0	0	0.0567	0.0057	0.0911	0.0091	0.0169	0	0.0949	0	0	<0.0001	0.6029
GSM972487	0.0391	0	0.2264	0	0.0134	0.2027	0	0.0129	0.0229	0	0.0989	0	0	0.0984	0	0.0708	0	0.0359	0	0.1639	0	0.0148	0.009	0.2321
GSM972486	0	0	0.1051	0.0059	0.0125	0.0422	0.0425	0.0233	0.021	0	0.0599	0	0	0.3648	0.034	0.1212	0.0012	0.0059	0	0.1449	0	0.0155	<0.0001	0.5427
GSM972485	0.0395	0	0.1838	0.0068	0.063	0.0364	0.0727	0.0543	0.0066	0.0046	0.1075	0	0	0.1523	0.0342	0.1028	0.0028	0.0445	0	0.0882	0	0	0.001	0.3302
GSM972484	0.0219	0	0.048	0.0529	0	0.0362	0.0201	0.0936	0.0152	0	0.0588	0	0.0312	0.1422	0.1561	0.1477	0.0326	0	0	0.1433	0	0	0.001	0.3622
GSM972483	0	0	0.1267	0	0.0497	0.1763	0.0408	0.0093	0	0	0.0902	0	0.0262	0.0874	0.0619	0.2315	0.0836	0.0003	0.0002	0.0159	0	0	0.057	0.1392
GSM972482	0	0	0.114	0.0151	0	0.1661	0	0.0122	0.004	0	0.0744	0	0	0.1599	0.0278	0.2882	0.0179	0.0105	0	0.0938	0	0.0161	0.006	0.2677
GSM972481	0.0202	0	0.3328	0.0301	0	0.0833	0.0092	0.0203	0.0163	0	0.078	0	0	0.0776	0.0299	0.1426	0.047	0.0088	0	0.0916	0.0046	0.0077	<0.0001	0.5033
GSM972480	0	0.0235	0.2318	0.0844	0	0.1256	0	0.0519	0	0	0.0894	0	0	0	0.0458	0.1801	0.0133	0.0425	0	0.1084	0	0.0034	0.001	0.3428
GSM972479	0.0009	0	0.0838	0.0187	0	0	0	0.0447	0.0168	0.0098	0	0.0042	0	0.3257	0.0142	0.2166	0	0	0	0.2225	0	0.0422	<0.0001	0.6202
GSM972478	0.0272	0	0.3075	0.027	0	0.122	0	0.0757	0	0	0.0423	0	0	0.0553	0.0416	0.0837	0.0192	0.046	0	0.1324	0.0201	0	<0.0001	0.4875
GSM972477	0.0083	0	0.2595	0.1278	0	0.0149	0	0.0976	0.0334	0.0101	0.0584	0	0.0221	0	0.0779	0.0356	0.059	0.0291	0	0.1349	0.0313	0	0.005	0.2728
GSM972476	0.0158	0	0.1141	0.0611	0	0.0055	0	0.0382	0.0365	0	0.0145	0	0	0.252	0.0618	0.1875	0.0675	0	0	0.0784	0.0276	0.0395	0.001	0.36
GSM972475	0	0	0.2168	0.0451	0	0.2227	0	0	0.0433	0	0.0232	0	0	0.1853	0.0226	0.1292	0.0302	0	0	0.0816	0	0	0.001	0.3356
GSM972474	0.0305	0	0.2537	0.0318	0	0.0323	0.0225	0.0673	0.0354	0	0.0559	0	0	0.1147	0.085	0.1279	0.0089	0.0424	0.0454	0.0042	0.0131	0.0289	<0.0001	0.4769
GSM972473	0.0125	0	0.2215	0.0568	0	0.0729	0.0765	0.0427	0	0	0.0633	0	0	0.025	0.0326	0.1142	0.0541	0.0403	0	0.1485	0.0104	0.0288	<0.0001	0.4145
GSM972472	0.0232	0	0.1121	0	0.0198	0.0924	0	0.001	0.0197	0	0.0453	0	0	0.0984	0.0116	0.1156	0.0249	0.0077	0	0.3415	0.0129	0.0738	<0.0001	0.4071
GSM972470	0.0008	0	0.1956	0.0532	0	0	0.0455	0.0407	0	0.1285	0	0	0	0.0827	0.087	0.1993	0.0132	0	0	0.1077	0	0.0457	<0.0001	0.535
GSM972469	0	0.0192	0.3224	0.0043	0	0.0766	0.0034	0.0663	0.0346	0	0.0648	0	0	0.0787	0.0274	0.1245	0.0334	0.033	0	0.0946	0	0.0167	<0.0001	0.4932
GSM972468	0	0	0.1885	0	0	0.0937	0.028	0.0666	0.0124	0.0109	0.0575	0	0	0.1472	0.1103	0.094	0.0275	0.0107	0	0.1228	0	0.03	0.003	0.3029
GSM972467	0.0182	0	0.2645	0.0198	0.0169	0.0192	0.0455	0.0996	0	0.0044	0.0605	0	0	0.0908	0.1178	0.1412	0.0497	0	0.0145	0.0374	0	0	0.001	0.3446
GSM972466	0.0289	0	0.0863	0.1077	0	0	0.0285	0.0922	0.0817	0	0.0944	0	0	0.0376	0.0533	0.0638	0	0.0877	0	0.1787	0	0.0592	0.008	0.239
GSM972465	0	0.0131	0.0971	0.0283	0.0588	0.0952	0	0	0.01	0	0.0477	0	0	0.274	0.0305	0.1235	0	0.0225	0	0.1838	0	0.0155	0.004	0.2893
GSM972464	0.1745	0	0.1648	0.0264	0.0041	0.0432	0.0489	0.0779	0	0	0.0861	0	0	0.0228	0.0197	0.1257	0.0734	0.0153	0	0.0897	0.0166	0.0109	0.001	0.3315
GSM972463	0	0	0.3725	0.0934	0	0.1545	0	0.0407	0.0121	0	0.0532	0	0	0.0283	0.0134	0.1289	0.0042	0.0363	0	0.0626	0	0	<0.0001	0.4209
GSM972462	0	0.0372	0.1621	0.1275	0.0231	0.0523	0.1257	0.0245	0	0	0.0649	0	0	0.1399	0.1013	0.0126	0.0446	0.0355	0	0.0489	0	0	0.004	0.2992
GSM972461	0.0526	0	0.1144	0.1062	0	0	0	0.1037	0.046	0.0225	0	0.0186	0	0.1619	0.1554	0.0321	0.0603	0	0	0.1232	0.003	0	0.008	0.2462
GSM972460	0	0.0206	0.2465	0.0484	0	0.0523	0.037	0.1112	0.0127	0	0.0212	0	0	0.0586	0.0772	0.1656	0.0803	0.0301	0.0022	0.0257	0	0.0103	<0.0001	0.3965
GSM972459	0.0372	0	0.2953	0	0.0509	0.0856	0.1022	0	0	0	0.0857	0	0	0	0.0644	0.1312	0.0116	0.0191	0	0.0726	0	0.0443	<0.0001	0.3919

CIBERSORT scores
0.4
0.3
0.2
0.1
0.0

Supplementary Figure S1. Quantification of immune cell infiltration via CIBERSORT analysis of gene expression data from tumor samples of 562 patients in GSE39582. The heatmap displays the infiltration abundance of 22 immune cell subtypes in each sample. The color intensity shown in the right column is proportional to the CIBERSORT score. Corresponding *p*-values and Pearson correlation coefficients were also demonstrated. CIBERSORT： Cell-type Identification by Estimating Relative Subsets of RNA Transcripts.

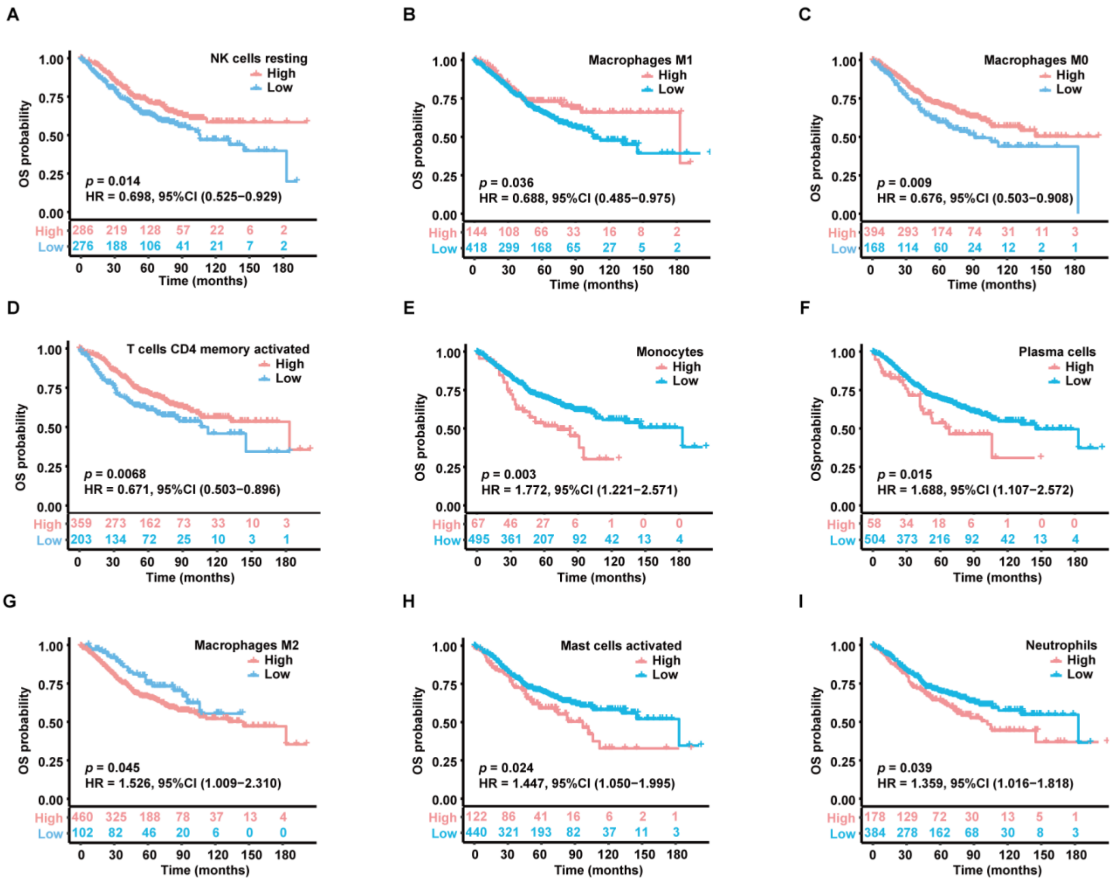

Supplementary Figure S2. KM analysis of immune cells significantly associated with CC prognosis, except for aDCs. Protective cell types include resting NK cells (**A**), M1-type macrophages (**B**), M0-type macrophages (**C**) and activated CD4+ memory T cells (**D**); whereas risk cell types encompass monocytes (**E**), plasma cells (**F**), M2-type macrophages (**G**), activated mast cells (**H**), and neutrophils (**I**). 562 patients were categorized into the high- and low-infiltration groups according to the optimal cutoff values of the CIBERSORT scores of each cell type. Numbers of individuals alive at different time points are labeled at the bottom. KM: Kaplan-Meier; CC: colon cancer; NK: natural killer.

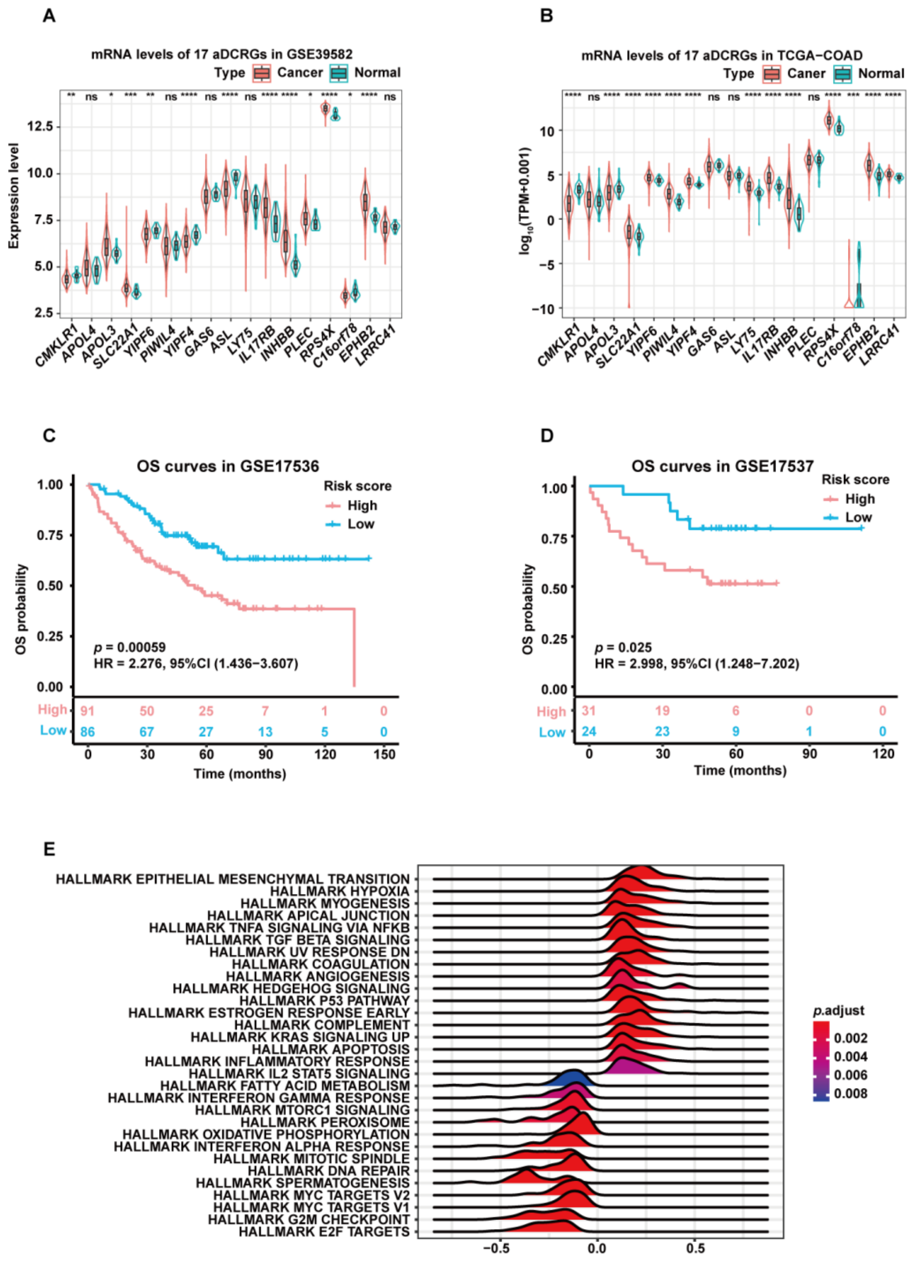

Supplementary Figure S3. Evaluation of mRNA levels of aDCRGs, KM analysis and GSEA. (**A,B**) Violin plots of the mRNA expression of 17 aDCRGs in tumor tissues and adjacent normal tissues based on the training set (**A**) and the second validation set (**B**). In the training set, statistical significance was determined by student's *t*-test for genes *YIPF6* and *YIPF4*, or by Wilcoxon-Mann-Whitney test for other genes. In the second validation set, statistical significance was determined by student's *t*-test for the gene *RPS4X*, or by Wilcoxon-Mann-Whitney test for other genes. *$p < 0.05$; **$p < 0.01$; ***$p < 0.001$; ****$p < 0.0001$. ns： not significant. (**C,D**) KM survival curves of patients in the high- and low-risk groups based on the subset GSE17536 (**C**) and subset GSE17537 (**D**). Numbers of individuals alive at different time points are presented at the bottom. (**E**) GSEA based on hallmark gene sets elucidated the differences in enriched gene sets between the high- and low-risk groups in the training set. Enrichment results of 30 gene sets that exhibit significant differences between the two groups are demonstrated. A total of 17 gene sets with ES more than 0 are enriched in the high-risk group, while 13 gene sets with ES less than 0 are enriched in the low-risk group. Color intensity is proportional to the *p*-value as demonstrated in the right column.

aDCRG: aDC-related prognostic gene; GSEA: Gene Set Enrichment Analysis; OS: overall survival; ES: enrichment score.

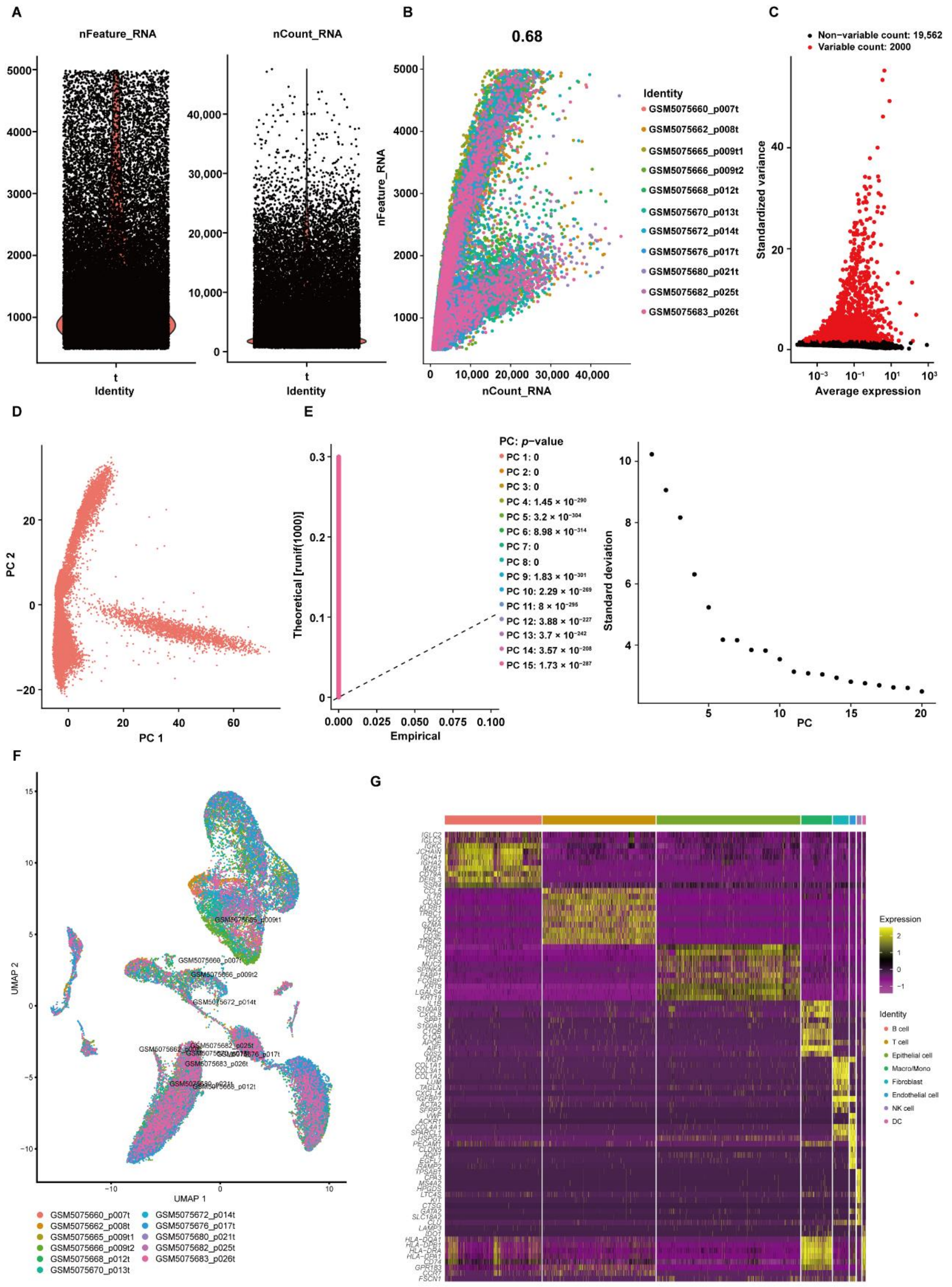

Supplementary Figure S4. Processing of scRNA-seq data. (A) Number of genes detected in each cell (nFeature_RNA) and total number of molecules detected within a cell (nCount_RNA). (B) The numbers of detected genes were significantly associated with the sequencing depth, with a Pearson's correlation coefficient of 0.68. (C) A variance diagram demonstrating 21,562 corresponding genes throughout all cells, with the red dots denoting highly variable genes and the black dots signifying non-variable genes. (D) Dimensionality reduction of all cells using PCA. (E) PCA screened out the top 15 PCs with an estimated

p-value < 0.05. (F) The UMAP plot of cells from 11 tumor tissues of 10 CC patients. (G) A heatmap exhibiting the top 10 marker genes of each cell cluster. The colors from purple to yellow correspond to levels of gene expression from low to high. ScRNA-seq: Single-cell RNA sequencing; PCA: principal component analysis; PC: principal component; UMAP: uniform manifold approximation and projection; Macro/Mono: Macrophage/Monocyte.

1.2 Supplementary Tables

Table S1. Information of 67 CC patients in the TCGA-COAD dataset collected and curated by the CTR-DB.

CTR-DB ID	Therapeutic Regimen	Response		Non-response	
		CR	PR	PD	SD
CTR_RNAseq_10	Cetuximab+Fluorouracil+Leucovorin+Oxaliplatin	1	0	0	1
CTR_RNAseq_117	Capecitabine	6	1	1	1
CTR_RNAseq_133	Bevacizumab+Fluorouracil+Irinotecan+Leucovorin	1	0	2	1
CTR_RNAseq_136	Fluorouracil	0	1	0	0
CTR_RNAseq_146	Capecitabine+Oxaliplatin	1	0	1	0
CTR_RNAseq_186	Bevacizumab+Leucovorin	1	0	0	0
CTR_RNAseq_190	Fluorouracil+Leucovorin	1	0	0	0
CTR_RNAseq_232	Fluorouracil+Irinotecan+Leucovorin	2	0	1	0
CTR_RNAseq_250	Fluorouracil+Irinotecan+Leucovorin	0	0	1	0
CTR_RNAseq_29	Fluorouracil+Leucovorin+Oxaliplatin	0	0	3	1
CTR_RNAseq_296	Bevacizumab+Fluorouracil+Irinotecan+Leucovorin	0	0	1	0
CTR_RNAseq_306	Capecitabine	1	0	0	0
CTR_RNAseq_308	Fluorouracil+Leucovorin	3	1	0	0
CTR_RNAseq_346	Fluorouracil+Leucovorin+Oxaliplatin	21	1	1	0
CTR_RNAseq_394	Bevacizumab+Fluorouracil+Oxaliplatin	1	1	0	0
CTR_RNAseq_468	Bevacizumab+Fluorouracil+Leucovorin+Oxaliplatin	0	0	2	0
CTR_RNAseq_49	Fluorouracil	0	1	0	0
CTR_RNAseq_70	Fluorouracil+Leucovorin+Oxaliplatin	2	0	1	0
CTR_RNAseq_8	Capecitabine+Oxaliplatin	1	0	1	0

CTR-DB: Cancer Treatment Response gene signature DataBase; RNA-seq: RNA sequencing; CR: Complete Response; PR: Partial Response; PD: Progressive Disease; SD: Stable Disease.
